# dTAF10- and dTAF10b-Containing Complexes Are Required for Ecdysone-Driven Larval-Pupal Morphogenesis in *Drosophila melanogaster*

**DOI:** 10.1371/journal.pone.0142226

**Published:** 2015-11-10

**Authors:** Zoltan Pahi, Zsuzsanna Kiss, Orbán Komonyi, Barbara N. Borsos, Laszlo Tora, Imre M. Boros, Tibor Pankotai

**Affiliations:** 1 Department of Biochemistry and Molecular Biology, University of Szeged, Szeged, Hungary; 2 Institute of Biochemistry, Biological Research Center, Szeged, Hungary; 3 Institut de Genetique et de Biologie Moleculaire et Cellulaire, Illkirch, France; Saint Louis University School of Medicine, UNITED STATES

## Abstract

In eukaryotes the TFIID complex is required for preinitiation complex assembly which positions RNA polymerase II around transcription start sites. On the other hand, histone acetyltransferase complexes including SAGA and ATAC, modulate transcription at several steps through modification of specific core histone residues. In this study we investigated the function of *Drosophila melanogaster* proteins TAF10 and TAF10b, which are subunits of dTFIID and dSAGA, respectively. We generated a mutation which eliminated the production of both Drosophila TAF10 orthologues. The simultaneous deletion of both d*Taf10* genes impaired the recruitment of the dTFIID subunit dTAF5 to polytene chromosomes, while binding of other TFIID subunits, dTAF1 and RNAPII was not affected. The lack of both dTAF10 proteins resulted in failures in the larval-pupal transition during metamorphosis and in transcriptional reprogramming at this developmental stage. Surprisingly, unlike dSAGA mutations, dATAC subunit mutations resulted in very similar changes in the steady state mRNA levels of approximately 5000 genes as did ablation of both d*Taf10* genes, indicating that dTAF10- and/or dTAF10b-containing complexes and dATAC affect similar pathways. Importantly, the phenotype resulting from d*Taf10+*d*Taf10b* mutation could be rescued by ectopically added ecdysone, suggesting that dTAF10- and/or dTAF10b-containing complexes are involved in the expression of ecdysone biosynthetic genes. Indeed, in d*Taf10+*d*Taf10b* mutants, cytochrome genes, which regulate ecdysone synthesis in the ring gland, were underrepresented. Therefore our data support the idea that the presence of dTAF10 proteins in dTFIID and/or dSAGA is required only at specific developmental steps. We propose that distinct forms of dTFIID and/or dSAGA exist during Drosophila metamorphosis, wherein different TAF compositions serve to target RNAPII at different developmental stages and tissues.

## Introduction

Eukaryotic transcription is a well-controlled multistep process because transcriptional programming is critical for growth, development, and survival. For tight regulation of the transcription of RNA polymerase II (RNAPII)-dependent genes, the coordination of cascade events is required. This involves the binding of activators to enhancers, the assembly of the transcription preinitiation complex (PIC) at promoter regions, and finally RNAPII initiation and elongation [1]. During transcriptional activation, PIC assembly is tightly regulated and involves large multiprotein complexes such as TFIIs, RNAPII, and chromatin modifiers. For transcription initiation, the presence of basal transcription factors such as TFIID, TFIIA, TFIIB, TFIIF, TFIIE, and TFIIH is required [2]. These factors are recruited onto core promoters of protein coding genes for the assembly of the PIC [3]. The TFIID complex, which plays an essential role in promoter recognition, is composed of 14 subunits: the TBP (TATA-binding protein) and 13 TAFs (TBP-associated factors) [4]. The TFIID is a key regulator of PIC assembly to core promoters and targets its binding around the transcription start site with the help of TBP [5]. Individual TAF subunits can also associate to the core promoter in cooperation with TATA-bound TBP and enhance the assembly of other general transcription factors at developmentally regulated gene promoters, leading to functional PIC assembly and RNAPII transcription initiation.

In yeast, the TFIID complex is composed of six TAFs (TAF4, TAF5, TAF6, TAF9, TAF10, and TAF12), which are present in double copies, while seven TAFs and TBP are present in a single copy [6, 7]. The duplicated TAFs create a symmetric scaffold and the remaining TAFs and TBP localize at the periphery of TFIID. *In vivo* studies highlight that both functional Drosophila and human core TFIID complexes contain dTAF4, dTAF5, dTAF6, dTAF9, and dTAF12 in their central regions [8]. The TAF8-TAF10 heterodimer is present in one copy in the human TFIID core complex (called 7TAF) [9–11]. After the binding of TAF8–TAF10 to the TAF4, TAF5, TAF6, TAF9, and TAF12-containing TFIID core complex, conformational change occurs inside the TFIID [9–11]. Interestingly, both TAF10- and TAF2-lacking TFIID complexes have been described from human cells [12–14].

TAFs are also present in the Spt-Ada-Gcn5 histone acetyltransferase (SAGA HAT) complex [15]. In mammalian cells, TAF10 is present in TFIID and SAGA-type complexes [16–19]. SAGA complexes contain the GCN5 HAT enzyme, as well as SPT, TRRAP, and ADA proteins. Additionally, several TAFs are also subunits of these complexes [20]. Interestingly, in Drosophila two dTAF10 homologues (dTAF10 and dTAF10b) have been identified [21, 22]. *Drosophila melanogaster* has evolved to separate dTAF10 functions by expressing two different dTAF10 homologues: dTAF10 is a dTFIID subunit and dTAF10b is a dSAGA subunit [23].

Data on the specific functions of individual TAF proteins, and in particular, that of TAF10 from different metazoan species in different experimental systems and distinct developmental stages showed that TAF10 has general or cell-specific roles in gene expression. A *TAF10* mutation in yeast affected the transcription of various genes and caused morphological changes and cell cycle-dependent phenotypes [24, 25]. RNAi experiments showed that TAF10 was necessary for the transcription of a set of specific genes in *Caenorhabditis elegans* early embryo, but not required for the expression of several genes in worm development [26]. The loss of TAF10 in cultured mouse cells resulted in cell cycle arrest and apoptotic cell death [27]. The mouse *Taf10* gene affected the fitness of pluripotent cells and was therefore shown to be essential in early embryonic development [28]. Inactivation of TAF10 in mouse F9 cells dissociated the TFIID complex to TBP and a TAF-containing subcomplex [28], while in developing mouse liver cells, TAF10 ablation resulted in the complete disassembly of TFIID complex into individual subunits [29]. The silencing of TAF10 in embryonic keratinocytes perturbed the expression of certain genes and affected keratinocyte differentiation. In contrast, the elimination of TAF10 in adult keratinocytes did not cause expressional changes in any of the tested genes and did not affect the response to ultraviolet (UV) irradiation or skin regeneration after wounding [30]. Similarly, TAF7 was also shown to be required for embryonic development, but was dispensable for T cell differentiation [31]. Thus, it seems that differential *in vivo* TAF requirements may exist based on the cellular context and developmental stage of the cell.

In *Drosophila melanogaster*, two different GCN5-containing complexes have been reported: dATAC and dSAGA. dATAC acetylates H4K5 and H4K12 and dSAGA acetylates H3K9 and H3K14 [32–34]. These HAT complexes of Drosophila and vertebrates share GCN5, ADA3, SGF29 and ADA2 (ADA2a in dATAC, or ADA2b in dSAGA) proteins [22]. Loss-of-function mutations of dATAC complex subunits caused a sharp rise in deaths at the L3/pre-pupa transition [35], indicating that dATAC is indispensable for transcriptional activation of ecdysone-induced genes [35].

Ecdysone is synthetized in the prothoracic gland and acts as a master controller of *Drosophila melanogaster* metamorphosis. Ecdysone biosynthesis is regulated by proteins encoded by the *Halloween* gene family: *phantom/Cyp306A1* (*phm*), *shadow/Cyp315A1* (*sad*), *spook/Cyp307A1* (*spo*), *spookier/Cyp307A2* (*spok*), *disembodied/Cyp302A1* (*dib*), and *shade/Cyp314A1* (*shd*) [36, 37]. At target tissues, ecdysone binds to its receptor and activates specific transcriptional reprogramming at specific developmental stages [38, 39]. The broad effects of ecdysone and steroid hormone signaling absolve the investigation of regulatory mechanisms that can organize complex transcriptional events resulting from changes in steroid levels during development.

Here, we show that dTAF10+dTAF10b-containing dTFIID/dSAGA and dADA2a-containing dATAC complexes have a synergetic role in the regulation of Drosophila morphogenesis. Our results support the model that both dATAC and dTFIID play important roles in the activation of ecdysone biosynthetic *Halloween* genes. d*Taf10* mutations caused failures in morphogenesis at the late larval/early pupal stage, which suggests that dTAF10-containing complexes are necessary only for specific developmental stages. These observations support the idea that functionally different TAF-containing dTFIID complexes participate in gene regulation.

## Materials and Methods

### Fly stocks

Fly stocks were maintained at room temperature in yeast-cornmeal-glucose medium. *Taf10* ^*25*^ mutations were generated by imprecise excision of P(SUPor-P) Taf10b^KG01574^, which is located on Taf10-coding sequences. Other fly lines were obtained from the Bloomington Drosophila stock centre and the NIG-Fly was obtained from Japan or the Vienna RNAi center (VDRC). Animals used for microarrays, immunostaining for histone and dTFIID, and in ecdysone-feeding experiments were *w* ^*1118*^ and *Taf10* ^*25*^. These fly lines were harvested at spiracle eversion at late L3 larval stage. dTFIID siRNA silencing lines were *Taf5* ^*RNAi*^, *Taf8* ^*RNAi*^, *and Taf10* ^*RNAi*^.

Taf5^RNAi^ http://stockcenter.vdrc.at/control/product/~VIEW_INDEX=0/~VIEW_SIZE=100/~product_id=45955

Taf8^RNAi^ http://stockcenter.vdrc.at/control/product/~VIEW_INDEX=0/~VIEW_SIZE=100/~product_id=27870

Taf10^RNAi^ http://www.shigen.nig.ac.jp/fly/nigfly/rnaiDetailAction.do?input=sr&stockId=2859R-5

### DNA microarrays

Microarray analyses of Drosophila larvae were performed as described previously [40]. Microarray hybridization and array scanning were performed at IGBMC Strasbourg. Data obtained from microarray experiments are available at the European Bioinformatics Institute (http://www.ebi.ac.uk/arrayexpress/) under the accession numbers E-MTAB-3842 and E-MEXP-2125

### RT-PCR

For the measurement of the transcriptional activity of *Halloween* genes *w* ^*1118*^ and *Taf10* ^*25*^, L3 larvae were synchronized and collected at the spiracle eversion stage. Total RNA was extracted using a Qiagen RNeasy Mini Kit according to the manufacturer's instructions. cDNA template was generated from 1 μg RNA using a First Strand cDNA Synthesis Kit (ABI). Primer sequences used to detect transcript levels of *Halloween* genes have been previously described [35].

### Western blot

For protein analysis, flies were homogenized in Laemmli sample buffer using a pestle. Samples were separated on 10–15% gels using SDS-PAGE and transferred to Hybond-ECL (Amersham) nitrocellulose membranes. Membranes were incubated overnight with primary antibodies against histone H3 (Abcam ab1791) and K14-acetylated H3 (Upstate #07-353). For protein detection, IgG-HRP (DAKO) secondary antibodies were used and membranes were developed using a Millipore ECL kit.

### Immunostaining

For salivary gland polytene chromosome immunostaining of late L3 larvae, samples were incubated in PBS buffer with 3.7% paraformaldehyde and 45% acetic acid. Non-specific binding sites were blocked for 1 h at 25°C in 5% fetal calf serum (Lonza) or 5% BSA (Sigma-Aldrich) containing PBST (PBS + 0.1% Tween20). Then slides were incubated overnight at 4°C with anti-histone H3K9ac (Abcam ab4441, 1:200), anti-histone H3K14ac (Upstate 07-353, 1:200), anti-histone H4K12ac (Abcam ab1761, 1:200), anti-histone H4K8ac (Abcam ab1760, 1:100) anti-dADA2b (1:100) and anti-RNAPII (1BP-7G5, 1:500) [21, 34] antibodies. Polyclonal rabbit antibodies against dTAF10 and dTAF10b were generated at IGBMC [41]. dTAF1, dTAF5 and dTBP antibodies were a kind gift from Y. Nakatani. Samples were incubated for 1 h at 25°C with secondary antibodies (Dylight 549, Alexa Fluor 555, and Alexa Fluor 488 IgGs, Molecular Probes). Slides were covered with Prolong Gold and examined with an Olympus BX51 microscope attached to a DP70 camera.

### Ecdysone and cholesterol feeding

For ectopic addition of ecdysone and cholesterol treatment, second instar larvae were collected at the L2-L3 molting stage and then incubated for 24 h until they reached the middle L3 stage. 10 larvae were transferred to new ecdysone or cholesterol-containing medium. Ecdysone and cholesterol were diluted to final concentrations of 0.5 mM and 0.14 mg/g, respectively. During the experiment, vials containing 10 larvae each were kept at 25°C.

### Immunoprecipitations

Drosophila *w* ^*1118*^ and *Taf10* ^*25*^ L3 larvae were harvested and crushed using a pestle containing non-denaturing lysis buffer (1% Triton X-100, 50 mM Tris-HCl pH 8 and 150 mM NaCl) containing 1x Protease Inhibitor Cocktail on ice for 1 h. After lysis, cuticles were pelleted by centrifugation (400 g, 5 min, 4°C) and discarded. Protein concentrations of soluble fractions were measured using Bradford reagent. Lysates (300 μg) were precleared for 2 h using blocked Protein A-Sepharose beads (Sigma-Aldrich). Polyclonal anti-dTAF5 antibody was used for the immunoprecipitation. Lysates and antibody were incubated overnight at 4°C with Protein A-Sepharose beads (Sigma-Aldrich), then TAF5 complex-bound beads were collected by centrifugation (400 g, 1 min, 4°C) and washed four times with non-denaturing lysis buffer supplemented with 1x Protease Inhibitor Cocktail. For elution, the beads were boiled in 2x SDS loading buffer for 5 min and centrifuged at 2000 g for 5 min at 4°C. For Western blot analysis, both anti-dTAF5 and anti-dTAF4 polyclonal antibodies were diluted to 1:250.

## Results

### dTAF10 and dTAF10b are required for the L3 larval-pupal transition of morphogenesis

**Fig 1:**
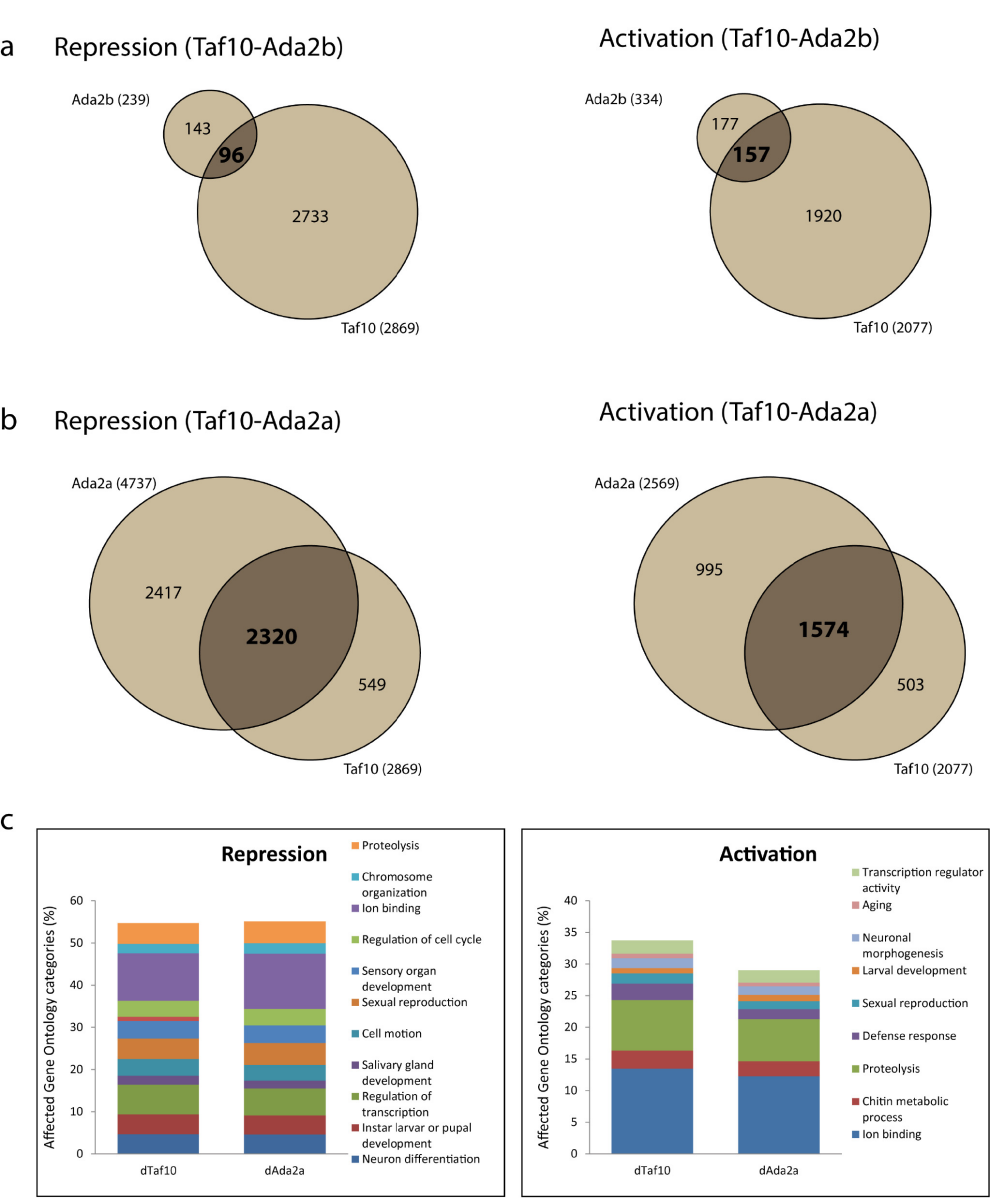
Steady state mRNA level changes in d*Taf10+*d*Taf10b* and d*Ada2a* (dATAC) mutants. (a.) Venn diagram showing repressed and activated genes in d*Taf10-* and d*Ada2b*-deficient larva. (b.) Venn diagram showing repressed and activated genes in d*Taf10*- and d*Ada2a*-deficient larva. (c.) Gene Ontology categories of genes affected by d*Taf10* and d*Ada2a* mutation with altered expression patterns compared to control.

In *Drosophila melanogaster*, dTAF10 proteins are present in two related complexes: the dTFIID general transcription factor complex and the dSAGA histone acetyltransferase complex [21, 22, 33]. To investigate the function of dTAF10s, we generated a Drosophila strain where the coding genes of two dTAF10 paralogues (d*Taf10* and d*Taf10b*) were deleted (S1 Fig). This strain hereafter is referred to as *Taf10* ^*25*^. In order to test whether loss of dTAF10 and dTAF10b resulted in transcriptional changes associated with dTFIID and/or dSAGA function, we performed a whole genome *Drosophila melanogaster* transcriptome analysis using an Affymetrix Drosophila2 microarray. To test whether dTAF10 and dTAF10b ablation affected genes controlled by dSAGA, we compared changes in steady state mRNA levels obtained after dTAF10+dTAF10b ablation and alterations in mRNA levels were detected in a strain from which dADA2b, a dSAGA subunit was depleted (Fig 1A) [40]. We synchronized *Taf10* ^*25*^ mutant larvae (lacking both d*Taf10* and d*Taf10b)* to spiracle eversion before puparium formation. At this stage of larval development, the molting hormone ecdysone induces apoptosis of polytene tissues and the formation of diploid adult tissues can be observed. Due to this developmental reprogramming, the majority of the genome undergoes expressional changes [42, 43].

When we eliminated both dTAF10 and dTAF10b, we found altered expression in one third of the transcribed genome (2869 genes were repressed and 2077 genes were activated out of 14 500 genes present on the microarray) (Fig 1A and 1B). Surprisingly, the sets of genes with altered mRNA expression in d*Taf10+*d*Taf10b* mutant overlapped only partially with the steady state mRNA expression changes observed in dADA2b mutants (Fig 1A and S2 Fig) [40]. The fact that dTAF10+dTAF10b ablation did not affect the same set of mRNAs as dADA2b depletion, suggested that the mRNA level changes observed upon the ablation of dTAF10+dTAF10b corresponded to dTFIID function rather than dSAGA function (Fig 1A and 1B).

Interestingly, the changes in steady state mRNA levels observed in d*Taf10+*d*Taf10b* mutants were comparable to changes previously reported in *dAda2a* (a subunit of the dATAC HAT complex) mutants [35] (Fig 1A and S2 Fig). We performed a Gene Ontology (GO) analysis on the sets of genes affected in both d*Taf10+*d*Taf10b* and d*Ada2a* mutants and found that in both mutants the affected gene categories with the highest significance score were mostly developmental regulatory genes. In the two compared samples the numbers of genes in the highest ranked GO terms showed high similarity on repressed and also on activated genes (Fig 1C and S1 Table). These results suggest co-operation between dTAF10-containing dTFIID and dATAC complexes in the regulation of steady state mRNA levels.

### dTAF10 and dTAF10b are required for dSAGA-specific H3K14 acetylation but not for dATAC-dependent H4K12 acetylation

**Fig 2:**
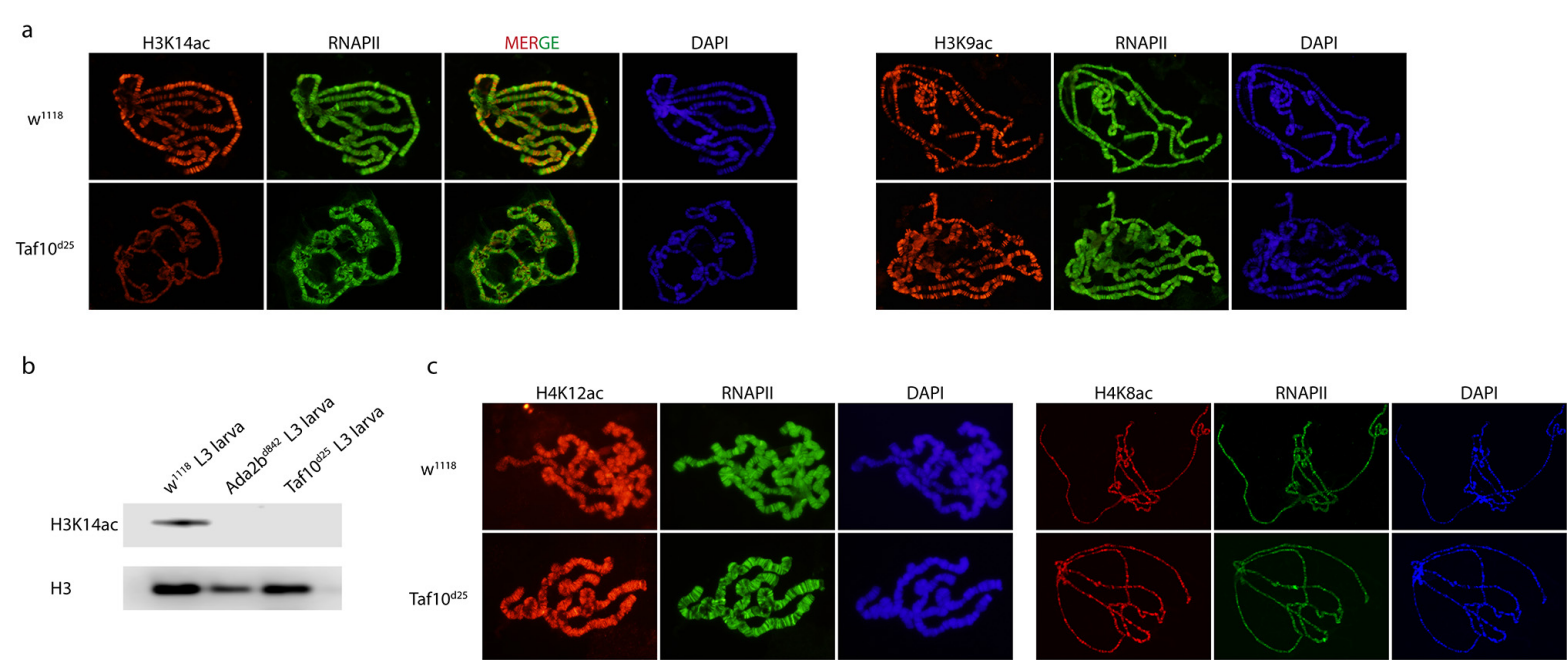
Histone acetylation changes regulated by dTAF10+dTAF10b-containing complexes. (a.) Immunostaining of polytene chromosomes in late third instar *Taf10* ^25^ and control (*w* ^*1118*^) larvae showing dSAGA-specific H3K14ac and H3K9ac. General RNAPII staining is also shown as a control. (b.) Western blot of H3K14ac levels in *Taf10* ^25^ mutants and *w* ^*1118*^ control animals. H3 detected as a loading control. (c.) Immunostaining of polytene chromosomes in late third instar *Taf10* ^25^ and control (*w* ^*1118*^) larvae with dATAC-specific H4K12ac and H4K8ac antibodies. General RNAPII staining is also shown as a control.

In order to test whether the ablation of dTAF10 and dTAF10b could influence HAT activity of dSAGA and dATAC complexes, we investigated global histone acetylation levels in d*Taf10+*d*Taf10b* mutant larvae. In particular, we studied the histone H3K9ac and H3K14ac levels, which are dSAGA-dependent, and histone H4K8ac and H4K12ac levels, which have been reported to be dependent on dATAC [32–34]. In *Taf10* ^*25*^ mutant larvae the level of dSAGA-specific H3K14ac was reduced, while dATAC-specific H4K8ac and H4K12ac levels were comparable to the controls (Fig 2A, 2B, 2C and S3 Fig). However, we did not observe a difference between the H3K9ac levels in T*af10* ^*25*^ mutant larvae and control samples. This result is consistent with the observation that dTAF10b is also a stable subunit of the dSAGA complex. On the other hand, the observation that dATAC-specific acetylation of H4K8 and H4K12 was not altered in d*Taf10+*d*Taf10b* mutants is in accordance with the fact that neither dTAF10 nor dTAF10b is a subunit of the dATAC complex. Thus, the changes in steady state mRNA levels resulting from the lack of dTAF10 and dTAF10b can be explained by the cooperation between dATAC and dTFIID (or dSAGA) complexes.

### Genome-wide binding of dTAF5 to polytene chromosomes is reduced in the absence of dTAF10 and dTAF10b

**Fig 3:**
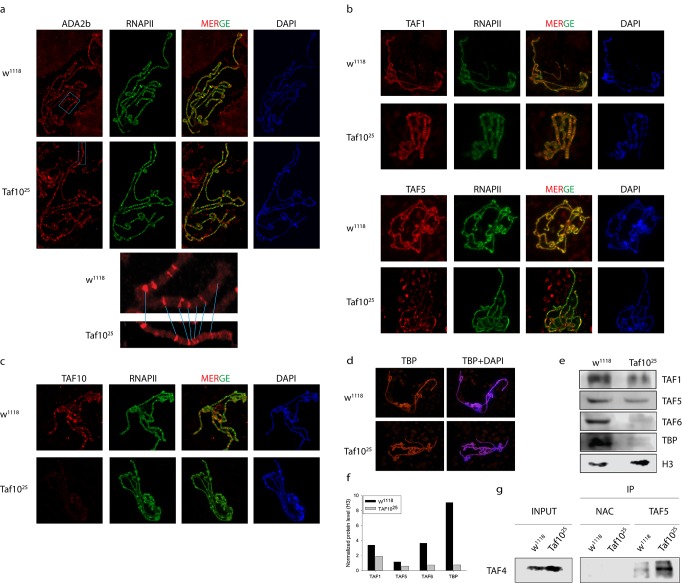
dTFIID subunit localization in d*Taf10+*d*Taf10b* mutants. (a.) Immunostaining of polytene chromosomes in late third instar *Taf10* ^25^ and control (*w* ^*1118*^) larvae with dADA2b-specific antibodies. (b.) Immunostaining of polytene chromosomes in late third instar *Taf10* ^25^ and control (*w* ^*1118*^) larvae with TAF1- and TAF5-specific antibodies. (c.) Immunostaining of polytene chromosomes in late third instar *Taf10* ^25^ and control (*w* ^*1118*^) larvae with TAF10-specific antibodies. (d.) Immunostaining of polytene chromosomes in late third instar *Taf10* ^25^ and control (*w* ^*1118*^) larvae with TAF10-specific or TBP-specific antibodies. On panels a,b,c,d general RNAPII staining is also shown as a control. (e.) Western blot detection of histone H3 and TAF1, TAF5, TAF6, and TBP subunits of TFIID from late third instar *Taf10* ^25^ and control (*w* ^*1118*^) animals. (f.) Western blot signals were quantified and normalized to H3. (g.) Western blot detection of TAF4 protein in anti-TAF5 immunoprecipitated samples from control (*w* ^*1118*^) and *Taf10* ^*25*^ mutant larva. Inputs for the demonstration of equal loading and the NPC are shown. Abbreviations: NPC = no protein control.

Next, we tested whether the ablation of dTAF10 isoforms would disturb the binding of dSAGA or dTFIID complex subunits. To test this, we performed immunostaining on Drosophila salivary gland polytene tissues both in control and *Taf10* ^*25*^ mutant animals and observed the localization of dSAGA and dTFIID subunits (Fig 3A, 3B, S4A and S4B Fig). Interestingly, the localization of the dSAGA subunit dADA2b did not change in *Taf10* ^*25*^ mutants, suggesting that dSAGA function was not altered in the absence of TAF10 (Fig 3A and S4A Fig).

Additionally, dTAF1, the largest subunit of dTFIID and TBP also showed unchanged localization in control and *Taf10* ^*25*^ mutants. On the other hand, the localization of dTAF5 was markedly reduced in the *Taf10* ^*25*^mutant (Fig 3B, panel 4 merge). In accordance, dTAF10 was only found at specific sites of wild-type polytene chromosomes compared to dTAF1 or dTAF5 (Fig 3C, lane 1 compared to Fig 3B, lanes 1 and 3).

While dTAF1 or dTAF5 co-localized with RNAPII, in the case of dTAF10 we could detect only minor overlap with RNAPII (Fig 3C). In order to verify whether reduced co-localization of dTAF5 in *Taf10* ^*25*^ mutant flies was due to dTFIID degradation, we measured protein levels of several dTFIID-specific subunits in *Taf10* ^*25*^ mutants by Western blot analysis (Fig 3E). In the absence or presence of dTAF10+dTAF10b detected identical levels of dTAF1 and dTAF5 were detected, which suggested that the weaker dTAF5 co-localization to polytene chromosomes was not the consequence of reduced dTAF5 protein levels (Fig 3E and S5 Fig). On the contrary, significantly lower levels of dTAF6 and dTBP were detected in *Taf10* ^*25*^ mutants compared to controls. However, the polytene-co-localized dTBP was not affected (Fig 3D and 3E) suggesting that the reduced level of dTBP was still sufficient for binding to specific target sequences. Taken together, these results suggest that loss of dTAF10 isoforms did not lead to dTAF5 destabilization. However, the deletion of d*Taf10* genes may result in the disappearance of the dTFIID 7TAF modules from polytene chromosomes. This conclusion is in agreement with findings that in human cells, both TAF10 and TAF5 are required for the integrity of 7TAF subcomplex [11, 44]. In contrary, dTAF1- and dTBP-containing submodules of dTFIID seem to be able to bind to polytene chromosomes even in the absence of dTAF10 isoforms.

### Loss of dTAF10+dTAF10b does not affect dTAF4 and dTAF5 interaction

Earlier reports suggested that functional TFIID complexes without TAF10 might exist depending on the developmental stage or the cellular conditions of the cell [12, 27, 30]. In order to evaluate whether dTAF10 proteins are required for the dTFIID complex formation in *Drosophila melanogaster* we performed immunoprecipitation experiments to test the incorporation of dTAF4 into dTFIID complexes in wild-type and d*Taf10*+d*Taf10b* mutants. As expected, dTAF4 was immunoprecipitated by anti-dTAF5 antibody from wild-type extracts (Fig 3G). Surprisingly, anti-dTAF5 antibody was able to co-immunoprecipitate dTAF4 in *Taf10* ^*25*^ mutant larvae; suggesting that partial dTAF4-dTAF5-containing dTFIID subcomplexes could be formed in the absence of dTAF10 (Fig 3G).

### d*Taf10* mutation results in failure of ecdysone-induced metamorphosis through the regulation of ecdysone biosynthetic gene expression

**Fig 4:**
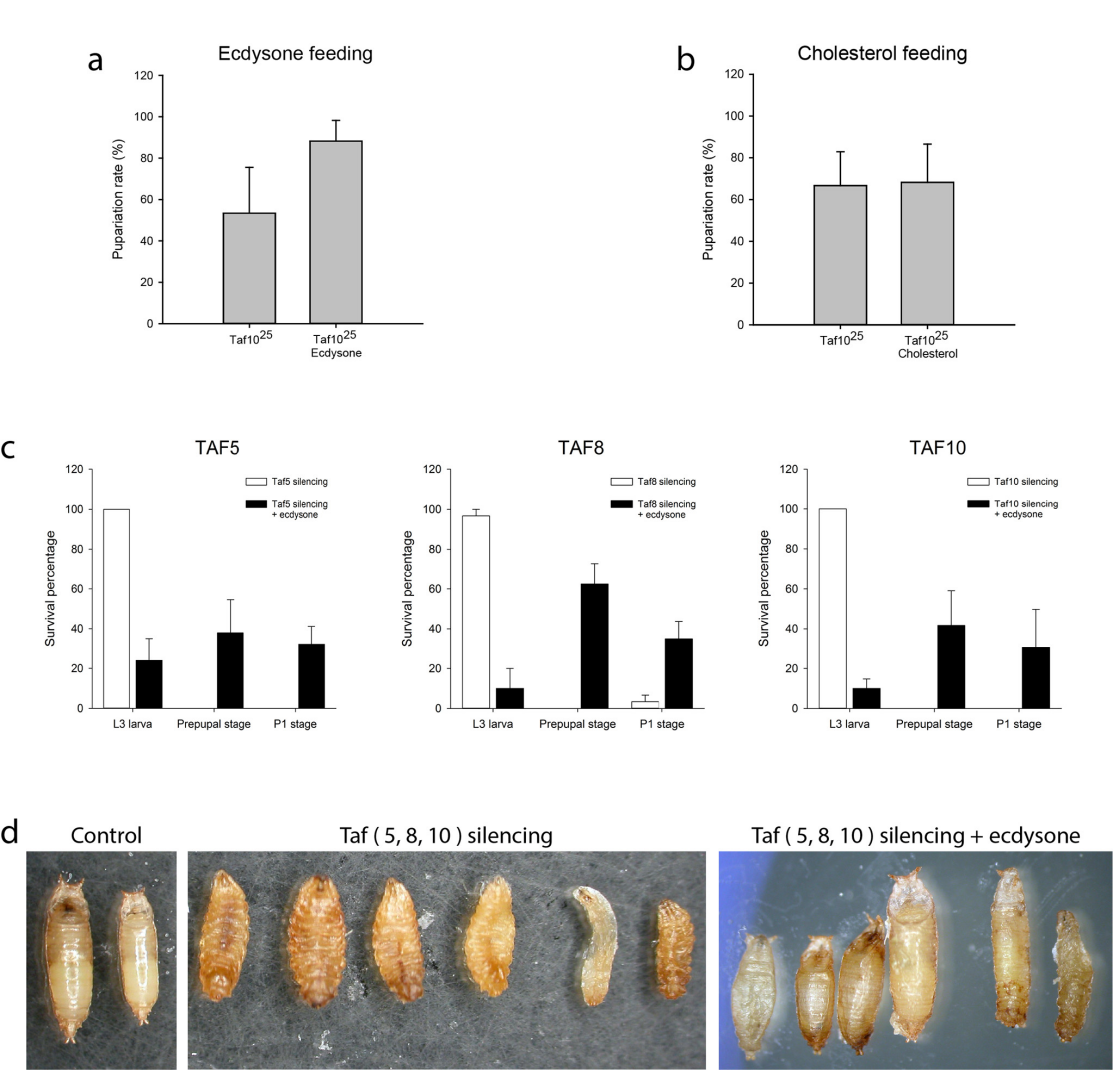
Ecdysone extends the life span of *Taf10* ^*25*^ null and Taf5-, Taf8- and Taf10-silenced animals. (a.) *Taf10* ^*25*^ homozygous larvae exhibit an extended lethal phase when their diet is supplemented with ecdysone at mid L3 stage. Mean values and standard errors were calculated from 3 independent experiments containing 30 larvae in each category. (b.) The pupariation rate of *Taf10* ^*25*^ and control larvae fed on cholesterol-containing medium in middle L3 stage. Mean values and errors were calculated from 3 independent experiments containing 30 larvae in each category. (c.) Animals in which *Taf5*, *Taf8* and *Taf10* were siRNA silenced exclusively at the ring gland (open bars) died at the third instar larval stage. Animals fed on ecdysone-containing food in mid-L3 stage could initiate pupariation (black bars). Means and standard errors were calculated from 30 larvae in each genotype.(d.) Result of siRNA silencing of *Taf5*, *Taf8* and *Taf10* at the ring gland. Images of the animals that are unable to execute puparium formation. RNAi larvae are often larger than control and show morphological abnormalities, such as improper spiracle eversion or altered body shape.

The loss of dTAF10 proteins results in failure of transcriptional reprogramming and in accordance with this, we found that the majority of d*Taf10* mutants were unable to form normal pupa. Approximately 50% of animals were arrested in the L3 larval stage, which was also delayed in the mutants (Fig 4A). The remaining 50% formed aberrant pupae. Earlier experiments showed that loss of dATAC histone acetyltransferase complex caused similar developmental failures [35]. Since the developmental failures in dATAC mutants resulted from the absence of the molting hormone ecdysone [35], we wondered whether ecdysone feeding could rescue puparium formation failure caused by *Taf10* ^*25*^ deletion. Ecdysone addition to the medium improved the development of *Taf10* ^*25*^ mutant larvae as indicated by an increased number of larvae that initiated pupariation (Fig 4A). Ecdysone is synthesized from cholesterol in the ring gland and its synthesis is activated by prothoracicotropic hormone (PTTH). Thus, the lack of activation of *Halloween* genes could be the result of a missing signal molecule or metabolic precursor. First, we addressed whether a failure in cholesterol uptake resulted in defects in pupariation. To this end, we fed *Taf10* ^*25*^ mutants with cholesterol to determine whether defects could be rescued. Surprisingly, cholesterol was unable to rescue defects (Fig 4B), suggesting that it was not the lack of precursor that caused the failure of hormone production. The number of polytene cells in the ring gland and morphology of the gland, however, were comparable to that of *Taf10* ^*25*^ and wild-type (data not shown). Microarray data also suggested that the ring gland of mutants was normal: we found that transcripts of calmodulin and PTTH genes were exclusively present in this tissue (data not shown). Thus, we concluded that the failures in pupariation of d*TAF10+*d*TAF10b* mutants were not resulted in defective cholesterol transport or ring gland morphology. These results suggest that dTAF10 proteins are required for ecdysone-induced larval-pupal transition.

Next, we investigated whether gene expression changes and the morphogenesis delay caused by the lack of dTAF10 were due to the lack of ecdysone hormone. Since ecdysone is synthesized in the prothoracic gland, we eliminated d*Taf10* messengers only in the prothoracic gland cells by producing siRNA against d*Taf10* with the help of a *phantom*-GAL4 driver. Indeed, the absence of dTAF10 in the prothoracic gland resulted in developmental failures in the late L3 larva stage (Fig 4C). Ecdysone feeding restored ability of larvae to form puparium. These results clearly indicated that the gene expression changes and failure in morphogenesis of dTAF10 mutant animals were due to the lack of the molting hormone ecdysone (Fig 4C). To further test this, we expressed siRNA constructs against d*Taf5* and d*Taf8*, two subunits of the 7TAF submodule of the dTFIID complex in the prothoracic gland. As we expected, the targeted silencing of d*Taf5* and d*Taf8* resulted in similar loss of molting phenotype, as was detected in d*Taf10*-silenced animals (Fig 4C and 4D). d*Taf5-*, d*Taf8-* and d*Taf10-*silenced animals could reach the early pupal stages by ecdysone feeding (Fig 4C and 4D). Together, these results suggest that dTFIID has an essential regulatory function in early stages of ecdysone hormone biosynthesis in prothoracic cells. Furthermore, larval-pupal morphogenesis and transcriptional reprogramming cannot be initiated in the absence of dTFIID function.

**Fig 5:**
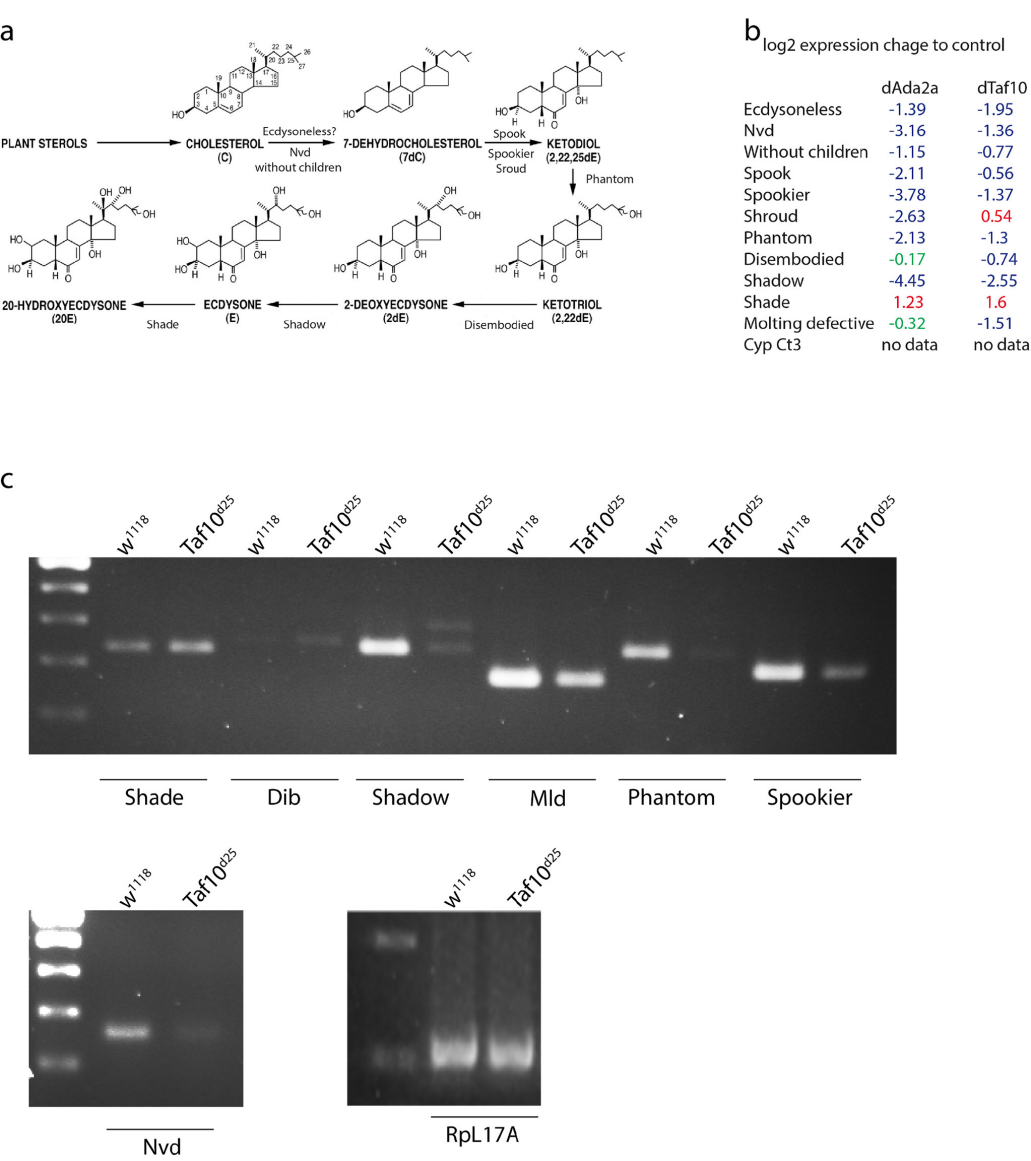
Ecdysone biosynthetic *Halloween* gene expression in *Taf10* ^*25*^ mutants. (a.) Involvement of cytochrome P450 genes in the synthesis of the steroid hormone ecdysone. Figure adapted from Warren 2006. (b.) List of genes implicated in ecdysone biosynthesis that show altered expression in d*Taf10* ^*25*^ and d*Ada2a* mutants compared to control animals as determined by the microarray experiment. Repressed genes are shown as blue, activated genes are shown in red and genes with no change are shown in green. (c.) mRNA levels of *Halloween* gene were determined by RT-PCR and corresponding bands are shown in *Taf10* ^*25*^ mutant and control (*w* ^*1118*^).

Previously, we reported that the lack of dATAC subunits resulted in decreased expression of the ecdysone biosynthetic P450 genes [35]. The similarities between the changes of dTAF10-dependent and dAda2a-(dATAC)-dependent transcriptomes prompted us to test whether dTAF10 proteins were also necessary for the expression of the P450 cytochrome genes. Microarray results showed that cytochrome P450 genes, which encode proteins involved in ecdysone biosynthesis in the ring gland, were down regulated in *Taf10* ^*25*^ *-*null mutants (Fig 5B). In contrast, *shade*, which converts ecdysone to its active form 20-OH ecdysone in the midgut, was upregulated. This suggests that dTAF10 proteins affect the expression of ecdysone biosynthetic genes only in the ring gland (Fig 5A). In agreement with this, RT-PCR analysis showed a strong reduction in the mRNA levels of *shadow*, *molting defective*, *phantom*, *spookier*, and *neverland* in d*Taf10* ^*25*^ mutants (Fig 5C). In contrast, the mRNA level of *shade* was increased in d*Taf10* ^*25*^ mutants showing a functional loss in the regulation of molting. The observation that mutations in ecdysone biosynthetic genes resulted in failure in developmental gene reprogramming and that d*Taf10* ^*25*^ mutants could be rescued by ecdysone feeding (Figs 4 and 5) suggested that the robust expressional change in d*Taf10* ^*25*^ mutants was likely due to the loss of ecdysone hormone. In addition, similar phenotypes were detected by the lack of dTAF10/dTAF10b and dADA2a, indicating potential co-operation between dTAF10-containing complexes and dATAC complexes in the regulation of ecdysone-driven morphogenesis.

## Discussion

### The role of dTAF10-containing dTFIID in Drosophila development

The multisubunit TFIID complex has an important role in the coordination of transcription initiation. The formation of TFIID is nucleated by the assembly of the 7TAF submodule composed of two copies of TAF4-TAF12, TAF6-TAF9 histone fold pairs and TAF5, and one copy of the TAF8-TAF10 histone fold pair [11, 44]. TAF proteins including TAF10 are also subunits of dSAGA, another HAT complex involved in transcription. Earlier studies highlighted the importance of TAF10 in the assembly and function of TFIID [27–30]. However, less is known about the structural and functional role of TAF10 in SAGA. In Drosophila, two dTAF10 isoforms have been reported, dTAF10 and dTAF10b [21]. Later it was shown that dTAF10 is a subunit of dTFIID, while dTAF10b is a subunit of dSAGA [23]. The two *Taf10* genes of Drosophila are located very close to each other and overlap in their assumed regulatory regions. In this study, we knocked out both genes to study the role of dTAF10 in *Drosophila melanogaster*. Here we report that neither of the two dTAF10 proteins have essential functions in transcriptional regulation at early larval stages. Animals lacking both d*Taf10* genes reached L3 larval stage but failed to form puparium and adult tissues. A possible explanation for this could be that maternal dTAF10 and/or dTAF10b is sufficient to bypass early developmental stages and in the loss of zygotic *Taf10* and *Taf10b* expression. Animals died only at the larval-pupal transition when the molting hormone ecdysone was crucial for further development. When d*Taf10* was eliminated using siRNA, resulting in the disappearance of both maternal and zygotic d*Taf10* mRNA, animals could reach the same developmental stage as the *Taf10* ^*25*^ null mutants. The fact, that d*Taf10* silencing resulted in an 80% reduction in specific mRNA level in all larval stages, led us to conclude that dTAF10 was essential only for larval-pupal morphogenesis (S6 Fig). Additionally, this finding suggests that dTAF10 is not always an essential part of the dTFIID. In other words, functional dTFIID could be formed even in the absence of dTAF10. In addition, the siRNA silencing of *dTaf8*, the histone fold partner of dTAF10, in dTFIID caused the same late larval-early pupal lethality as was observed in *Taf10* ^*25*^ mutants. In contrary, the elimination of another dTFIID subunit, dTAF5, resulted in lethality at the L2-L3 larval stage. According to our results and in good agreement with results from Wright et al. [8], a dTFIID sub-complex, which does not contain the dTAF8-dTAF10 heterodimer as a stable component, may exist during Drosophila metamorphosis. In accordance with this observation, Jacq et al. [12] reported the biochemical isolation of both TAF10-lacking and TAF10-containing TFIID complexes from human HeLa cells [12]. Our study reveals an additional dTFIID function and supports a model in which the dTAF8-dTAF10 histone fold pair is not necessary for all developmental stages. This finding is also in agreement with observations from TAF10-lacking keratinocytes of adult epidermis [30]. However, the role of partial dTAF10b-lacking dSAGA complexes in the observed phenotypes cannot be ruled out.

### d*Taf10+*d*Taf10b* mutation influences dTFIID complex formation

As we report here, in the absence of dTAF10, animals cannot form normal pupa and subsequently die because they cannot initiate transcriptional reprogramming which is regulated by the molting hormone ecdysone. During larval-pupal morphogenesis, a large ecdysone peak appears at late L3 larval stage 92 h after eggs hatch. The increase in ecdysone levels activates polytene tissue apoptosis and the production of diploid tissues from imaginal discs. During these steps, major transcriptional reprogramming results in the activation and repression of thousands of genes [42, 43]. Our data show that the dTAF10 proteins are essential for transcriptional reprogramming as in the absence of dTAF10 and dTAF10b on approximately one third of the Drosophila genes (5000 genes) transcription is altered, probably due to the inappropriate dTFIID or dSAGA function resulting from the lack of dTAF10s. The fact that the changes in gene expression following d*Taf10+*d*Taf10b* deletion did not overlap with changes resulting from the ablation of a dSAGA-specific subunit, dADA2b, suggests that the expression changes reflected the role of dTAF10 in dTFIID rather than the role of dTAF10b in dSAGA. In agreement with this, the absence of dTAF10, but not that of dTAF1 and dTBP, likely perturbed the recruitment of dTAF5 to polytene chromosomes. This suggests that a partial dTFIID subcomplex, presumably containing dTAF1 and dTBP, could be formed. Based on our immunoprecipitation results, we conclude that in the absence of dTAF10s, a partial dTAF4-dTAF5-containing dTFIID subcomplex is formed. In contrast, the loss of dTAF10 proteins caused a reduction in dTAF6 and dTBP protein levels, which affected the formation of other dTFIID subcomplexes. Finally, in the absence of dTAF10+dTAF10b we could not detect alterations in RNAPII localization to polytene chromosomes in *Taf10* ^*25*^ mutants compared to controls. Thus, it is possible that RNAPII transcription occurs in salivary gland cells in the absence of dTAF10 and/or dTAF10b. Knocking out TAF10 in mice caused embryonic lethality at 5.5 days of embryonic life. The ablation of TAF10 in mouse embryonic keratinocytes influenced the expression of many genes and the loss of TAF10 in epidermal cells did not affect the transcription and cellular stability of the examined genes [28, 30]. Similarly, TAF7 has been reported to be required for embryonic development, but not T-cell differentiation [31]. Based on these results, dTAF10-containing complexes may be necessary only at defined developmental stages and/or in tissues in order to regulate specific transcription programs. Moreover, it seems that TAF10 is required for embryonic transcription modes rather than later in highly differentiated cells. Our results support this idea since the exclusive silencing of d*Taf10+*d*Taf10b* in prothoracotropic tissues resulted in the same L3 larva-early pupal lethality as observed in *Taf10* ^*25*^ *-*null mutant flies. The fact that the loss of dTAF10 proteins in the ring gland could be rescued by ectopic ecdysone addition confirmed that dTAF10- and/or dTAF10b-containing complexes, probably dTFIID, had an exclusive role in the production of ecdysone. We identified several cytochrome P450 genes (*shadow*, *molting defective*, *phantom*, *spookier*, *neverland*), which were down-regulated in the absence of dTAF10s. This suggests that dTAF10- and/or dTAF10b-containing complexes had an essential role in the transcription of ecdysone biosynthetic genes. We believe that the gene expression changes detected in *Taf10* ^*25*^-null mutants were a consequence of perturbed ecdysone production.

### Synergistic role of dTAF10/dTAF10b-containing complexes and the dATAC HAT complex

The *Taf10* ^*25*^ mutation affected steady state mRNA levels of the same sets of genes as mutation of the HAT dATAC complex subunit d*Ada2a*. Thus, dTAF10/dTAF10b-containing complexes and dATAC had a role in the activation of ecdysone biosynthetic *Halloween* genes at specific developmental stages. Since dTAF10 and dTAF10b are not part of the dATAC complex it is conceivable that dTAF10/dTAF10b-containing complexes (probably dTFIID) and the dATAC complexes have a synergistic role in the transcriptional activation of *Halloween* genes. It has been shown that the dATAC complex can directly interact with dTBP *in vivo* [45], therefore this two-step regulation may be important for the activation of ecdysone biosynthetic genes at specific developmental stages. Previously, it was reported that TAF1, the largest subunit of TFIID, has higher affinity to tetra-acetylated histone H4 substrates (acetylated H4K5, K8, K12 and K16), which contain TAF1 tandem bromodomains [46, 47]. These bromodomains may be required for the positioning of TFIID next to nucleosome-bound territories of the chromosome. Additionally, TATA sequences are usually located in close proximity to nucleosomes *in vivo* [48] and acetylation of H4K5, K8 and K12 has been correlated with transcriptional activation [49]. Based on these findings, the bromodomain containing TAF1 could be targeted to promoters by acetylated tails of histone H4 around the TSS. A simple hypothesis linking histone acetylation and core promoter recognition is that HATs such as dATAC are recruited prior to dTFIID binding. dATAC could facilitate the acetylation of K5 and K12 of histone H4 at the promoter region thereby increasing the binding affinity of dTFIID. We believe that dATAC creates an acetylation pattern on histone N-terminal tails that favours transcriptional activation at early steps of transcription. This connects histone acetylation and transcription initiation by enhancing the recruitment of dTFIID to genes marked with numerous acetylated histones.

## Supporting Information

S1 Fig The *Taf10* genomic region and mutant alleles. The TAF10 coding genes (*Taf10a* and *Taf10b*), their relative positions and extension of deletions generated by mobilization of P element are shown.
(TIF)

S2 Fig Scatter plot showing the expressional changes of entire gene set in the microarray analysis. The position of each dot on the scatter plot corresponds to the signal intensity change (log_2_ scale) of a single gene. The normalized log_2_ expression change of *Taf10* are shown on the x and d*Ada2a* or d*Ada2b* on y axes The middle line indicates the expressional change values that are similar in d*Taf10* mutants d*Ada2a* or d*Ada2b* respectively (similar levels of expression change in both mutants).
(TIF)

S3 Fig Histone acetylation changes regulated by dTAF10+dTAF10b-containing complexes. (a.) Immunostaining of polytene chromosomes in late third instar *Taf10* ^25^ and control (*w* ^*1118*^) larvae showing dSAGA-specific H3K14ac and H3K9ac. General RNAPII staining is also shown as a control. (b.) Immunostaining of polytene chromosomes in late third instar *Taf10* ^25^ and control (*w* ^*1118*^) larvae with dATAC-specific H4K12ac and H4K8ac antibodies. General RNAPII staining is also shown as a control.
(TIF)

S4 Fig dTFIID subunit localization in d*Taf10*+d*Taf10b* mutants. (a.) Immunostaining of polytene chromosomes in late third instar *Taf10* ^25^ and control (*w* ^*1118*^) larvae with dADA2b-specific antibodies. General RNAPII staining is also shown as a control. (b.) Immunostaining of polytene chromosomes in late third instar *Taf10* ^25^ and control (*w* ^*1118*^) larvae with TAF1- and TAF5-specific antibodies. General RNAPII staining is also shown as a control. (c.) Immunostaining of polytene chromosomes in late third instar *Taf10* ^25^ and control (*w* ^*1118*^) larvae with TAF10-specific antibodies. General RNAPII staining is also shown as a control. (d.) Immunostaining of polytene chromosomes in late third instar *Taf10* ^25^ and control (*w* ^*1118*^) larvae with TAF10-specific or TBP-specific antibodies. General RNAPII staining is also shown as a control.
(TIF)

S5 Fig The expressional changes of TAF protein encoding genes in *Taf10* ^*25*^ mutants compared to control animals as determined by the microarray experiment. (TIF)

S6 Fig The effect of *Taf10* silencing by siRNA on d*Taf10* mRNA level at different developmental stages. (TIF)

S1 Table Gene Ontology categories of genes affected by d*Taf10* and d*Ada2a* mutation with altered expression patterns compared to control. (PDF)
