# Supplementary figures and images for: dTAF10- and dTAF10b-Containing Complexes Are Required for Ecdysone-Driven Larval-Pupal Morphogenesis in Drosophila melanogaster
Source: PLoS One. 2015 Nov 10;10(11):e0142226. doi: 10.1371/journal.pone.0142226 (PMC4640578; doi:10.1371/journal.pone.0142226)

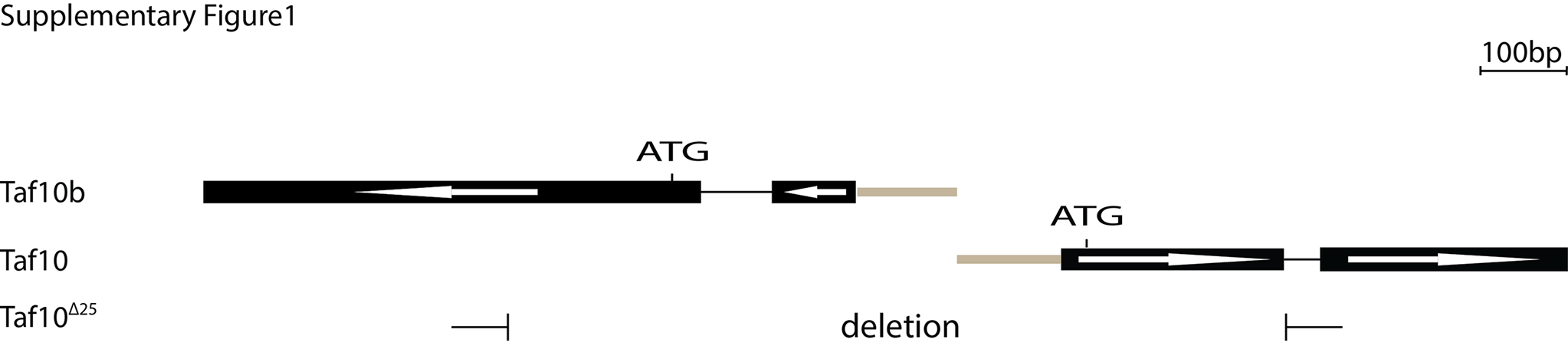

Supplement: S1 Fig — The TAF10 coding genes (Taf10a and Taf10b), their relative positions and extension of deletions generated by mobilization of P element are shown. (TIF) [file pone.0142226.s001.tif]

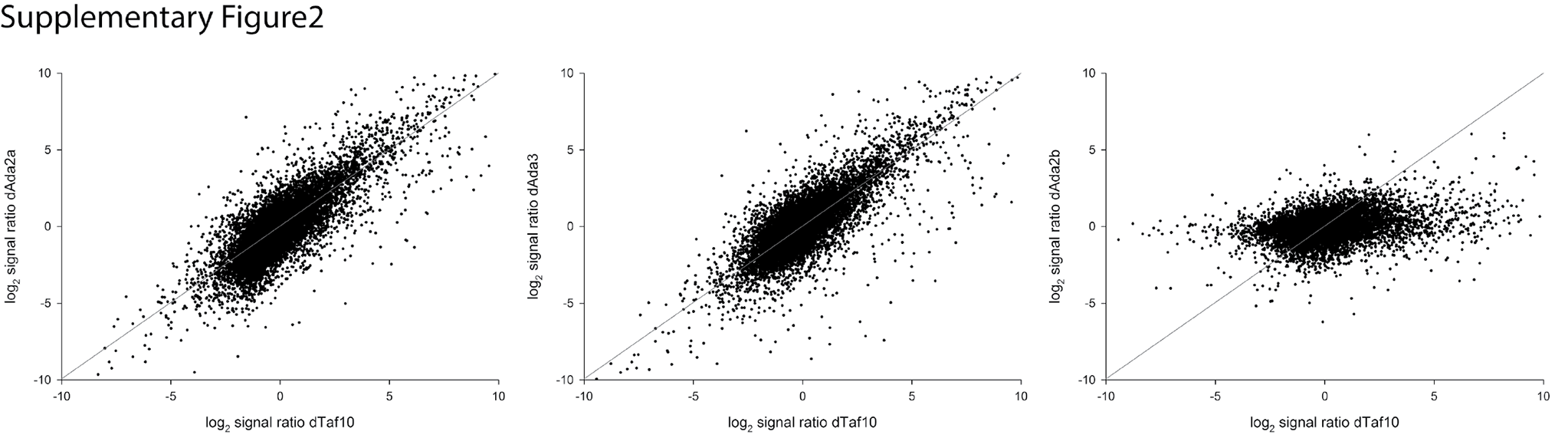

Supplement: S2 Fig — The position of each dot on the scatter plot corresponds to the signal intensity change (log2 scale) of a single gene. The normalized log2 expression change of Taf10 are shown on the x and dAda2a or dAda2b on y axes The middle line indicates the expressional change values that are similar in dTaf10 mutants dAda2a or dAda2b respectively (similar levels of expression change in both mutants). (TIF) [file pone.0142226.s002.tif]

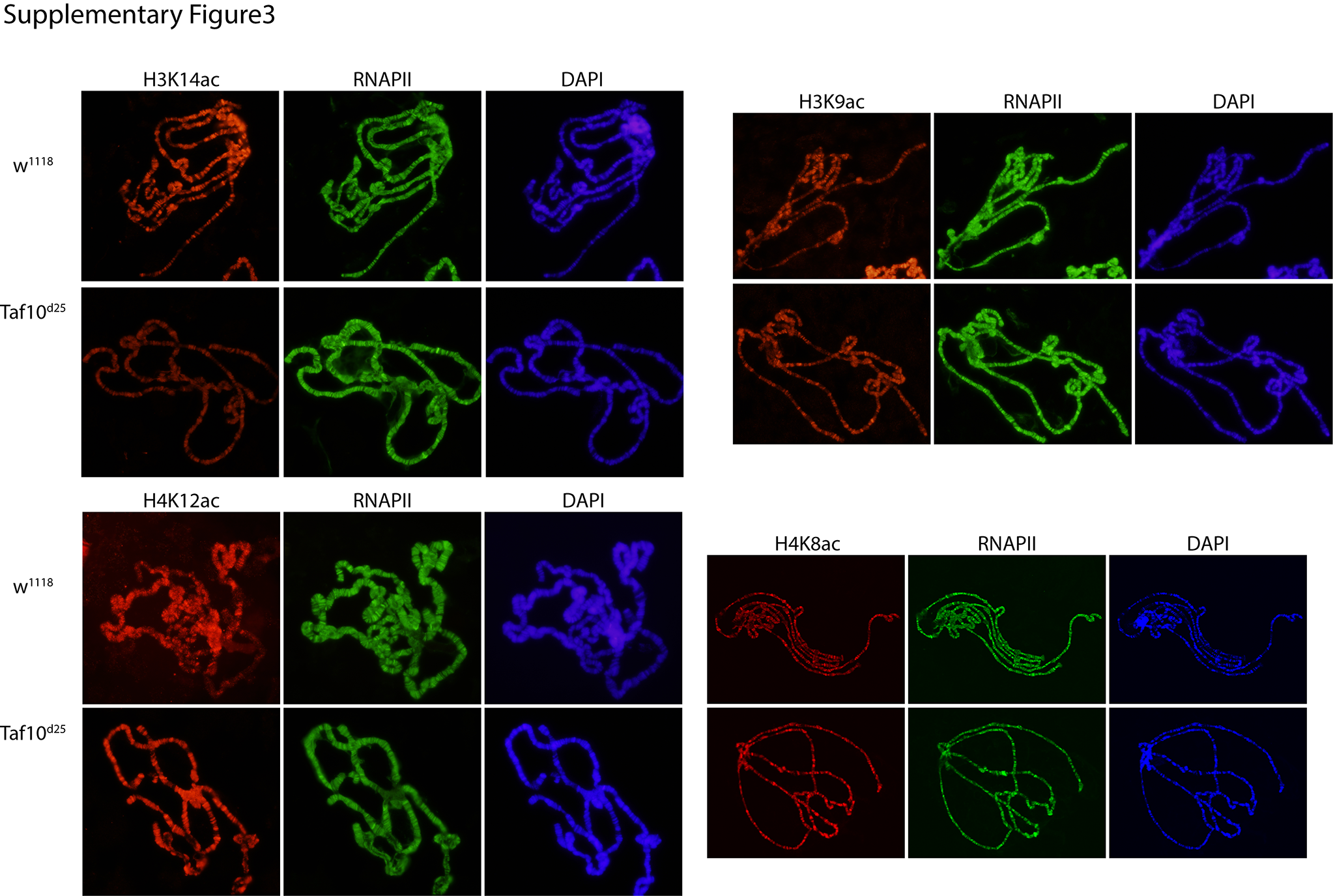

Supplement: S3 Fig — (a.) Immunostaining of polytene chromosomes in late third instar Taf10 25 and control (w 1118) larvae showing dSAGA-specific H3K14ac and H3K9ac. General RNAPII staining is also shown as a control. (b.) Immunostaining of polytene chromosomes in late third instar Taf10 25 and control (w 1118) larvae with dATAC-specific H4K12ac and H4K8ac antibodies. General RNAPII staining is also shown as a control. (TIF) [file pone.0142226.s003.tif]

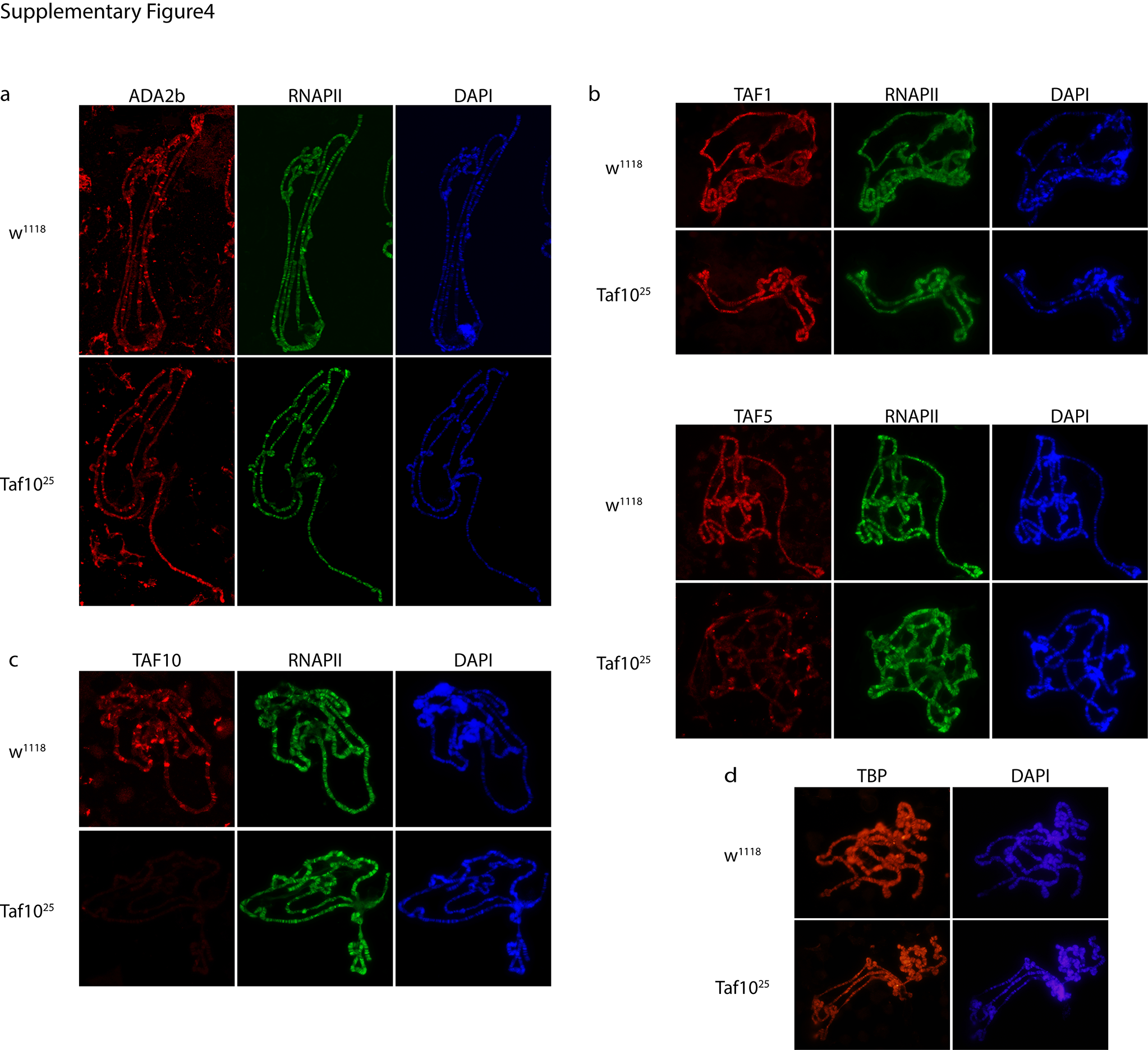

Supplement: S4 Fig — (a.) Immunostaining of polytene chromosomes in late third instar Taf10 25 and control (w 1118) larvae with dADA2b-specific antibodies. General RNAPII staining is also shown as a control. (b.) Immunostaining of polytene chromosomes in late third instar Taf10 25 and control (w 1118) larvae with TAF1- and TAF5-specific antibodies. General RNAPII staining is also shown as a control. (c.) Immunostaining of polytene chromosomes in late third instar Taf10 25 and control (w 1118) larvae with TAF10-specific antibodies. General RNAPII staining is also shown as a control. (d.) Immunostaining of polytene chromosomes in late third instar Taf10 25 and control (w 1118) larvae with TAF10-specific or TBP-specific antibodies. General RNAPII staining is also shown as a control. (TIF) [file pone.0142226.s004.tif]

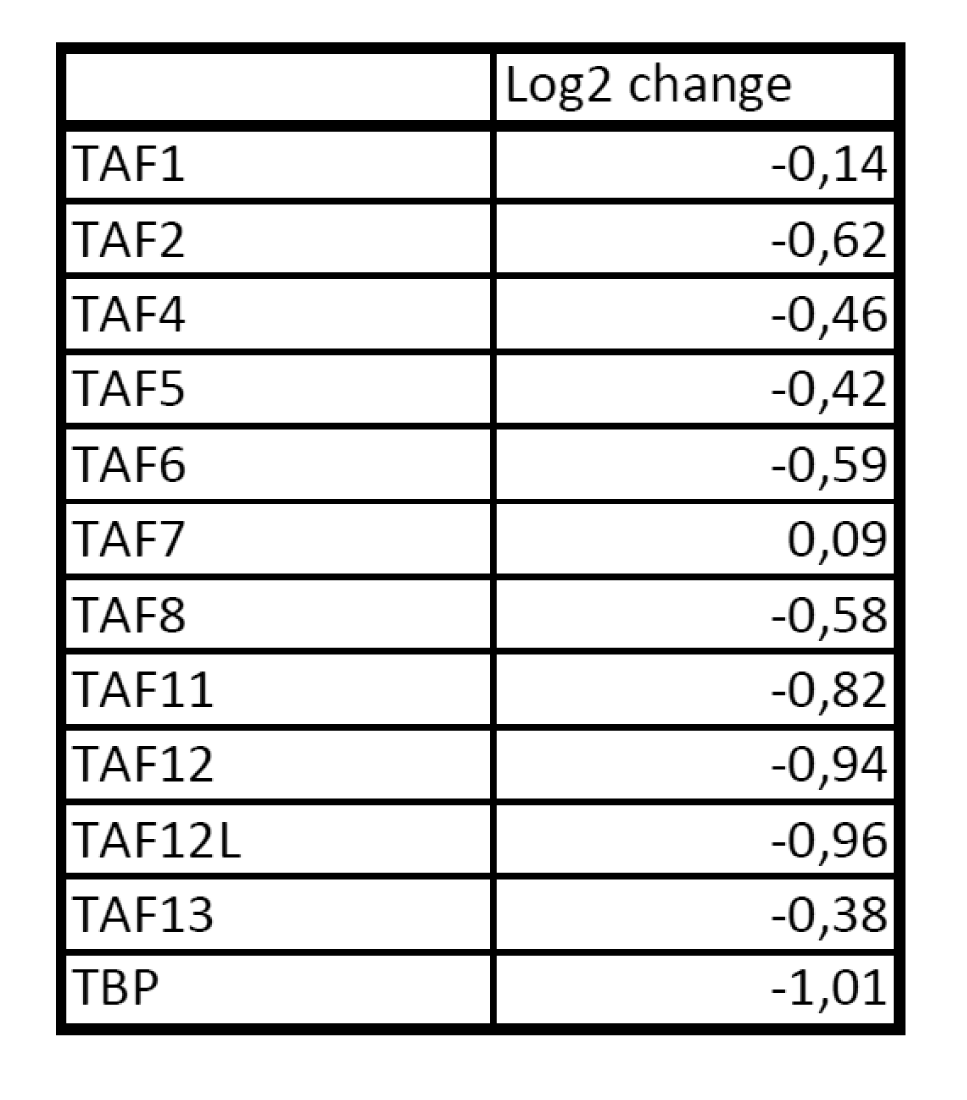

Supplement: S5 Fig — (TIF) [file pone.0142226.s005.tif]

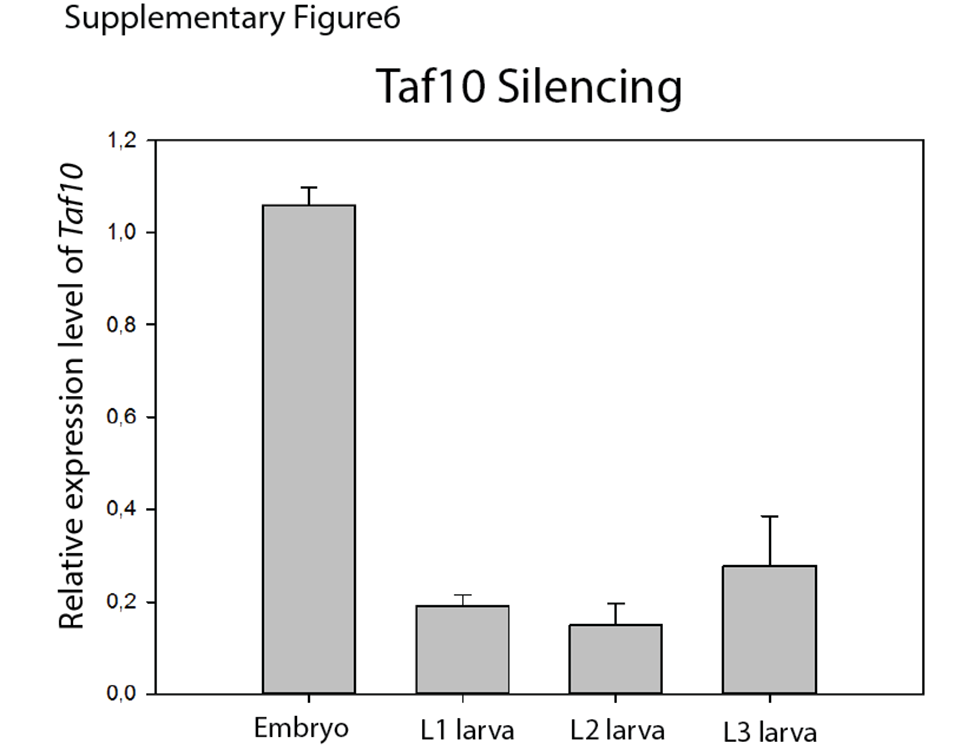

Supplement: S6 Fig — (TIF) [file pone.0142226.s006.tif]
