# Supplementary material for: dTAF10- and dTAF10b-Containing Complexes Are Required for Ecdysone-Driven Larval-Pupal Morphogenesis in Drosophila melanogaster
Source: PLoS One. 2015 Nov 10;10(11):e0142226. doi: 10.1371/journal.pone.0142226 (PMC4640578; doi:10.1371/journal.pone.0142226)
Supplement: S1 Table — (PDF) [file pone.0142226.s007.pdf]

Supplementary table 1: Gene Ontology categories of genes affected by Taf10 and dAda2a mutation with altered expression patterns compared to control.

Taf10 - Repression							
Category	Term	Number of affected genes	percentage	PValue	Bonferroni	Benjamini
GOTERM_BP_FAT	GO:0006928~cell motion	114	4,2	6,19E-19	1,58E-15	1,58E-15
GOTERM_BP_FAT	GO:0045165~cell fate commitment	93	3,4	8,13E-18	2,07E-14	1,04E-14
GOTERM_BP_FAT	GO:0000902~cell morphogenesis	144	5,3	1,50E-17	3,83E-14	1,28E-14
GOTERM_BP_FAT	GO:0048732~gland development	72	2,7	1,48E-16	2,83E-13	7,07E-14
GOTERM_BP_FAT	GO:0002165~instar larval or pupal development	136	5,0	2,63E-16	5,66E-13	1,13E-13
GOTERM_BP_FAT	GO:0009791~post-embryonic development	139	5,2	5,11E-16	1,41E-12	2,36E-13
GOTERM_BP_FAT	GO:0009886~post-embryonic morphogenesis	120	4,4	5,30E-16	1,41E-12	2,02E-13
GOTERM_BP_FAT	GO:0030182~neuron differentiation	133	4,9	5,81E-16	1,41E-12	1,77E-13
GOTERM_BP_FAT	GO:0048707~instar larval or pupal morphogenesis	118	4,4	8,78E-16	2,26E-12	2,52E-13
GOTERM_BP_FAT	GO:0007552~metamorphosis	121	4,5	1,50E-15	3,96E-12	3,96E-13
GOTERM_BP_FAT	GO:0030030~cell projection organization	121	4,5	2,99E-15	7,64E-12	6,95E-13
GOTERM_BP_FAT	GO:0032989~cellular component morphogenesis	155	5,7	6,27E-15	1,58E-11	1,32E-12
GOTERM_BP_FAT	GO:0048666~neuron development	114	4,2	4,36E-14	1,11E-10	8,56E-12
GOTERM_BP_FAT	GO:0007444~imaginal disc development	129	4,8	4,37E-14	1,12E-10	7,96E-12
GOTERM_BP_FAT	GO:0000904~cell morphogenesis involved in differentiation	103	3,8	7,68E-14	1,96E-10	1,31E-11
GOTERM_BP_FAT	GO:0035272~exocrine system development	60	2,2	2,87E-13	7,32E-10	4,58E-11
GOTERM_BP_FAT	GO:0007431~salivary gland development	60	2,2	2,87E-13	7,32E-10	4,58E-11
GOTERM_BP_FAT	GO:0007409~axonogenesis	75	2,8	7,01E-13	1,79E-09	1,05E-10
GOTERM_BP_FAT	GO:0035218~leg disc development	32	1,2	1,11E-12	2,82E-09	1,57E-10
GOTERM_BP_FAT	GO:0048667~cell morphogenesis involved in neuron differentiation	96	3,6	2,25E-12	5,73E-09	3,02E-10
GOTERM_BP_FAT	GO:0007560~imaginal disc morphogenesis	95	3,5	2,36E-12	6,03E-09	3,01E-10
GOTERM_BP_FAT	GO:0048563~post-embryonic organ morphogenesis	95	3,5	2,36E-12	6,03E-09	3,01E-10
GOTERM_BP_FAT	GO:0048569~post-embryonic organ development	98	3,6	2,54E-12	6,46E-09	3,08E-10
GOTERM_BP_FAT	GO:0048858~cell projection morphogenesis	103	3,8	3,60E-12	9,17E-09	4,17E-10
GOTERM_BP_FAT	GO:0048812~neuron projection morphogenesis	95	3,5	3,74E-12	9,53E-09	4,14E-10
GOTERM_BP_FAT	GO:0031175~neuron projection development	95	3,5	4,69E-12	1,20E-08	4,98E-10
GOTERM_BP_FAT	GO:0007411~axon guidance	57	2,1	5,22E-12	1,33E-08	5,32E-10
GOTERM_BP_FAT	GO:0032990~cell part morphogenesis	105	3,9	5,87E-12	1,50E-08	5,76E-10
GOTERM_BP_FAT	GO:0007389~pattern specification process	137	5,1	1,46E-11	3,71E-08	1,37E-09
GOTERM_BP_FAT	GO:0045449~regulation of transcription	202	7,5	3,08E-11	7,85E-08	2,80E-09
GOTERM_BP_FAT	GO:0007435~salivary gland morphogenesis	49	1,8	6,88E-11	1,75E-07	6,05E-09
GOTERM_BP_FAT	GO:0022612~gland morphogenesis	49	1,8	6,88E-11	1,75E-07	6,05E-09
GOTERM_BP_FAT	GO:0003002~regionalization	129	4,8	8,87E-11	2,26E-07	7,54E-09
GOTERM_BP_FAT	GO:0007423~sensory organ development	119	4,4	1,26E-10	3,21E-07	1,03E-08
GOTERM_BP_FAT	GO:0051252~regulation of RNA metabolic process	175	6,5	1,52E-10	3,88E-07	1,21E-08
GOTERM_BP_FAT	GO:0007447~imaginal disc pattern formation	43	1,6	2,53E-10	6,45E-07	1,95E-08
GOTERM_BP_FAT	GO:0048736~appendage development	81	3,0	2,89E-10	7,36E-07	2,16E-08
GOTERM_BP_FAT	GO:0048737~imaginal disc-derived appendage development	80	3,0	3,81E-10	9,70E-07	2,77E-08
GOTERM_BP_FAT	GO:0060173~limb development	25	0,9	6,05E-10	1,54E-06	4,28E-08
GOTERM_BP_FAT	GO:0035108~limb morphogenesis	25	0,9	6,05E-10	1,54E-06	4,28E-08
GOTERM_BP_FAT	GO:0048870~cell motility	66	2,4	6,60E-10	1,68E-06	4,55E-08
GOTERM_BP_FAT	GO:0016477~cell migration	63	2,3	6,87E-10	1,75E-06	4,61E-08
GOTERM_BP_FAT	GO:0006355~regulation of transcription, DNA-dependent	159	5,9	7,55E-10	1,92E-06	4,94E-08
GOTERM_BP_FAT	GO:0035107~appendage morphogenesis	79	2,9	7,78E-10	1,98E-06	4,96E-08
GOTERM_BP_FAT	GO:0051674~localization of cell	68	2,5	8,08E-10	2,06E-06	5,02E-08
GOTERM_BP_FAT	GO:0035114~imaginal disc-derived appendage morphogenesis	78	2,9	1,03E-09	2,61E-06	6,22E-08
GOTERM_BP_FAT	GO:0035110~leg morphogenesis	24	0,9	2,24E-09	5,70E-06	1,33E-07
GOTERM_BP_FAT	GO:0007155~cell adhesion	60	2,2	1,18E-08	3,01E-05	6,85E-07
GOTERM_BP_FAT	GO:0048729~tissue morphogenesis	77	2,9	1,30E-08	3,32E-05	7,39E-07
GOTERM_BP_FAT	GO:0009954~proximal/distal pattern formation	17	0,6	2,19E-08	5,59E-05	1,22E-06
GOTERM_BP_FAT	GO:0007478~leg disc morphogenesis	20	0,7	2,36E-08	6,01E-05	1,28E-06
GOTERM_BP_FAT	GO:0001708~cell fate specification	30	1,1	2,45E-08	6,25E-05	1,30E-06
GOTERM_BP_FAT	GO:0006350~transcription	122	4,5	2,50E-08	6,37E-05	1,30E-06
GOTERM_BP_FAT	GO:0001709~cell fate determination	48	1,8	2,99E-08	7,62E-05	1,52E-06
GOTERM_BP_FAT	GO:0007498~mesoderm development	38	1,4	3,26E-08	8,32E-05	1,63E-06
GOTERM_BP_FAT	GO:0007449~proximal/distal pattern formation, imaginal disc	16	0,6	3,52E-08	8,96E-05	1,72E-06
GOTERM_BP_FAT	GO:0016265~death	56	2,1	4,50E-08	1,15E-04	2,16E-06
GOTERM_BP_FAT	GO:0007166~cell surface receptor linked signal transduction	155	5,7	5,62E-08	1,43E-04	2,65E-06
GOTERM_BP_FAT	GO:0035120~post-embryonic appendage morphogenesis	71	2,6	6,62E-08	1,69E-04	3,07E-06
GOTERM_BP_FAT	GO:0012501~programmed cell death	53	2,0	8,03E-08	2,05E-04	3,65E-06
GOTERM_BP_FAT	GO:0060541~respiratory system development	53	2,0	8,03E-08	2,05E-04	3,65E-06
GOTERM_BP_FAT	GO:0007424~open tracheal system development	53	2,0	8,03E-08	2,05E-04	3,65E-06
GOTERM_BP_FAT	GO:0035109~imaginal disc-derived limb morphogenesis	19	0,7	8,90E-08	2,27E-04	3,98E-06
GOTERM_BP_FAT	GO:0008219~cell death	55	2,0	9,48E-08	2,42E-04	4,16E-06
GOTERM_BP_FAT	GO:0048749~compound eye development	88	3,3	9,93E-08	2,53E-04	4,29E-06
GOTERM_BP_FAT	GO:0007507~heart development	31	1,1	1,10E-07	2,81E-04	4,69E-06
GOTERM_BP_FAT	GO:0001654~eye development	93	3,4	1,13E-07	2,88E-04	4,72E-06
GOTERM_BP_FAT	GO:0016337~cell-cell adhesion	28	1,0	1,31E-07	3,35E-04	5,41E-06
GOTERM_BP_FAT	GO:0007156~homophilic cell adhesion	18	0,7	1,61E-07	4,09E-04	6,50E-06
GOTERM_BP_FAT	GO:0002009~morphogenesis of an epithelium	69	2,6	1,66E-07	4,23E-04	6,60E-06
GOTERM_BP_FAT	GO:0060429~epithelium development	71	2,6	2,03E-07	5,18E-04	7,98E-06
GOTERM_BP_FAT	GO:0006357~regulation of transcription from RNA polymerase II promoter	61	2,3	2,49E-07	6,33E-04	9,60E-06
GOTERM_BP_FAT	GO:0022610~biological adhesion	60	2,2	2,66E-07	6,78E-04	1,01E-05
GOTERM_BP_FAT	GO:0007365~periodic partitioning	26	1,0	2,91E-07	7,41E-04	1,09E-05
GOTERM_BP_FAT	GO:0007480~imaginal disc-derived leg morphogenesis	18	0,7	3,28E-07	8,37E-04	1,21E-05
GOTERM_BP_FAT	GO:0035127~post-embryonic limb morphogenesis	18	0,7	3,28E-07	8,37E-04	1,21E-05
GOTERM_BP_FAT	GO:0035220~wing disc development	80	3,0	4,83E-07	1,23E-03	1,76E-05
GOTERM_BP_FAT	GO:0007548~sex differentiation	27	1,0	8,38E-07	2,13E-03	3,01E-05
GOTERM_BP_FAT	GO:0000122~negative regulation of transcription from RNA polymerase II promoter	27	1,0	1,23E-06	3,14E-03	4,37E-05
GOTERM_BP_FAT	GO:0048859~formation of anatomical boundary	22	0,8	1,40E-06	3,55E-03	4,88E-05
GOTERM_BP_FAT	GO:0007219~Notch signaling pathway	26	1,0	1,53E-06	3,90E-03	5,29E-05
GOTERM_BP_FAT	GO:0035214~eye-antennal disc development	27	1,0	1,79E-06	4,56E-03	6,10E-05
GOTERM_BP_FAT	GO:0008038~neuron recognition	23	0,9	2,85E-06	7,24E-03	9,56E-05
GOTERM_BP_FAT	GO:0016055~Wnt receptor signaling pathway	29	1,1	3,14E-06	7,98E-03	1,04E-04
GOTERM_BP_FAT	GO:0045596~negative regulation of cell differentiation	30	1,1	3,37E-06	8,54E-03	1,10E-04
GOTERM_BP_FAT	GO:0007398~ectoderm development	28	1,0	4,03E-06	1,02E-02	1,30E-04
GOTERM_BP_FAT	GO:0008037~cell recognition	23	0,9	4,30E-06	1,09E-02	1,37E-04
GOTERM_BP_FAT	GO:0007479~leg disc proximal/distal pattern formation	12	0,4	4,71E-06	1,19E-02	1,48E-04
GOTERM_BP_FAT	GO:0035223~leg disc pattern formation	12	0,4	4,71E-06	1,19E-02	1,48E-04

GOTERM_BP_FAT	GO:0048645~organ formation	20	0,7	4,87E-06	1,23E-02	1,51E-04
GOTERM_BP_FAT	GO:0007472~wing disc morphogenesis	63	2,3	7,63E-06	1,93E-02	2,34E-04
GOTERM_BP_FAT	GO:0001738~morphogenesis of a polarized epithelium	28	1,0	7,70E-06	1,94E-02	2,34E-04
GOTERM_BP_FAT	GO:0016271~tissue death	28	1,0	7,70E-06	1,94E-02	2,34E-04
GOTERM_BP_FAT	GO:0007559~histolysis	28	1,0	7,70E-06	1,94E-02	2,34E-04
GOTERM_BP_FAT	GO:0010623~developmental programmed cell death	11	0,4	7,77E-06	1,96E-02	2,33E-04
GOTERM_BP_FAT	GO:0048598~embryonic morphogenesis	64	2,4	9,48E-06	2,39E-02	2,81E-04
GOTERM_BP_FAT	GO:0007476~imaginal disc-derived wing morphogenesis	62	2,3	1,15E-05	2,90E-02	3,38E-04
GOTERM_BP_FAT	GO:0035222~wing disc pattern formation	27	1,0	1,36E-05	3,41E-02	3,94E-04
GOTERM_BP_FAT	GO:0042067~establishment of ommatidial polarity	21	0,8	1,46E-05	3,65E-02	4,17E-04
GOTERM_BP_FAT	GO:0007610~behavior	105	3,9	1,62E-05	4,05E-02	4,59E-04
GOTERM_BP_FAT	GO:0007163~establishment or maintenance of cell polarity	37	1,4	1,63E-05	4,08E-02	4,57E-04
GOTERM_BP_FAT	GO:0001736~establishment of planar polarity	25	0,9	1,65E-05	4,12E-02	4,58E-04
GOTERM_BP_FAT	GO:0045137~development of primary sexual characteristics	18	0,7	1,70E-05	4,24E-02	4,65E-04
GOTERM_BP_FAT	GO:0007164~establishment of tissue polarity	25	0,9	2,28E-05	5,64E-02	6,18E-04
GOTERM_BP_FAT	GO:0007626~locomotory behavior	53	2,0	2,30E-05	5,71E-02	6,18E-04
GOTERM_BP_FAT	GO:0010160~formation of organ boundary	18	0,7	4,08E-05	9,88E-02	1,08E-03
GOTERM_BP_FAT	GO:0035215~genital disc development	15	0,6	4,27E-05	1,03E-01	1,12E-03
GOTERM_BP_FAT	GO:0035071~salivary gland cell autophagic cell death	26	1,0	4,32E-05	1,04E-01	1,12E-03
GOTERM_BP_FAT	GO:0048102~autophagic cell death	26	1,0	4,32E-05	1,04E-01	1,12E-03
GOTERM_BP_FAT	GO:0035070~salivary gland histolysis	26	1,0	4,32E-05	1,04E-01	1,12E-03
GOTERM_BP_FAT	GO:0051301~cell division	57	2,1	5,48E-05	1,30E-01	1,41E-03
GOTERM_BP_FAT	GO:0048565~gut development	27	1,0	5,74E-05	1,36E-01	1,46E-03
GOTERM_BP_FAT	GO:0045944~positive regulation of transcription from RNA polymerase II promoter	21	0,8	6,28E-05	1,48E-01	1,58E-03
GOTERM_BP_FAT	GO:0001751~compound eye photoreceptor cell differentiation	36	1,3	6,38E-05	1,50E-01	1,59E-03
GOTERM_BP_FAT	GO:0051254~positive regulation of RNA metabolic process	31	1,1	6,70E-05	1,57E-01	1,66E-03
GOTERM_BP_FAT	GO:0035282~segmentation	68	2,5	7,79E-05	1,80E-01	1,91E-03
GOTERM_BP_FAT	GO:0007469~antennal development	13	0,5	8,95E-05	2,04E-01	2,17E-03
GOTERM_BP_FAT	GO:0035285~appendage segmentation	11	0,4	9,33E-05	2,12E-01	2,24E-03
GOTERM_BP_FAT	GO:0035286~leg segmentation	11	0,4	9,33E-05	2,12E-01	2,24E-03
GOTERM_BP_FAT	GO:0042330~taxis	19	0,7	1,03E-04	2,31E-01	2,45E-03
GOTERM_BP_FAT	GO:0045893~positive regulation of transcription, DNA-dependent	30	1,1	1,11E-04	2,47E-01	2,62E-03
GOTERM_BP_FAT	GO:0046530~photoreceptor cell differentiation	39	1,4	1,34E-04	2,89E-01	3,12E-03
GOTERM_BP_FAT	GO:0009880~embryonic pattern specification	61	2,3	1,44E-04	3,07E-01	3,33E-03
GOTERM_BP_FAT	GO:0010942~positive regulation of cell death	19	0,7	1,45E-04	3,09E-01	3,33E-03
GOTERM_BP_FAT	GO:0043068~positive regulation of programmed cell death	19	0,7	1,45E-04	3,09E-01	3,33E-03
GOTERM_BP_FAT	GO:0010604~positive regulation of macromolecule metabolic process	42	1,6	1,46E-04	3,11E-01	3,32E-03
GOTERM_BP_FAT	GO:0001754~eye photoreceptor cell differentiation	36	1,3	1,71E-04	3,54E-01	3,85E-03
GOTERM_BP_FAT	GO:0003006~reproductive developmental process	115	4,3	1,83E-04	3,73E-01	4,08E-03
GOTERM_BP_FAT	GO:0035295~tube development	32	1,2	1,89E-04	3,83E-01	4,19E-03
GOTERM_BP_FAT	GO:0007059~chromosome segregation	36	1,3	2,06E-04	4,09E-01	4,52E-03
GOTERM_BP_FAT	GO:0048511~rhythmic process	20	0,7	2,13E-04	4,19E-01	4,64E-03
GOTERM_BP_FAT	GO:0048592~eye morphogenesis	68	2,5	2,31E-04	4,44E-01	4,97E-03
GOTERM_BP_FAT	GO:0007367~segment polarity determination	17	0,6	2,38E-04	4,55E-01	5,09E-03
GOTERM_BP_FAT	GO:0001745~compound eye morphogenesis	64	2,4	2,49E-04	4,70E-01	5,28E-03
GOTERM_BP_FAT	GO:0035017~cuticle pattern formation	13	0,5	2,49E-04	4,70E-01	5,23E-03
GOTERM_BP_FAT	GO:0007422~peripheral nervous system development	29	1,1	2,83E-04	5,14E-01	5,90E-03
GOTERM_BP_FAT	GO:0007280~pole cell migration	11	0,4	3,22E-04	5,60E-01	6,65E-03
GOTERM_BP_FAT	GO:0007350~blastoderm segmentation	57	2,1	3,22E-04	5,60E-01	6,60E-03
GOTERM_BP_FAT	GO:0048103~somatic stem cell division	14	0,5	3,23E-04	5,61E-01	6,56E-03
GOTERM_BP_FAT	GO:0009968~negative regulation of signal transduction	32	1,2	3,43E-04	5,83E-01	6,91E-03
GOTERM_BP_FAT	GO:0043067~regulation of programmed cell death	34	1,3	3,51E-04	5,92E-01	7,03E-03
GOTERM_BP_FAT	GO:0010648~negative regulation of cell communication	32	1,2	4,14E-04	6,52E-01	8,22E-03
GOTERM_BP_FAT	GO:0007611~learning or memory	26	1,0	4,18E-04	6,56E-01	8,24E-03
GOTERM_BP_FAT	GO:0008406~gonad development	14	0,5	4,84E-04	7,09E-01	9,44E-03
GOTERM_BP_FAT	GO:0048608~reproductive structure development	14	0,5	4,84E-04	7,09E-01	9,44E-03
GOTERM_BP_FAT	GO:0007450~dorsal/ventral pattern formation, imaginal disc	21	0,8	5,04E-04	7,23E-01	9,75E-03
GOTERM_BP_FAT	GO:0007623~circadian rhythm	19	0,7	5,05E-04	7,24E-01	9,70E-03
GOTERM_BP_FAT	GO:0016198~axon choice point recognition	11	0,4	5,49E-04	7,53E-01	1,05E-02
GOTERM_BP_FAT	GO:0035051~cardiac cell differentiation	11	0,4	5,49E-04	7,53E-01	1,05E-02
GOTERM_BP_FAT	GO:0008354~germ cell migration	16	0,6	6,08E-04	7,88E-01	1,15E-02
GOTERM_BP_FAT	GO:0046668~regulation of retinal cell programmed cell death	10	0,4	6,14E-04	7,91E-01	1,15E-02
GOTERM_BP_FAT	GO:0035290~trunk segmentation	10	0,4	6,14E-04	7,91E-01	1,15E-02
GOTERM_BP_FAT	GO:0050890~cognition	82	3,0	6,75E-04	8,21E-01	1,26E-02
GOTERM_BP_FAT	GO:0010941~regulation of cell death	34	1,3	7,07E-04	8,35E-01	1,31E-02
GOTERM_BP_FAT	GO:0007419~ventral cord development	14	0,5	7,09E-04	8,36E-01	1,30E-02
GOTERM_BP_FAT	GO:0010557~positive regulation of macromolecule biosynthetic process	37	1,4	7,34E-04	8,46E-01	1,34E-02
GOTERM_BP_FAT	GO:0045941~positive regulation of transcription	34	1,3	8,35E-04	8,81E-01	1,51E-02
GOTERM_BP_FAT	GO:0007612~learning	20	0,7	8,60E-04	8,88E-01	1,54E-02
GOTERM_BP_FAT	GO:0017145~stem cell division	19	0,7	8,76E-04	8,93E-01	1,56E-02
GOTERM_BP_FAT	GO:0051173~positive regulation of nitrogen compound metabolic process	35	1,3	8,92E-04	8,97E-01	1,58E-02
GOTERM_BP_FAT	GO:0045935~positive regulation of nucleobase, nucleoside, nucleotide and nucleic acid metabolic process	35	1,3	8,92E-04	8,97E-01	1,58E-02
GOTERM_BP_FAT	GO:0014016~neuroblast differentiation	13	0,5	8,98E-04	8,99E-01	1,58E-02
GOTERM_BP_FAT	GO:0010628~positive regulation of gene expression	34	1,3	9,82E-04	9,18E-01	1,71E-02
GOTERM_BP_FAT	GO:0043473~pigmentation	22	0,8	1,01E-03	9,24E-01	1,75E-02
GOTERM_BP_FAT	GO:0007622~rhythmic behavior	16	0,6	1,14E-03	9,45E-01	1,96E-02
GOTERM_BP_FAT	GO:0008544~epidermis development	18	0,7	1,16E-03	9,48E-01	1,97E-02
GOTERM_BP_FAT	GO:0055057~neuroblast division	11	0,4	1,40E-03	9,72E-01	2,37E-02
GOTERM_BP_FAT	GO:0055059~asymmetric neuroblast division	11	0,4	1,40E-03	9,72E-01	2,37E-02
GOTERM_BP_FAT	GO:0048534~hemopoietic or lymphoid organ development	20	0,7	1,40E-03	9,72E-01	2,36E-02
GOTERM_BP_FAT	GO:0002520~immune system development	20	0,7	1,40E-03	9,72E-01	2,36E-02
GOTERM_BP_FAT	GO:0008258~head involution	18	0,7	1,50E-03	9,78E-01	2,51E-02
GOTERM_BP_FAT	GO:0007379~segment specification	17	0,6	1,53E-03	9,80E-01	2,53E-02
GOTERM_BP_FAT	GO:0035277~spiracle morphogenesis, open tracheal system	10	0,4	1,70E-03	9,87E-01	2,80E-02
GOTERM_BP_FAT	GO:0007517~muscle organ development	38	1,4	1,85E-03	9,91E-01	3,02E-02
GOTERM_BP_FAT	GO:0006468~protein amino acid phosphorylation	62	2,3	1,93E-03	9,93E-01	3,13E-02
GOTERM_BP_FAT	GO:0007405~neuroblast proliferation	14	0,5	1,96E-03	9,93E-01	3,16E-02
GOTERM_BP_FAT	GO:0048512~circadian behavior	15	0,6	2,01E-03	9,94E-01	3,22E-02
GOTERM_BP_FAT	GO:0008052~sensory organ boundary specification	11	0,4	2,11E-03	9,95E-01	3,35E-02
GOTERM_BP_FAT	GO:0051345~positive regulation of hydrolase activity	11	0,4	2,11E-03	9,95E-01	3,35E-02
GOTERM_BP_FAT	GO:0007017~microtubule-based process	90	3,3	2,23E-03	9,97E-01	3,52E-02
GOTERM_BP_FAT	GO:0014017~neuroblast fate commitment	12	0,4	2,40E-03	9,98E-01	3,75E-02
GOTERM_BP_FAT	GO:0045448~mitotic cell cycle, embryonic	12	0,4	2,40E-03	9,98E-01	3,75E-02
GOTERM_BP_FAT	GO:0010721~negative regulation of cell development	10	0,4	2,64E-03	9,99E-01	4,11E-02
GOTERM_BP_FAT	GO:0042659~regulation of cell fate specification	10	0,4	2,64E-03	9,99E-01	4,11E-02

GOTERM_BP_FAT	GO:0010453~regulation of cell fate commitment	10	0,4	2,64E-03	9,99E-01	4,11E-02
GOTERM_BP_FAT	GO:0030178~negative regulation of Wnt receptor signaling pathway	10	0,4	2,64E-03	9,99E-01	4,11E-02
GOTERM_BP_FAT	GO:0007448~anterior/posterior pattern formation, imaginal disc	10	0,4	2,64E-03	9,99E-01	4,11E-02
GOTERM_BP_FAT	GO:0007455~eye-antennal disc morphogenesis	15	0,6	2,67E-03	9,99E-01	4,12E-02
GOTERM_BP_FAT	GO:0009891~positive regulation of biosynthetic process	40	1,5	2,87E-03	9,99E-01	4,39E-02
GOTERM_BP_FAT	GO:0031328~positive regulation of cellular biosynthetic process	40	1,5	2,87E-03	9,99E-01	4,39E-02
GOTERM_BP_FAT	GO:0048542~lymph gland development	11	0,4	3,09E-03	1,00E+00	4,70E-02
GOTERM_BP_FAT	GO:0042063~gliogenesis	17	0,6	3,26E-03	1,00E+00	4,93E-02
GOTERM_BP_FAT	GO:0055123~digestive system development	17	0,6	3,26E-03	1,00E+00	4,93E-02
GOTERM_BP_FAT	GO:0048546~digestive tract morphogenesis	17	0,6	3,26E-03	1,00E+00	4,93E-02
GOTERM_BP_FAT	GO:0042981~regulation of apoptosis	28	1,0	3,35E-03	1,00E+00	5,02E-02
GOTERM_BP_FAT	GO:0007494~midgut development	12	0,4	3,37E-03	1,00E+00	5,02E-02
GOTERM_BP_FAT	GO:0009913~epidermal cell differentiation	14	0,5	3,54E-03	1,00E+00	5,24E-02
GOTERM_BP_FAT	GO:0045132~meiotic chromosome segregation	19	0,7	3,63E-03	1,00E+00	5,34E-02
GOTERM_BP_FAT	GO:0035239~tube morphogenesis	25	0,9	3,82E-03	1,00E+00	5,58E-02
GOTERM_BP_FAT	GO:0035287~head segmentation	10	0,4	3,96E-03	1,00E+00	5,74E-02
GOTERM_BP_FAT	GO:0046700~heterocycle catabolic process	10	0,4	3,96E-03	1,00E+00	5,74E-02
GOTERM_BP_FAT	GO:0051726~regulation of cell cycle	42	1,6	4,19E-03	1,00E+00	6,03E-02
GOTERM_BP_FAT	GO:0048589~developmental growth	24	0,9	4,20E-03	1,00E+00	6,02E-02
GOTERM_BP_FAT	GO:0008593~regulation of Notch signaling pathway	11	0,4	4,40E-03	1,00E+00	6,25E-02
GOTERM_BP_FAT	GO:0010631~epithelial cell migration	12	0,4	4,63E-03	1,00E+00	6,53E-02
GOTERM_BP_FAT	GO:0007427~epithelial cell migration, open tracheal system	12	0,4	4,63E-03	1,00E+00	6,53E-02
GOTERM_BP_FAT	GO:0016481~negative regulation of transcription	50	1,9	4,63E-03	1,00E+00	6,50E-02
GOTERM_BP_FAT	GO:0019827~stem cell maintenance	13	0,5	4,70E-03	1,00E+00	6,55E-02
GOTERM_BP_FAT	GO:0048663~neuron fate commitment	18	0,7	4,81E-03	1,00E+00	6,67E-02
GOTERM_BP_FAT	GO:0051172~negative regulation of nitrogen compound metabolic process	53	2,0	4,98E-03	1,00E+00	6,86E-02
GOTERM_BP_FAT	GO:0045934~negative regulation of nucleobase, nucleoside, nucleotide and nucleic acid metabolic process	53	2,0	4,98E-03	1,00E+00	6,86E-02
GOTERM_BP_FAT	GO:0007293~germarium-derived egg chamber formation	20	0,7	5,03E-03	1,00E+00	6,89E-02
GOTERM_BP_FAT	GO:0048190~wing disc dorsal/ventral pattern formation	17	0,6	5,15E-03	1,00E+00	7,01E-02
GOTERM_BP_FAT	GO:0007439~ectodermal gut development	16	0,6	5,47E-03	1,00E+00	7,40E-02
GOTERM_BP_FAT	GO:0048567~ectodermal gut morphogenesis	16	0,6	5,47E-03	1,00E+00	7,40E-02
GOTERM_BP_FAT	GO:0007442~hindgut morphogenesis	15	0,6	5,77E-03	1,00E+00	7,74E-02
GOTERM_BP_FAT	GO:0022416~bristle development	18	0,7	5,92E-03	1,00E+00	7,89E-02
GOTERM_BP_FAT	GO:0016331~morphogenesis of embryonic epithelium	32	1,2	5,98E-03	1,00E+00	7,93E-02
GOTERM_BP_FAT	GO:0051276~chromosome organization	64	2,4	6,04E-03	1,00E+00	7,96E-02
GOTERM_BP_FAT	GO:0042335~cuticle development	20	0,7	6,07E-03	1,00E+00	7,97E-02
GOTERM_BP_FAT	GO:0048864~stem cell development	13	0,5	6,19E-03	1,00E+00	8,07E-02
GOTERM_BP_FAT	GO:0035316~non-sensory hair organization	13	0,5	6,19E-03	1,00E+00	8,07E-02
GOTERM_BP_FAT	GO:0012502~induction of programmed cell death	13	0,5	6,19E-03	1,00E+00	8,07E-02
GOTERM_BP_FAT	GO:0048863~stem cell differentiation	13	0,5	6,19E-03	1,00E+00	8,07E-02
GOTERM_BP_FAT	GO:0035315~hair cell differentiation	13	0,5	6,19E-03	1,00E+00	8,07E-02
GOTERM_BP_FAT	GO:0046552~photoreceptor cell fate commitment	17	0,6	6,38E-03	1,00E+00	8,26E-02
GOTERM_BP_FAT	GO:0007297~ovarian follicle cell migration	23	0,9	6,47E-03	1,00E+00	8,34E-02
GOTERM_BP_FAT	GO:0007283~spermatogenesis	39	1,4	6,49E-03	1,00E+00	8,32E-02
GOTERM_BP_FAT	GO:0048232~male gamete generation	39	1,4	6,49E-03	1,00E+00	8,32E-02
GOTERM_BP_FAT	GO:0050877~neurological system process	113	4,2	6,55E-03	1,00E+00	8,35E-02
GOTERM_BP_FAT	GO:0008407~bristle morphogenesis	16	0,6	6,84E-03	1,00E+00	8,66E-02
GOTERM_BP_FAT	GO:0001752~compound eye photoreceptor fate commitment	16	0,6	6,84E-03	1,00E+00	8,66E-02
GOTERM_BP_FAT	GO:0048547~gut morphogenesis	16	0,6	6,84E-03	1,00E+00	8,66E-02
GOTERM_BP_FAT	GO:0042706~eye photoreceptor cell fate commitment	16	0,6	6,84E-03	1,00E+00	8,66E-02
GOTERM_BP_FAT	GO:0048813~dendrite morphogenesis	31	1,1	7,58E-03	1,00E+00	9,51E-02
GOTERM_BP_FAT	GO:0016358~dendrite development	31	1,1	7,58E-03	1,00E+00	9,51E-02
GOTERM_BP_FAT	GO:0048066~pigmentation during development	19	0,7	7,99E-03	1,00E+00	9,95E-02
GOTERM_BP_FAT	GO:0033157~regulation of intracellular protein transport	12	0,4	8,25E-03	1,00E+00	1,02E-01
GOTERM_BP_FAT	GO:0045475~locomotor rhythm	11	0,4	8,29E-03	1,00E+00	1,02E-01
GOTERM_BP_FAT	GO:0007298~border follicle cell migration	20	0,7	8,69E-03	1,00E+00	1,06E-01
GOTERM_BP_FAT	GO:0030707~ovarian follicle cell development	48	1,8	9,24E-03	1,00E+00	1,12E-01
GOTERM_BP_FAT	GO:0042051~compound eye photoreceptor development	17	0,6	9,54E-03	1,00E+00	1,15E-01
GOTERM_BP_FAT	GO:0007391~dorsal closure	26	1,0	1,00E-02	1,00E+00	1,20E-01
GOTERM_BP_FAT	GO:0008356~asymmetric cell division	18	0,7	1,05E-02	1,00E+00	1,25E-01
GOTERM_BP_FAT	GO:0001667~ameboidal cell migration	12	0,4	1,07E-02	1,00E+00	1,27E-01
GOTERM_BP_FAT	GO:0043632~modification-dependent macromolecule catabolic process	41	1,5	1,09E-02	1,00E+00	1,28E-01
GOTERM_BP_FAT	GO:0042752~regulation of circadian rhythm	11	0,4	1,10E-02	1,00E+00	1,29E-01
GOTERM_BP_FAT	GO:0030855~epithelial cell differentiation	10	0,4	1,10E-02	1,00E+00	1,28E-01
GOTERM_BP_FAT	GO:0007167~enzyme linked receptor protein signaling pathway	36	1,3	1,12E-02	1,00E+00	1,29E-01
GOTERM_BP_FAT	GO:0042461~photoreceptor cell development	19	0,7	1,14E-02	1,00E+00	1,31E-01
GOTERM_BP_FAT	GO:0007143~female meiosis	19	0,7	1,14E-02	1,00E+00	1,31E-01
GOTERM_BP_FAT	GO:0006915~apoptosis	22	0,8	1,15E-02	1,00E+00	1,32E-01
GOTERM_BP_FAT	GO:0042462~eye photoreceptor cell development	17	0,6	1,15E-02	1,00E+00	1,31E-01
GOTERM_BP_FAT	GO:0031327~negative regulation of cellular biosynthetic process	56	2,1	1,24E-02	1,00E+00	1,40E-01
GOTERM_BP_FAT	GO:0009890~negative regulation of biosynthetic process	56	2,1	1,24E-02	1,00E+00	1,40E-01
GOTERM_BP_FAT	GO:0060284~regulation of cell development	33	1,2	1,25E-02	1,00E+00	1,40E-01
GOTERM_BP_FAT	GO:0007010~cytoskeleton organization	96	3,6	1,31E-02	1,00E+00	1,46E-01
GOTERM_BP_FAT	GO:0009792~embryonic development ending in birth or egg hatching	51	1,9	1,37E-02	1,00E+00	1,51E-01
GOTERM_BP_FAT	GO:0070201~regulation of establishment of protein localization	12	0,4	1,38E-02	1,00E+00	1,51E-01
GOTERM_BP_FAT	GO:0035317~imaginal disc-derived wing hair organization	12	0,4	1,38E-02	1,00E+00	1,51E-01
GOTERM_BP_FAT	GO:0051223~regulation of protein transport	12	0,4	1,38E-02	1,00E+00	1,51E-01
GOTERM_BP_FAT	GO:0046822~regulation of nucleocytoplasmic transport	12	0,4	1,38E-02	1,00E+00	1,51E-01
GOTERM_BP_FAT	GO:0008283~cell proliferation	32	1,2	1,39E-02	1,00E+00	1,53E-01
GOTERM_BP_FAT	GO:0001700~embryonic development via the syncytial blastoderm	49	1,8	1,42E-02	1,00E+00	1,54E-01
GOTERM_BP_FAT	GO:0051603~proteolysis involved in cellular protein catabolic process	43	1,6	1,42E-02	1,00E+00	1,54E-01
GOTERM_BP_FAT	GO:0044257~cellular protein catabolic process	43	1,6	1,42E-02	1,00E+00	1,54E-01
GOTERM_BP_FAT	GO:0007400~neuroblast fate determination	10	0,4	1,48E-02	1,00E+00	1,59E-01
GOTERM_BP_FAT	GO:0016318~ommatidial rotation	10	0,4	1,48E-02	1,00E+00	1,59E-01
GOTERM_BP_FAT	GO:0010558~negative regulation of macromolecule biosynthetic process	55	2,0	1,48E-02	1,00E+00	1,59E-01
GOTERM_BP_FAT	GO:0042692~muscle cell differentiation	21	0,8	1,49E-02	1,00E+00	1,59E-01
GOTERM_BP_FAT	GO:0007346~regulation of mitotic cell cycle	26	1,0	1,49E-02	1,00E+00	1,58E-01
GOTERM_BP_FAT	GO:0015837~amine transport	14	0,5	1,50E-02	1,00E+00	1,58E-01
GOTERM_BP_FAT	GO:0006793~phosphorus metabolic process	111	4,1	1,54E-02	1,00E+00	1,62E-01
GOTERM_BP_FAT	GO:0006796~phosphate metabolic process	111	4,1	1,54E-02	1,00E+00	1,62E-01
GOTERM_BP_FAT	GO:0035152~regulation of tube architecture, open tracheal system	13	0,5	1,62E-02	1,00E+00	1,69E-01
GOTERM_BP_FAT	GO:0032880~regulation of protein localization	13	0,5	1,62E-02	1,00E+00	1,69E-01
GOTERM_BP_FAT	GO:0019941~modification-dependent protein catabolic process	40	1,5	1,62E-02	1,00E+00	1,69E-01
GOTERM_BP_FAT	GO:0007018~microtubule-based movement	27	1,0	1,71E-02	1,00E+00	1,76E-01
GOTERM_BP_FAT	GO:0032386~regulation of intracellular transport	12	0,4	1,74E-02	1,00E+00	1,78E-01

GOTERM_BP_FAT	GO:0030097~hemopoiesis	14	0,5	1,83E-02	1,00E+00	1,86E-01
GOTERM_BP_FAT	GO:0042306~regulation of protein import into nucleus	11	0,4	1,85E-02	1,00E+00	1,87E-01
GOTERM_BP_FAT	GO:0009953~dorsal/ventral pattern formation	33	1,2	1,92E-02	1,00E+00	1,92E-01
GOTERM_BP_FAT	GO:0007635~chemosensory behavior	28	1,0	1,94E-02	1,00E+00	1,93E-01
GOTERM_BP_FAT	GO:0040007~growth	28	1,0	1,94E-02	1,00E+00	1,93E-01
GOTERM_BP_FAT	GO:0048610~reproductive cellular process	101	3,7	1,98E-02	1,00E+00	1,96E-01
GOTERM_BP_FAT	GO:0008355~olfactory learning	15	0,6	2,01E-02	1,00E+00	1,98E-01
GOTERM_BP_FAT	GO:0006508~proteolysis	142	5,3	2,07E-02	1,00E+00	2,03E-01
GOTERM_BP_FAT	GO:0050767~regulation of neurogenesis	19	0,7	2,15E-02	1,00E+00	2,09E-01
GOTERM_BP_FAT	GO:0046942~carboxylic acid transport	16	0,6	2,16E-02	1,00E+00	2,10E-01
GOTERM_BP_FAT	GO:0015849~organic acid transport	16	0,6	2,16E-02	1,00E+00	2,10E-01
GOTERM_BP_FAT	GO:0043065~positive regulation of apoptosis	12	0,4	2,17E-02	1,00E+00	2,09E-01
GOTERM_BP_FAT	GO:0044265~cellular macromolecule catabolic process	50	1,9	2,25E-02	1,00E+00	2,15E-01
GOTERM_BP_FAT	GO:0030111~regulation of Wnt receptor signaling pathway	11	0,4	2,33E-02	1,00E+00	2,22E-01
GOTERM_BP_FAT	GO:0006865~amino acid transport	13	0,5	2,43E-02	1,00E+00	2,29E-01
GOTERM_BP_FAT	GO:0048332~mesoderm morphogenesis	10	0,4	2,49E-02	1,00E+00	2,33E-01
GOTERM_BP_FAT	GO:0030163~protein catabolic process	45	1,7	2,50E-02	1,00E+00	2,34E-01
GOTERM_BP_FAT	GO:0006325~chromatin organization	39	1,4	2,82E-02	1,00E+00	2,58E-01
GOTERM_BP_FAT	GO:0000278~mitotic cell cycle	74	2,7	2,85E-02	1,00E+00	2,60E-01
GOTERM_BP_FAT	GO:0043062~extracellular structure organization	19	0,7	2,86E-02	1,00E+00	2,60E-01
GOTERM_BP_FAT	GO:0009057~macromolecule catabolic process	59	2,2	2,96E-02	1,00E+00	2,66E-01
GOTERM_BP_FAT	GO:0007169~transmembrane receptor protein tyrosine kinase signaling pathway	27	1,0	3,01E-02	1,00E+00	2,70E-01
GOTERM_BP_FAT	GO:0010629~negative regulation of gene expression	55	2,0	3,07E-02	1,00E+00	2,73E-01
GOTERM_BP_FAT	GO:0016333~morphogenesis of follicular epithelium	10	0,4	3,15E-02	1,00E+00	2,78E-01
GOTERM_BP_FAT	GO:0030031~cell projection assembly	18	0,7	3,20E-02	1,00E+00	2,82E-01
GOTERM_BP_FAT	GO:0051253~negative regulation of RNA metabolic process	43	1,6	3,31E-02	1,00E+00	2,89E-01
GOTERM_BP_FAT	GO:0009967~positive regulation of signal transduction	15	0,6	3,34E-02	1,00E+00	2,90E-01
GOTERM_BP_FAT	GO:0010605~negative regulation of macromolecule metabolic process	66	2,4	3,35E-02	1,00E+00	2,89E-01
GOTERM_BP_FAT	GO:0042048~olfactory behavior	24	0,9	3,40E-02	1,00E+00	2,92E-01
GOTERM_BP_FAT	GO:0060562~epithelial tube morphogenesis	13	0,5	3,52E-02	1,00E+00	3,00E-01
GOTERM_BP_FAT	GO:0051783~regulation of nuclear division	13	0,5	3,52E-02	1,00E+00	3,00E-01
GOTERM_BP_FAT	GO:0001763~morphogenesis of a branching structure	13	0,5	3,52E-02	1,00E+00	3,00E-01
GOTERM_BP_FAT	GO:0007088~regulation of mitosis	13	0,5	3,52E-02	1,00E+00	3,00E-01
GOTERM_BP_FAT	GO:0007224~smoothened signaling pathway	11	0,4	3,57E-02	1,00E+00	3,03E-01
GOTERM_BP_FAT	GO:0048634~regulation of muscle development	11	0,4	3,57E-02	1,00E+00	3,03E-01
GOTERM_BP_FAT	GO:0016310~phosphorylation	85	3,2	3,98E-02	1,00E+00	3,31E-01
GOTERM_BP_FAT	GO:0006470~protein amino acid dephosphorylation	21	0,8	4,27E-02	1,00E+00	3,49E-01
GOTERM_BP_FAT	GO:0016321~female meiosis chromosome segregation	11	0,4	4,34E-02	1,00E+00	3,53E-01
GOTERM_BP_FAT	GO:0007276~gamete generation	136	5,0	4,35E-02	1,00E+00	3,52E-01
GOTERM_BP_FAT	GO:0010647~positive regulation of cell communication	15	0,6	4,52E-02	1,00E+00	3,63E-01
GOTERM_BP_FAT	GO:0040008~regulation of growth	25	0,9	4,64E-02	1,00E+00	3,69E-01
GOTERM_BP_FAT	GO:0048754~branching morphogenesis of a tube	12	0,4	4,66E-02	1,00E+00	3,69E-01
GOTERM_BP_FAT	GO:0060446~branching involved in open tracheal system development	12	0,4	4,66E-02	1,00E+00	3,69E-01
GOTERM_BP_FAT	GO:0045892~negative regulation of transcription, DNA-dependent	41	1,5	4,69E-02	1,00E+00	3,70E-01
GOTERM_BP_FAT	GO:0009628~response to abiotic stimulus	46	1,7	4,75E-02	1,00E+00	3,73E-01
GOTERM_BP_FAT	GO:0019953~sexual reproduction	139	5,2	4,80E-02	1,00E+00	3,75E-01
GOTERM_BP_FAT	GO:0048588~developmental cell growth	10	0,4	4,80E-02	1,00E+00	3,74E-01
GOTERM_BP_FAT	GO:0034329~cell junction assembly	10	0,4	4,80E-02	1,00E+00	3,74E-01
GOTERM_BP_FAT	GO:0007067~mitosis	33	1,2	5,25E-02	1,00E+00	4,00E-01
GOTERM_BP_FAT	GO:0007600~sensory perception	57	2,1	5,44E-02	1,00E+00	4,10E-01
GOTERM_BP_FAT	GO:0048638~regulation of developmental growth	12	0,4	5,50E-02	1,00E+00	4,13E-01
GOTERM_BP_FAT	GO:0007127~meiosis I	13	0,5	5,72E-02	1,00E+00	4,24E-01
GOTERM_BP_FAT	GO:0051960~regulation of nervous system development	22	0,8	5,88E-02	1,00E+00	4,32E-01
GOTERM_BP_FAT	GO:0000910~cytokinesis	20	0,7	6,00E-02	1,00E+00	4,38E-01
GOTERM_BP_FAT	GO:0008361~regulation of cell size	19	0,7	6,04E-02	1,00E+00	4,39E-01
GOTERM_BP_FAT	GO:0051146~striated muscle cell differentiation	17	0,6	6,06E-02	1,00E+00	4,39E-01
GOTERM_BP_FAT	GO:0000087~M phase of mitotic cell cycle	33	1,2	6,16E-02	1,00E+00	4,43E-01
GOTERM_BP_FAT	GO:0000280~nuclear division	33	1,2	6,16E-02	1,00E+00	4,43E-01
GOTERM_BP_FAT	GO:0007613~memory	11	0,4	6,18E-02	1,00E+00	4,43E-01
GOTERM_BP_FAT	GO:0007173~epidermal growth factor receptor signaling pathway	11	0,4	6,18E-02	1,00E+00	4,43E-01
GOTERM_BP_FAT	GO:0006511~ubiquitin-dependent protein catabolic process	23	0,9	6,41E-02	1,00E+00	4,54E-01
GOTERM_BP_FAT	GO:0016568~chromatin modification	26	1,0	6,65E-02	1,00E+00	4,66E-01
GOTERM_BP_FAT	GO:0051336~regulation of hydrolase activity	18	0,7	6,79E-02	1,00E+00	4,72E-01
GOTERM_BP_FAT	GO:0007474~imaginal disc-derived wing vein specification	10	0,4	6,94E-02	1,00E+00	4,78E-01
GOTERM_BP_FAT	GO:0048619~embryonic hindgut morphogenesis	10	0,4	6,94E-02	1,00E+00	4,78E-01
GOTERM_BP_FAT	GO:0007369~gastrulation	18	0,7	7,58E-02	1,00E+00	5,09E-01
GOTERM_BP_FAT	GO:0051235~maintenance of location	13	0,5	7,62E-02	1,00E+00	5,09E-01
GOTERM_BP_FAT	GO:0000819~sister chromatid segregation	13	0,5	7,62E-02	1,00E+00	5,09E-01
GOTERM_BP_FAT	GO:0060538~skeletal muscle organ development	15	0,6	7,71E-02	1,00E+00	5,12E-01
GOTERM_BP_FAT	GO:0048609~reproductive process in a multicellular organism	144	5,3	7,72E-02	1,00E+00	5,11E-01
GOTERM_BP_FAT	GO:0032504~multicellular organism reproduction	144	5,3	7,72E-02	1,00E+00	5,11E-01
GOTERM_BP_FAT	GO:0016311~dephosphorylation	25	0,9	8,08E-02	1,00E+00	5,27E-01
GOTERM_BP_FAT	GO:0016049~cell growth	13	0,5	8,70E-02	1,00E+00	5,53E-01
GOTERM_BP_FAT	GO:0048285~organelle fission	33	1,2	8,89E-02	1,00E+00	5,60E-01
GOTERM_BP_FAT	GO:0009416~response to light stimulus	23	0,9	9,23E-02	1,00E+00	5,73E-01
GOTERM_BP_FAT	GO:0008105~asymmetric protein localization	10	0,4	9,60E-02	1,00E+00	5,87E-01
GOTERM_BP_FAT	GO:0008586~imaginal disc-derived wing vein morphogenesis	10	0,4	9,60E-02	1,00E+00	5,87E-01
GOTERM_BP_FAT	GO:0043085~positive regulation of catalytic activity	16	0,6	9,63E-02	1,00E+00	5,87E-01
GOTERM_BP_FAT	GO:0000226~microtubule cytoskeleton organization	59	2,2	9,69E-02	1,00E+00	5,88E-01
GOTERM_BP_FAT	GO:0006997~nucleus organization	11	0,4	9,78E-02	1,00E+00	5,90E-01
GOTERM_BP_FAT	GO:0045664~regulation of neuron differentiation	14	0,5	9,82E-02	1,00E+00	5,91E-01
GOTERM_CC_FAT	GO:0015630~microtubule cytoskeleton	75	2,8	2,78E-10	1,28E-07	1,28E-07
GOTERM_CC_FAT	GO:0005856~cytoskeleton	103	3,8	1,46E-09	6,75E-07	3,38E-07
GOTERM_CC_FAT	GO:0005886~plasma membrane	191	7,1	1,54E-09	7,12E-07	2,37E-07
GOTERM_CC_FAT	GO:0044430~cytoskeletal part	88	3,3	2,85E-09	1,31E-06	3,29E-07
GOTERM_CC_FAT	GO:0005875~microtubule associated complex	39	1,4	1,14E-05	5,24E-03	1,05E-03
GOTERM_CC_FAT	GO:0031224~intrinsic to membrane	278	10,3	7,03E-05	3,19E-02	5,39E-03
GOTERM_CC_FAT	GO:0005911~cell-cell junction	21	0,8	4,41E-04	1,84E-01	2,87E-02
GOTERM_CC_FAT	GO:0016021~integral to membrane	268	9,9	4,93E-04	2,03E-01	2,80E-02
GOTERM_CC_FAT	GO:0005815~microtubule organizing center	21	0,8	1,21E-03	4,27E-01	6,00E-02
GOTERM_CC_FAT	GO:0031012~extracellular matrix	19	0,7	1,65E-03	5,32E-01	7,32E-02
GOTERM_CC_FAT	GO:0043296~apical junction complex	16	0,6	1,71E-03	5,45E-01	6,91E-02
GOTERM_CC_FAT	GO:0016327~apicolateral plasma membrane	17	0,6	1,71E-03	5,46E-01	6,37E-02
GOTERM_CC_FAT	GO:0044459~plasma membrane part	90	3,3	2,54E-03	6,90E-01	8,61E-02
GOTERM_CC_FAT	GO:0005813~centrosome	18	0,7	3,48E-03	7,99E-01	1,08E-01

GOTERM_CC_FAT	GO:0070161~anchoring junction	17	0,6	3,64E-03	8,14E-01	1,06E-01
GOTERM_CC_FAT	GO:0045177~apical part of cell	17	0,6	3,64E-03	8,14E-01	1,06E-01
GOTERM_CC_FAT	GO:0005578~proteinaceous extracellular matrix	17	0,6	5,73E-03	9,29E-01	1,53E-01
GOTERM_CC_FAT	GO:0045179~apical cortex	10	0,4	6,19E-03	9,43E-01	1,55E-01
GOTERM_CC_FAT	GO:0030054~cell junction	35	1,3	6,48E-03	9,50E-01	1,53E-01
GOTERM_CC_FAT	GO:0005912~adherens junction	16	0,6	7,57E-03	9,70E-01	1,68E-01
GOTERM_CC_FAT	GO:0005938~cell cortex	22	0,8	9,48E-03	9,88E-01	1,97E-01
GOTERM_CC_FAT	GO:0005874~microtubule	26	1,0	1,30E-02	9,98E-01	2,50E-01
GOTERM_CC_FAT	GO:0044448~cell cortex part	15	0,6	1,51E-02	9,99E-01	2,73E-01
GOTERM_CC_FAT	GO:0005887~integral to plasma membrane	46	1,7	1,60E-02	9,99E-01	2,77E-01
GOTERM_CC_FAT	GO:0030286~dynein complex	14	0,5	2,00E-02	1,00E+00	3,21E-01
GOTERM_CC_FAT	GO:0031226~intrinsic to plasma membrane	46	1,7	2,07E-02	1,00E+00	3,20E-01
GOTERM_CC_FAT	GO:0070160~occluding junction	10	0,4	2,08E-02	1,00E+00	3,11E-01
GOTERM_CC_FAT	GO:0005819~spindle	19	0,7	2,75E-02	1,00E+00	3,78E-01
GOTERM_CC_FAT	GO:0043228~non-membrane-bounded organelle	179	6,6	2,86E-02	1,00E+00	3,80E-01
GOTERM_CC_FAT	GO:0043232~intracellular non-membrane-bounded organelle	179	6,6	2,86E-02	1,00E+00	3,80E-01
GOTERM_CC_FAT	GO:0005694~chromosome	62	2,3	6,56E-02	1,00E+00	6,60E-01
GOTERM_CC_FAT	GO:0044421~extracellular region part	33	1,2	8,66E-02	1,00E+00	7,51E-01
GOTERM_MF_FAT	GO:0043565~sequence-specific DNA binding	86	3,2	1,53E-12	1,51E-09	1,51E-09
GOTERM_MF_FAT	GO:0003704~specific RNA polymerase II transcription factor activity	41	1,5	7,56E-12	7,46E-09	3,73E-09
GOTERM_MF_FAT	GO:0003700~transcription factor activity	123	4,6	1,31E-11	1,29E-08	4,31E-09
GOTERM_MF_FAT	GO:0030528~transcription regulator activity	186	6,9	1,06E-10	1,05E-07	2,62E-08
GOTERM_MF_FAT	GO:0003702~RNA polymerase II transcription factor activity	88	3,3	1,86E-10	1,83E-07	3,66E-08
GOTERM_MF_FAT	GO:0003677~DNA binding	210	7,8	7,05E-09	6,96E-06	1,16E-06
GOTERM_MF_FAT	GO:0016564~transcription repressor activity	45	1,7	1,94E-08	1,92E-05	2,74E-06
GOTERM_MF_FAT	GO:0015631~tubulin binding	28	1,0	1,54E-05	1,51E-02	1,89E-03
GOTERM_MF_FAT	GO:0008092~cytoskeletal protein binding	64	2,4	2,60E-05	2,54E-02	2,85E-03
GOTERM_MF_FAT	GO:0008017~microtubule binding	25	0,9	9,90E-05	9,31E-02	9,72E-03
GOTERM_MF_FAT	GO:0016563~transcription activator activity	30	1,1	1,05E-03	6,46E-01	9,02E-02
GOTERM_MF_FAT	GO:0008235~metalloexopeptidase activity	17	0,6	1,87E-03	8,42E-01	1,43E-01
GOTERM_MF_FAT	GO:0016566~specific transcriptional repressor activity	15	0,6	2,19E-03	8,85E-01	1,53E-01
GOTERM_MF_FAT	GO:0004879~ligand-dependent nuclear receptor activity	11	0,4	3,60E-03	9,72E-01	2,25E-01
GOTERM_MF_FAT	GO:0004713~protein tyrosine kinase activity	21	0,8	4,79E-03	9,91E-01	2,71E-01
GOTERM_MF_FAT	GO:0008134~transcription factor binding	27	1,0	5,05E-03	9,93E-01	2,68E-01
GOTERM_MF_FAT	GO:0003714~transcription corepressor activity	10	0,4	6,39E-03	9,98E-01	3,11E-01
GOTERM_MF_FAT	GO:0046982~protein heterodimerization activity	16	0,6	1,29E-02	1,00E+00	5,10E-01
GOTERM_MF_FAT	GO:0008237~metallopeptidase activity	42	1,6	1,68E-02	1,00E+00	5,86E-01
GOTERM_MF_FAT	GO:0015294~solute:cation symporter activity	23	0,9	1,94E-02	1,00E+00	6,20E-01
GOTERM_MF_FAT	GO:0005275~amine transmembrane transporter activity	18	0,7	2,03E-02	1,00E+00	6,19E-01
GOTERM_MF_FAT	GO:0015296~anion:cation symporter activity	13	0,5	2,19E-02	1,00E+00	6,30E-01
GOTERM_MF_FAT	GO:0003712~transcription cofactor activity	19	0,7	2,24E-02	1,00E+00	6,21E-01
GOTERM_MF_FAT	GO:0016881~acid-amino acid ligase activity	30	1,1	2,37E-02	1,00E+00	6,27E-01
GOTERM_MF_FAT	GO:0004672~protein kinase activity	64	2,4	2,75E-02	1,00E+00	6,67E-01
GOTERM_MF_FAT	GO:0004842~ubiquitin-protein ligase activity	23	0,9	2,92E-02	1,00E+00	6,75E-01
GOTERM_MF_FAT	GO:0015293~symporter activity	23	0,9	3,31E-02	1,00E+00	7,08E-01
GOTERM_MF_FAT	GO:0019787~small conjugating protein ligase activity	24	0,9	3,45E-02	1,00E+00	7,10E-01
GOTERM_MF_FAT	GO:0008270~zinc ion binding	204	7,6	3,46E-02	1,00E+00	6,99E-01
GOTERM_MF_FAT	GO:0015171~amino acid transmembrane transporter activity	15	0,6	3,48E-02	1,00E+00	6,88E-01
GOTERM_MF_FAT	GO:0003729~mRNA binding	44	1,6	3,50E-02	1,00E+00	6,78E-01
GOTERM_MF_FAT	GO:0004725~protein tyrosine phosphatase activity	14	0,5	4,41E-02	1,00E+00	7,51E-01
GOTERM_MF_FAT	GO:0008238~exopeptidase activity	27	1,0	4,68E-02	1,00E+00	7,62E-01
GOTERM_MF_FAT	GO:0004674~protein serine/threonine kinase activity	46	1,7	5,90E-02	1,00E+00	8,29E-01
GOTERM_MF_FAT	GO:0004222~metalloendopeptidase activity	21	0,8	6,30E-02	1,00E+00	8,40E-01
GOTERM_MF_FAT	GO:0008083~growth factor activity	10	0,4	6,99E-02	1,00E+00	8,63E-01

Ada2a - Repression						
Category	Term	Number of affected genes	percentage	PValue	Bonferroni	Benjamini
GOTERM_BP_FAT	GO:0000902~cell morphogenesis	242	5,5	3,58E-35	1,05E-31	1,05E-31
GOTERM_BP_FAT	GO:0030182~neuron differentiation	218	4,9	2,02E-29	5,96E-26	2,98E-26
GOTERM_BP_FAT	GO:0006928~cell motion	176	4,0	3,63E-29	1,07E-25	3,56E-26
GOTERM_BP_FAT	GO:0032989~cellular component morphogenesis	258	5,8	9,51E-29	2,80E-25	7,00E-26
GOTERM_BP_FAT	GO:0045165~cell fate commitment	144	3,2	2,05E-28	6,05E-25	1,21E-25
GOTERM_BP_FAT	GO:0009886~post-embryonic morphogenesis	192	4,3	2,40E-27	7,08E-24	1,18E-24
GOTERM_BP_FAT	GO:0009791~post-embryonic development	223	5,0	3,60E-27	1,06E-23	1,52E-24
GOTERM_BP_FAT	GO:0007552~metamorphosis	195	4,4	4,58E-27	1,35E-23	1,69E-24
GOTERM_BP_FAT	GO:0048707~instar larval or pupal morphogenesis	188	4,2	1,68E-26	4,96E-23	5,51E-24
GOTERM_BP_FAT	GO:0030030~cell projection organization	194	4,4	5,71E-26	1,68E-22	1,68E-23
GOTERM_BP_FAT	GO:0002165~instar larval or pupal development	214	4,8	8,47E-26	2,49E-22	2,27E-23
GOTERM_BP_FAT	GO:0048666~neuron development	184	4,1	1,76E-24	5,18E-21	4,31E-22
GOTERM_BP_FAT	GO:0007444~imaginal disc development	207	4,7	1,24E-23	3,66E-20	2,82E-21
GOTERM_BP_FAT	GO:0000904~cell morphogenesis involved in differentiation	163	3,7	1,40E-22	4,12E-19	2,94E-20
GOTERM_BP_FAT	GO:0048569~post-embryonic organ development	159	3,6	9,36E-22	2,76E-18	1,84E-19
GOTERM_BP_FAT	GO:0048812~neuron projection morphogenesis	154	3,5	2,06E-21	6,06E-18	3,79E-19
GOTERM_BP_FAT	GO:0048858~cell projection morphogenesis	167	3,8	2,66E-21	7,83E-18	4,60E-19
GOTERM_BP_FAT	GO:0031175~neuron projection development	154	3,5	3,24E-21	9,54E-18	5,30E-19
GOTERM_BP_FAT	GO:0048667~cell morphogenesis involved in neuron differentiation	154	3,5	5,08E-21	1,50E-17	7,87E-19
GOTERM_BP_FAT	GO:0048732~gland development	102	2,3	1,25E-20	3,68E-17	1,84E-18
GOTERM_BP_FAT	GO:0007423~sensory organ development	199	4,5	1,71E-20	5,04E-17	2,40E-18
GOTERM_BP_FAT	GO:0045449~regulation of transcription	334	7,5	2,46E-20	7,24E-17	3,29E-18
GOTERM_BP_FAT	GO:0032990~cell part morphogenesis	169	3,8	2,84E-20	8,37E-17	3,64E-18
GOTERM_BP_FAT	GO:0048563~post-embryonic organ morphogenesis	150	3,4	7,99E-20	2,35E-16	9,80E-18
GOTERM_BP_FAT	GO:0007560~imaginal disc morphogenesis	150	3,4	7,99E-20	2,35E-16	9,80E-18
GOTERM_BP_FAT	GO:0048729~tissue morphogenesis	135	3,0	1,37E-19	4,04E-16	1,62E-17
GOTERM_BP_FAT	GO:0007431~salivary gland development	89	2,0	5,16E-19	1,52E-15	5,84E-17
GOTERM_BP_FAT	GO:0035272~exocrine system development	89	2,0	5,16E-19	1,52E-15	5,84E-17
GOTERM_BP_FAT	GO:0060429~epithelium development	129	2,9	5,88E-19	1,73E-15	6,41E-17
GOTERM_BP_FAT	GO:0007409~axonogenesis	113	2,5	2,19E-18	6,46E-15	2,31E-16
GOTERM_BP_FAT	GO:0006355~regulation of transcription, DNA-dependent	264	5,9	3,72E-18	1,10E-14	3,78E-16
GOTERM_BP_FAT	GO:0002009~morphogenesis of an epithelium	123	2,8	6,43E-18	1,89E-14	6,31E-16
GOTERM_BP_FAT	GO:0006350~transcription	209	4,7	1,64E-17	4,84E-14	1,56E-15
GOTERM_BP_FAT	GO:0007389~pattern specification process	216	4,9	2,28E-17	6,73E-14	2,10E-15
GOTERM_BP_FAT	GO:0007411~axon guidance	85	1,9	2,96E-17	8,72E-14	2,64E-15
GOTERM_BP_FAT	GO:0051252~regulation of RNA metabolic process	283	6,4	4,14E-17	1,22E-13	3,59E-15
GOTERM_BP_FAT	GO:0003002~regionalization	203	4,6	9,75E-16	2,94E-12	8,40E-14
GOTERM_BP_FAT	GO:0007435~salivary gland morphogenesis	73	1,6	1,23E-15	3,60E-12	9,99E-14
GOTERM_BP_FAT	GO:0022612~gland morphogenesis	73	1,6	1,23E-15	3,60E-12	9,99E-14
GOTERM_BP_FAT	GO:0051674~localization of cell	106	2,4	5,68E-15	1,67E-11	4,51E-13
GOTERM_BP_FAT	GO:0001709~cell fate determination	78	1,8	1,18E-14	3,50E-11	9,21E-13
GOTERM_BP_FAT	GO:0048870~cell motility	101	2,3	2,98E-14	8,76E-11	2,25E-12
GOTERM_BP_FAT	GO:0048736~appendage development	123	2,8	6,50E-14	1,92E-10	4,79E-12
GOTERM_BP_FAT	GO:0007163~establishment or maintenance of cell polarity	68	1,5	8,66E-14	2,55E-10	6,22E-12
GOTERM_BP_FAT	GO:0035107~appendage morphogenesis	121	2,7	1,07E-13	3,14E-10	7,47E-12
GOTERM_BP_FAT	GO:0007447~imaginal disc pattern formation	62	1,4	1,32E-13	3,88E-10	9,03E-12
GOTERM_BP_FAT	GO:0048749~compound eye development	145	3,3	1,50E-13	4,42E-10	1,00E-11
GOTERM_BP_FAT	GO:0048737~imaginal disc-derived appendage development	121	2,7	1,55E-13	4,56E-10	1,01E-11
GOTERM_BP_FAT	GO:0035114~imaginal disc-derived appendage morphogenesis	119	2,7	2,53E-13	7,46E-10	1,62E-11
GOTERM_BP_FAT	GO:0001654~eye development	153	3,4	2,64E-13	7,77E-10	1,65E-11
GOTERM_BP_FAT	GO:0030163~protein catabolic process	104	2,3	2,99E-13	8,80E-10	1,83E-11
GOTERM_BP_FAT	GO:0016477~cell migration	94	2,1	3,96E-13	1,17E-09	2,38E-11
GOTERM_BP_FAT	GO:0060541~respiratory system development	85	1,9	5,25E-13	1,55E-09	3,10E-11
GOTERM_BP_FAT	GO:0007424~open tracheal system development	85	1,9	5,25E-13	1,55E-09	3,10E-11
GOTERM_BP_FAT	GO:0035220~wing disc development	133	3,0	1,06E-12	3,12E-09	6,11E-11
GOTERM_BP_FAT	GO:0006357~regulation of transcription from RNA polymerase II promoter	99	2,2	2,30E-12	6,76E-09	1,30E-10
GOTERM_BP_FAT	GO:0007155~cell adhesion	92	2,1	2,39E-12	7,04E-09	1,33E-10
GOTERM_BP_FAT	GO:0022610~biological adhesion	96	2,2	1,06E-11	3,12E-08	5,78E-10
GOTERM_BP_FAT	GO:0035120~post-embryonic appendage morphogenesis	111	2,5	1,54E-11	4,55E-08	8,27E-10
GOTERM_BP_FAT	GO:0051603~proteolysis involved in cellular protein catabolic process	93	2,1	3,26E-11	9,60E-08	1,71E-09
GOTERM_BP_FAT	GO:0044257~cellular protein catabolic process	93	2,1	3,26E-11	9,60E-08	1,71E-09
GOTERM_BP_FAT	GO:0048598~embryonic morphogenesis	109	2,5	3,54E-11	1,04E-07	1,83E-09
GOTERM_BP_FAT	GO:0045596~negative regulation of cell differentiation	49	1,1	3,75E-11	1,10E-07	1,90E-09
GOTERM_BP_FAT	GO:0001751~compound eye photoreceptor cell differentiation	64	1,4	6,61E-11	1,95E-07	3,30E-09
GOTERM_BP_FAT	GO:0007166~cell surface receptor linked signal transduction	245	5,5	7,89E-11	2,32E-07	3,87E-09
GOTERM_BP_FAT	GO:0035218~leg disc development	37	0,8	1,69E-10	4,96E-07	8,14E-09
GOTERM_BP_FAT	GO:0043632~modification-dependent macromolecule catabolic process	86	1,9	2,64E-10	7,77E-07	1,25E-08
GOTERM_BP_FAT	GO:0012501~programmed cell death	79	1,8	4,47E-10	1,32E-06	2,09E-08
GOTERM_BP_FAT	GO:0006468~protein amino acid phosphorylation	118	2,7	4,58E-10	1,35E-06	2,11E-08
GOTERM_BP_FAT	GO:0019941~modification-dependent protein catabolic process	85	1,9	4,92E-10	1,45E-06	2,23E-08
GOTERM_BP_FAT	GO:0001738~morphogenesis of a polarized epithelium	45	1,0	5,82E-10	1,71E-06	2,60E-08
GOTERM_BP_FAT	GO:0046530~photoreceptor cell differentiation	69	1,6	6,05E-10	1,78E-06	2,66E-08
GOTERM_BP_FAT	GO:0016337~cell-cell adhesion	40	0,9	6,34E-10	1,87E-06	2,75E-08
GOTERM_BP_FAT	GO:0001754~eye photoreceptor cell differentiation	64	1,4	7,93E-10	2,34E-06	3,39E-08
GOTERM_BP_FAT	GO:0009057~macromolecule catabolic process	123	2,8	1,35E-09	3,99E-06	5,70E-08
GOTERM_BP_FAT	GO:0007219~Notch signaling pathway	39	0,9	1,55E-09	4,56E-06	6,43E-08
GOTERM_BP_FAT	GO:0048663~neuron fate commitment	37	0,8	2,05E-09	6,03E-06	8,37E-08
GOTERM_BP_FAT	GO:0006793~phosphorus metabolic process	214	4,8	2,42E-09	7,13E-06	9,76E-08
GOTERM_BP_FAT	GO:0006796~phosphate metabolic process	214	4,8	2,42E-09	7,13E-06	9,76E-08
GOTERM_BP_FAT	GO:0007472~wing disc morphogenesis	102	2,3	2,51E-09	7,39E-06	9,99E-08
GOTERM_BP_FAT	GO:0016265~death	81	1,8	2,73E-09	8,03E-06	1,07E-07
GOTERM_BP_FAT	GO:0035222~wing disc pattern formation	43	1,0	3,12E-09	9,20E-06	1,21E-07
GOTERM_BP_FAT	GO:0007476~imaginal disc-derived wing morphogenesis	101	2,3	3,18E-09	9,37E-06	1,22E-07
GOTERM_BP_FAT	GO:0007169~transmembrane receptor protein tyrosine kinase signaling pathway	60	1,4	3,25E-09	9,57E-06	1,23E-07
GOTERM_BP_FAT	GO:0051301~cell division	96	2,2	4,15E-09	1,22E-05	1,55E-07
GOTERM_BP_FAT	GO:0008219~cell death	80	1,8	4,98E-09	1,47E-05	1,83E-07
GOTERM_BP_FAT	GO:0035214~eye-antennal disc development	40	0,9	5,40E-09	1,59E-05	1,97E-07
GOTERM_BP_FAT	GO:0046552~photoreceptor cell fate commitment	35	0,8	5,78E-09	1,70E-05	2,07E-07
GOTERM_BP_FAT	GO:0007346~regulation of mitotic cell cycle	55	1,2	6,51E-09	1,92E-05	2,31E-07
GOTERM_BP_FAT	GO:0042706~eye photoreceptor cell fate commitment	33	0,7	7,37E-09	2,17E-05	2,59E-07
GOTERM_BP_FAT	GO:0001752~compound eye photoreceptor fate commitment	33	0,7	7,37E-09	2,17E-05	2,59E-07

GOTERM_BP_FAT	GO:0008356~asymmetric cell division	38	0,9	7,63E-09	2,25E-05	2,64E-07
GOTERM_BP_FAT	GO:0007167~enzyme linked receptor protein signaling pathway	73	1,6	9,08E-09	2,67E-05	3,11E-07
GOTERM_BP_FAT	GO:0001745~compound eye morphogenesis	111	2,5	9,55E-09	2,81E-05	3,23E-07
GOTERM_BP_FAT	GO:0016271~tissue death	43	1,0	1,08E-08	3,18E-05	3,62E-07
GOTERM_BP_FAT	GO:0007559~histolysis	43	1,0	1,08E-08	3,18E-05	3,62E-07
GOTERM_BP_FAT	GO:0001736~establishment of planar polarity	39	0,9	1,26E-08	3,70E-05	4,16E-07
GOTERM_BP_FAT	GO:0016331~morphogenesis of embryonic epithelium	63	1,4	1,43E-08	4,21E-05	4,68E-07
GOTERM_BP_FAT	GO:0048592~eye morphogenesis	117	2,6	1,53E-08	4,50E-05	4,95E-07
GOTERM_BP_FAT	GO:0003006~reproductive developmental process	196	4,4	1,61E-08	4,75E-05	5,16E-07
GOTERM_BP_FAT	GO:0060284~regulation of cell development	67	1,5	1,81E-08	5,34E-05	5,74E-07
GOTERM_BP_FAT	GO:0051726~regulation of cell cycle	80	1,8	1,99E-08	5,87E-05	6,24E-07
GOTERM_BP_FAT	GO:0048859~formation of anatomical boundary	31	0,7	2,08E-08	6,14E-05	6,46E-07
GOTERM_BP_FAT	GO:0001708~cell fate specification	39	0,9	2,39E-08	7,05E-05	7,34E-07
GOTERM_BP_FAT	GO:0007164~establishment of tissue polarity	39	0,9	2,39E-08	7,05E-05	7,34E-07
GOTERM_BP_FAT	GO:0044265~cellular macromolecule catabolic process	101	2,3	2,44E-08	7,17E-05	7,40E-07
GOTERM_BP_FAT	GO:0010942~positive regulation of cell death	31	0,7	4,61E-08	1,36E-04	1,39E-06
GOTERM_BP_FAT	GO:0043068~positive regulation of programmed cell death	31	0,7	4,61E-08	1,36E-04	1,39E-06
GOTERM_BP_FAT	GO:0007365~periodic partitioning	35	0,8	5,03E-08	1,48E-04	1,50E-06
GOTERM_BP_FAT	GO:0035070~salivary gland histolysis	41	0,9	5,28E-08	1,56E-04	1,56E-06
GOTERM_BP_FAT	GO:0048102~autophagic cell death	41	0,9	5,28E-08	1,56E-04	1,56E-06
GOTERM_BP_FAT	GO:0035071~salivary gland cell autophagic cell death	41	0,9	5,28E-08	1,56E-04	1,56E-06
GOTERM_BP_FAT	GO:0007391~dorsal closure	52	1,2	5,42E-08	1,60E-04	1,58E-06
GOTERM_BP_FAT	GO:0016055~Wnt receptor signaling pathway	42	0,9	7,44E-08	2,19E-04	2,15E-06
GOTERM_BP_FAT	GO:0042067~establishment of ommatidial polarity	31	0,7	9,78E-08	2,88E-04	2,80E-06
GOTERM_BP_FAT	GO:0048645~organ formation	28	0,6	1,48E-07	4,37E-04	4,20E-06
GOTERM_BP_FAT	GO:0007398~ectoderm development	40	0,9	1,99E-07	5,85E-04	5,58E-06
GOTERM_BP_FAT	GO:0008283~cell proliferation	63	1,4	2,13E-07	6,28E-04	5,92E-06
GOTERM_BP_FAT	GO:0007156~homophilic cell adhesion	22	0,5	2,24E-07	6,60E-04	6,17E-06
GOTERM_BP_FAT	GO:0009880~embryonic pattern specification	100	2,3	2,52E-07	7,42E-04	6,88E-06
GOTERM_BP_FAT	GO:0046668~regulation of retinal cell programmed cell death	16	0,4	2,81E-07	8,28E-04	7,60E-06
GOTERM_BP_FAT	GO:0007350~blastoderm segmentation	95	2,1	3,62E-07	1,07E-03	9,70E-06
GOTERM_BP_FAT	GO:0007455~eye-antennal disc morphogenesis	27	0,6	3,70E-07	1,09E-03	9,81E-06
GOTERM_BP_FAT	GO:0007548~sex differentiation	36	0,8	4,79E-07	1,41E-03	1,26E-05
GOTERM_BP_FAT	GO:0007449~proximal/distal pattern formation, imaginal disc	18	0,4	5,08E-07	1,49E-03	1,32E-05
GOTERM_BP_FAT	GO:0043067~regulation of programmed cell death	56	1,3	5,59E-07	1,64E-03	1,44E-05
GOTERM_BP_FAT	GO:0009954~proximal/distal pattern formation	19	0,4	5,77E-07	1,70E-03	1,48E-05
GOTERM_BP_FAT	GO:0051276~chromosome organization	116	2,6	6,38E-07	1,88E-03	1,62E-05
GOTERM_BP_FAT	GO:0007422~peripheral nervous system development	47	1,1	6,75E-07	1,99E-03	1,70E-05
GOTERM_BP_FAT	GO:0048813~dendrite morphogenesis	58	1,3	7,14E-07	2,10E-03	1,78E-05
GOTERM_BP_FAT	GO:0016358~dendrite development	58	1,3	7,14E-07	2,10E-03	1,78E-05
GOTERM_BP_FAT	GO:0008038~neuron recognition	31	0,7	7,48E-07	2,20E-03	1,85E-05
GOTERM_BP_FAT	GO:0060173~limb development	27	0,6	7,81E-07	2,30E-03	1,92E-05
GOTERM_BP_FAT	GO:0035108~limb morphogenesis	27	0,6	7,81E-07	2,30E-03	1,92E-05
GOTERM_BP_FAT	GO:0000122~negative regulation of transcription from RNA polymerase II promoter	36	0,8	8,28E-07	2,43E-03	2,01E-05
GOTERM_BP_FAT	GO:0030707~ovarian follicle cell development	89	2,0	8,36E-07	2,46E-03	2,02E-05
GOTERM_BP_FAT	GO:0010160~formation of organ boundary	26	0,6	9,10E-07	2,68E-03	2,18E-05
GOTERM_BP_FAT	GO:0035282~segmentation	108	2,4	9,35E-07	2,75E-03	2,22E-05
GOTERM_BP_FAT	GO:0009968~negative regulation of signal transduction	52	1,2	1,01E-06	2,97E-03	2,38E-05
GOTERM_BP_FAT	GO:0010564~regulation of cell cycle process	45	1,0	1,09E-06	3,21E-03	2,55E-05
GOTERM_BP_FAT	GO:0007450~dorsal/ventral pattern formation, imaginal disc	34	0,8	1,29E-06	3,79E-03	2,99E-05
GOTERM_BP_FAT	GO:0008037~cell recognition	31	0,7	1,38E-06	4,06E-03	3,18E-05
GOTERM_BP_FAT	GO:0022604~regulation of cell morphogenesis	58	1,3	1,43E-06	4,22E-03	3,27E-05
GOTERM_BP_FAT	GO:0050767~regulation of neurogenesis	38	0,9	1,46E-06	4,30E-03	3,31E-05
GOTERM_BP_FAT	GO:0010648~negative regulation of cell communication	52	1,2	1,47E-06	4,33E-03	3,31E-05
GOTERM_BP_FAT	GO:0051254~positive regulation of RNA metabolic process	47	1,1	1,55E-06	4,55E-03	3,46E-05
GOTERM_BP_FAT	GO:0051960~regulation of nervous system development	47	1,1	1,55E-06	4,55E-03	3,46E-05
GOTERM_BP_FAT	GO:0035215~genital disc development	21	0,5	1,61E-06	4,72E-03	3,55E-05
GOTERM_BP_FAT	GO:0014016~neuroblast differentiation	21	0,5	1,61E-06	4,72E-03	3,55E-05
GOTERM_BP_FAT	GO:0008045~motor axon guidance	18	0,4	1,66E-06	4,88E-03	3,65E-05
GOTERM_BP_FAT	GO:0008544~epidermis development	30	0,7	1,69E-06	4,97E-03	3,69E-05
GOTERM_BP_FAT	GO:0048589~developmental growth	43	1,0	1,77E-06	5,19E-03	3,83E-05
GOTERM_BP_FAT	GO:0007367~segment polarity determination	26	0,6	1,88E-06	5,51E-03	4,03E-05
GOTERM_BP_FAT	GO:0035110~leg morphogenesis	26	0,6	1,88E-06	5,51E-03	4,03E-05
GOTERM_BP_FAT	GO:0045893~positive regulation of transcription, DNA-dependent	46	1,0	1,97E-06	5,80E-03	4,21E-05
GOTERM_BP_FAT	GO:0007242~intracellular signaling cascade	119	2,7	2,19E-06	6,44E-03	4,65E-05
GOTERM_BP_FAT	GO:0010941~regulation of cell death	56	1,3	2,32E-06	6,81E-03	4,88E-05
GOTERM_BP_FAT	GO:0048565~gut development	40	0,9	2,33E-06	6,84E-03	4,87E-05
GOTERM_BP_FAT	GO:0001700~embryonic development via the syncytial blastoderm	91	2,1	2,34E-06	6,87E-03	4,85E-05
GOTERM_BP_FAT	GO:0035295~tube development	50	1,1	2,39E-06	7,02E-03	4,93E-05
GOTERM_BP_FAT	GO:0009967~positive regulation of signal transduction	31	0,7	2,48E-06	7,26E-03	5,06E-05
GOTERM_BP_FAT	GO:0007088~regulation of mitosis	27	0,6	3,06E-06	8,98E-03	6,22E-05
GOTERM_BP_FAT	GO:0051783~regulation of nuclear division	27	0,6	3,06E-06	8,98E-03	6,22E-05
GOTERM_BP_FAT	GO:0007478~leg disc morphogenesis	22	0,5	3,41E-06	1,00E-02	6,88E-05
GOTERM_BP_FAT	GO:0007507~heart development	38	0,9	3,76E-06	1,10E-02	7,53E-05
GOTERM_BP_FAT	GO:0014017~neuroblast fate commitment	20	0,5	4,23E-06	1,24E-02	8,41E-05
GOTERM_BP_FAT	GO:0010628~positive regulation of gene expression	56	1,3	4,51E-06	1,32E-02	8,92E-05
GOTERM_BP_FAT	GO:0016310~phosphorylation	159	3,6	4,95E-06	1,45E-02	9,72E-05
GOTERM_BP_FAT	GO:0048190~wing disc dorsal/ventral pattern formation	30	0,7	5,39E-06	1,58E-02	1,05E-04
GOTERM_BP_FAT	GO:0051173~positive regulation of nitrogen compound metabolic process	57	1,3	6,71E-06	1,96E-02	1,30E-04
GOTERM_BP_FAT	GO:0045935~positive regulation of nucleobase, nucleoside, nucleotide and nucleic acid metabolic process	57	1,3	6,71E-06	1,96E-02	1,30E-04
GOTERM_BP_FAT	GO:0042063~gliogenesis	29	0,7	6,72E-06	1,96E-02	1,29E-04
GOTERM_BP_FAT	GO:0010647~positive regulation of cell communication	31	0,7	7,35E-06	2,14E-02	1,41E-04
GOTERM_BP_FAT	GO:0045941~positive regulation of transcription	55	1,2	7,89E-06	2,30E-02	1,50E-04
GOTERM_BP_FAT	GO:0045466~R7 cell differentiation	21	0,5	8,53E-06	2,48E-02	1,61E-04
GOTERM_BP_FAT	GO:0035109~imaginal disc-derived limb morphogenesis	21	0,5	8,53E-06	2,48E-02	1,61E-04
GOTERM_BP_FAT	GO:0010604~positive regulation of macromolecule metabolic process	64	1,4	1,02E-05	2,95E-02	1,91E-04
GOTERM_BP_FAT	GO:0009792~embryonic development ending in birth or egg hatching	92	2,1	1,13E-05	3,28E-02	2,11E-04
GOTERM_BP_FAT	GO:0008360~regulation of cell shape	48	1,1	1,13E-05	3,28E-02	2,10E-04
GOTERM_BP_FAT	GO:0006470~protein amino acid dephosphorylation	42	0,9	1,15E-05	3,32E-02	2,11E-04
GOTERM_BP_FAT	GO:0035223~leg disc pattern formation	14	0,3	1,16E-05	3,36E-02	2,12E-04
GOTERM_BP_FAT	GO:0010454~negative regulation of cell fate commitment	14	0,3	1,16E-05	3,36E-02	2,12E-04
GOTERM_BP_FAT	GO:0009996~negative regulation of cell fate specification	14	0,3	1,16E-05	3,36E-02	2,12E-04
GOTERM_BP_FAT	GO:0007479~leg disc proximal/distal pattern formation	14	0,3	1,16E-05	3,36E-02	2,12E-04
GOTERM_BP_FAT	GO:0010453~regulation of cell fate commitment	16	0,4	1,32E-05	3,82E-02	2,41E-04
GOTERM_BP_FAT	GO:0042659~regulation of cell fate specification	16	0,4	1,32E-05	3,82E-02	2,41E-04

GOTERM_BP_FAT	GO:0007018~microtubule-based movement	50	1,1	1,37E-05	3,95E-02	2,47E-04
GOTERM_BP_FAT	GO:0006511~ubiquitin-dependent protein catabolic process	47	1,1	1,44E-05	4,16E-02	2,59E-04
GOTERM_BP_FAT	GO:0007498~mesoderm development	45	1,0	1,66E-05	4,76E-02	2,95E-04
GOTERM_BP_FAT	GO:0048610~reproductive cellular process	180	4,1	1,67E-05	4,80E-02	2,96E-04
GOTERM_BP_FAT	GO:0007419~ventral cord development	21	0,5	1,78E-05	5,12E-02	3,15E-04
GOTERM_BP_FAT	GO:0031328~positive regulation of cellular biosynthetic process	67	1,5	1,94E-05	5,55E-02	3,40E-04
GOTERM_BP_FAT	GO:0009891~positive regulation of biosynthetic process	67	1,5	1,94E-05	5,55E-02	3,40E-04
GOTERM_BP_FAT	GO:0045944~positive regulation of transcription from RNA polymerase II promoter	29	0,7	1,95E-05	5,57E-02	3,39E-04
GOTERM_BP_FAT	GO:0007480~imaginal disc-derived leg morphogenesis	20	0,5	2,10E-05	6,00E-02	3,64E-04
GOTERM_BP_FAT	GO:0035127~post-embryonic limb morphogenesis	20	0,5	2,10E-05	6,00E-02	3,64E-04
GOTERM_BP_FAT	GO:0035239~tube morphogenesis	42	0,9	2,40E-05	6,83E-02	4,14E-04
GOTERM_BP_FAT	GO:0043297~apical junction assembly	19	0,4	2,45E-05	6,95E-02	4,19E-04
GOTERM_BP_FAT	GO:0007400~neuroblast fate determination	18	0,4	2,80E-05	7,92E-02	4,77E-04
GOTERM_BP_FAT	GO:0048864~stem cell development	22	0,5	2,91E-05	8,22E-02	4,93E-04
GOTERM_BP_FAT	GO:0012502~induction of programmed cell death	22	0,5	2,91E-05	8,22E-02	4,93E-04
GOTERM_BP_FAT	GO:0007405~neuroblast proliferation	22	0,5	2,91E-05	8,22E-02	4,93E-04
GOTERM_BP_FAT	GO:0048863~stem cell differentiation	22	0,5	2,91E-05	8,22E-02	4,93E-04
GOTERM_BP_FAT	GO:0046669~regulation of compound eye retinal cell programmed cell death	12	0,3	2,91E-05	8,22E-02	4,90E-04
GOTERM_BP_FAT	GO:0010557~positive regulation of macromolecule biosynthetic process	58	1,3	3,06E-05	8,63E-02	5,13E-04
GOTERM_BP_FAT	GO:0017145~stem cell division	29	0,7	3,19E-05	8,97E-02	5,31E-04
GOTERM_BP_FAT	GO:0007017~microtubule-based process	149	3,4	3,26E-05	9,15E-02	5,39E-04
GOTERM_BP_FAT	GO:0045664~regulation of neuron differentiation	30	0,7	4,02E-05	1,12E-01	6,62E-04
GOTERM_BP_FAT	GO:0034329~cell junction assembly	20	0,5	4,26E-05	1,18E-01	6,96E-04
GOTERM_BP_FAT	GO:0008407~bristle morphogenesis	27	0,6	5,12E-05	1,40E-01	8,33E-04
GOTERM_BP_FAT	GO:0043065~positive regulation of apoptosis	22	0,5	5,40E-05	1,47E-01	8,74E-04
GOTERM_BP_FAT	GO:0007059~chromosome segregation	53	1,2	5,58E-05	1,51E-01	8,97E-04
GOTERM_BP_FAT	GO:0022416~bristle development	30	0,7	6,29E-05	1,69E-01	1,01E-03
GOTERM_BP_FAT	GO:0019827~stem cell maintenance	21	0,5	6,66E-05	1,78E-01	1,06E-03
GOTERM_BP_FAT	GO:0007096~regulation of exit from mitosis	10	0,2	6,68E-05	1,79E-01	1,06E-03
GOTERM_BP_FAT	GO:0006325~chromatin organization	71	1,6	6,89E-05	1,84E-01	1,08E-03
GOTERM_BP_FAT	GO:0009953~dorsal/ventral pattern formation	59	1,3	6,99E-05	1,86E-01	1,09E-03
GOTERM_BP_FAT	GO:0007298~border follicle cell migration	34	0,8	8,06E-05	2,11E-01	1,26E-03
GOTERM_BP_FAT	GO:0055123~digestive system development	27	0,6	8,27E-05	2,16E-01	1,28E-03
GOTERM_BP_FAT	GO:0048546~digestive tract morphogenesis	27	0,6	8,27E-05	2,16E-01	1,28E-03
GOTERM_BP_FAT	GO:0009913~epidermal cell differentiation	22	0,5	9,62E-05	2,47E-01	1,48E-03
GOTERM_BP_FAT	GO:0045137~development of primary sexual characteristics	22	0,5	9,62E-05	2,47E-01	1,48E-03
GOTERM_BP_FAT	GO:0007427~epithelial cell migration, open tracheal system	19	0,4	9,98E-05	2,55E-01	1,53E-03
GOTERM_BP_FAT	GO:0048103~somatic stem cell division	19	0,4	9,98E-05	2,55E-01	1,53E-03
GOTERM_BP_FAT	GO:0007043~cell-cell junction assembly	19	0,4	9,98E-05	2,55E-01	1,53E-03
GOTERM_BP_FAT	GO:0010631~epithelial cell migration	19	0,4	9,98E-05	2,55E-01	1,53E-03
GOTERM_BP_FAT	GO:0007276~gamete generation	241	5,4	1,05E-04	2,65E-01	1,60E-03
GOTERM_BP_FAT	GO:0007439~ectodermal gut development	26	0,6	1,05E-04	2,66E-01	1,59E-03
GOTERM_BP_FAT	GO:0048567~ectodermal gut morphogenesis	26	0,6	1,05E-04	2,66E-01	1,59E-03
GOTERM_BP_FAT	GO:0042981~regulation of apoptosis	45	1,0	1,14E-04	2,86E-01	1,73E-03
GOTERM_BP_FAT	GO:0007517~muscle organ development	60	1,4	1,17E-04	2,92E-01	1,76E-03
GOTERM_BP_FAT	GO:0035316~non-sensory hair organization	21	0,5	1,20E-04	2,98E-01	1,79E-03
GOTERM_BP_FAT	GO:0035315~hair cell differentiation	21	0,5	1,20E-04	2,98E-01	1,79E-03
GOTERM_BP_FAT	GO:0007297~ovarian follicle cell migration	38	0,9	1,27E-04	3,12E-01	1,89E-03
GOTERM_BP_FAT	GO:0006508~proteolysis	245	5,5	1,37E-04	3,31E-01	2,02E-03
GOTERM_BP_FAT	GO:0016311~dephosphorylation	49	1,1	1,40E-04	3,38E-01	2,06E-03
GOTERM_BP_FAT	GO:0033043~regulation of organelle organization	49	1,1	1,40E-04	3,38E-01	2,06E-03
GOTERM_BP_FAT	GO:0048534~hemopoietic or lymphoid organ development	30	0,7	1,45E-04	3,48E-01	2,13E-03
GOTERM_BP_FAT	GO:0002520~immune system development	30	0,7	1,45E-04	3,48E-01	2,13E-03
GOTERM_BP_FAT	GO:0008593~regulation of Notch signaling pathway	17	0,4	1,46E-04	3,49E-01	2,12E-03
GOTERM_BP_FAT	GO:0001667~ameboidal cell migration	20	0,5	1,50E-04	3,56E-01	2,17E-03
GOTERM_BP_FAT	GO:0048547~gut morphogenesis	26	0,6	1,66E-04	3,88E-01	2,40E-03
GOTERM_BP_FAT	GO:0008052~sensory organ boundary specification	16	0,4	1,74E-04	4,00E-01	2,49E-03
GOTERM_BP_FAT	GO:0007469~antennal development	16	0,4	1,74E-04	4,00E-01	2,49E-03
GOTERM_BP_FAT	GO:0046578~regulation of Ras protein signal transduction	38	0,9	1,76E-04	4,05E-01	2,52E-03
GOTERM_BP_FAT	GO:0040007~growth	49	1,1	1,83E-04	4,17E-01	2,60E-03
GOTERM_BP_FAT	GO:0007293~germarium-derived egg chamber formation	32	0,7	1,92E-04	4,31E-01	2,71E-03
GOTERM_BP_FAT	GO:0042461~photoreceptor cell development	32	0,7	1,92E-04	4,31E-01	2,71E-03
GOTERM_BP_FAT	GO:0019953~sexual reproduction	245	5,5	2,01E-04	4,47E-01	2,83E-03
GOTERM_BP_FAT	GO:0048568~embryonic organ development	15	0,3	2,04E-04	4,51E-01	2,86E-03
GOTERM_BP_FAT	GO:0046532~regulation of photoreceptor cell differentiation	15	0,3	2,04E-04	4,51E-01	2,86E-03
GOTERM_BP_FAT	GO:0007010~cytoskeleton organization	163	3,7	2,08E-04	4,57E-01	2,89E-03
GOTERM_BP_FAT	GO:0035162~embryonic hemopoiesis	14	0,3	2,35E-04	5,00E-01	3,26E-03
GOTERM_BP_FAT	GO:0035277~spiracle morphogenesis, open tracheal system	14	0,3	2,35E-04	5,00E-01	3,26E-03
GOTERM_BP_FAT	GO:0035317~imaginal disc-derived wing hair organization	20	0,5	2,62E-04	5,38E-01	3,62E-03
GOTERM_BP_FAT	GO:0030097~hemopoiesis	24	0,5	2,71E-04	5,50E-01	3,72E-03
GOTERM_BP_FAT	GO:0035088~establishment or maintenance of apical/basal cell polarity	17	0,4	2,82E-04	5,65E-01	3,86E-03
GOTERM_BP_FAT	GO:0030111~regulation of Wnt receptor signaling pathway	19	0,4	3,30E-04	6,21E-01	4,48E-03
GOTERM_BP_FAT	GO:0030855~epithelial cell differentiation	16	0,4	3,45E-04	6,38E-01	4,68E-03
GOTERM_BP_FAT	GO:0030031~cell projection assembly	32	0,7	3,89E-04	6,82E-01	5,24E-03
GOTERM_BP_FAT	GO:0008258~head involution	26	0,6	3,90E-04	6,83E-01	5,23E-03
GOTERM_BP_FAT	GO:0055057~neuroblast division	15	0,3	4,19E-04	7,09E-01	5,60E-03
GOTERM_BP_FAT	GO:0055059~asymmetric neuroblast division	15	0,3	4,19E-04	7,09E-01	5,60E-03
GOTERM_BP_FAT	GO:0060560~developmental growth involved in morphogenesis	14	0,3	5,03E-04	7,73E-01	6,68E-03
GOTERM_BP_FAT	GO:0016198~axon choice point recognition	14	0,3	5,03E-04	7,73E-01	6,68E-03
GOTERM_BP_FAT	GO:0030178~negative regulation of Wnt receptor signaling pathway	14	0,3	5,03E-04	7,73E-01	6,68E-03
GOTERM_BP_FAT	GO:0035051~cardiac cell differentiation	14	0,3	5,03E-04	7,73E-01	6,68E-03
GOTERM_BP_FAT	GO:0045448~mitotic cell cycle, embryonic	17	0,4	5,17E-04	7,82E-01	6,84E-03
GOTERM_BP_FAT	GO:0007611~learning or memory	36	0,8	5,29E-04	7,90E-01	6,97E-03
GOTERM_BP_FAT	GO:0051056~regulation of small GTPase mediated signal transduction	42	0,9	5,33E-04	7,92E-01	6,98E-03
GOTERM_BP_FAT	GO:0016567~protein ubiquitination	23	0,5	5,39E-04	7,96E-01	7,04E-03
GOTERM_BP_FAT	GO:0007442~hindgut morphogenesis	23	0,5	5,39E-04	7,96E-01	7,04E-03
GOTERM_BP_FAT	GO:0030010~establishment of cell polarity	13	0,3	5,95E-04	8,27E-01	7,73E-03
GOTERM_BP_FAT	GO:0007369~gastrulation	34	0,8	6,30E-04	8,44E-01	8,15E-03
GOTERM_BP_FAT	GO:0035017~cuticle pattern formation	16	0,4	6,45E-04	8,50E-01	8,30E-03
GOTERM_BP_FAT	GO:0016318~ommatidial rotation	16	0,4	6,45E-04	8,50E-01	8,30E-03
GOTERM_BP_FAT	GO:0042051~compound eye photoreceptor development	27	0,6	6,57E-04	8,56E-01	8,41E-03
GOTERM_BP_FAT	GO:0000087~M phase of mitotic cell cycle	60	1,4	6,76E-04	8,63E-01	8,62E-03
GOTERM_BP_FAT	GO:0032446~protein modification by small protein conjugation	25	0,6	7,41E-04	8,87E-01	9,41E-03
GOTERM_BP_FAT	GO:0070647~protein modification by small protein conjugation or removal	32	0,7	7,48E-04	8,89E-01	9,45E-03
GOTERM_BP_FAT	GO:0046529~imaginal disc fusion, thorax closure	11	0,2	7,77E-04	8,99E-01	9,78E-03

GOTERM_BP_FAT	GO:0045167~asymmetric protein localization involved in cell fate determination	11	0,2	7,77E-04	8,99E-01	9,78E-03
GOTERM_BP_FAT	GO:0016481~negative regulation of transcription	79	1,8	7,88E-04	9,02E-01	9,87E-03
GOTERM_BP_FAT	GO:0007067~mitosis	59	1,3	8,27E-04	9,13E-01	1,03E-02
GOTERM_BP_FAT	GO:0035111~leg joint morphogenesis	10	0,2	8,36E-04	9,15E-01	1,04E-02
GOTERM_BP_FAT	GO:0051172~negative regulation of nitrogen compound metabolic process	84	1,9	8,43E-04	9,17E-01	1,04E-02
GOTERM_BP_FAT	GO:0045934~negative regulation of nucleobase, nucleoside, nucleotide and nucleic acid metabolic process	84	1,9	8,43E-04	9,17E-01	1,04E-02
GOTERM_BP_FAT	GO:0035152~regulation of tube architecture, open tracheal system	21	0,5	8,85E-04	9,26E-01	1,09E-02
GOTERM_BP_FAT	GO:0016333~morphogenesis of follicular epithelium	17	0,4	9,01E-04	9,30E-01	1,10E-02
GOTERM_BP_FAT	GO:0008293~torso signaling pathway	19	0,4	9,19E-04	9,33E-01	1,12E-02
GOTERM_BP_FAT	GO:0042462~eye photoreceptor cell development	27	0,6	9,39E-04	9,37E-01	1,14E-02
GOTERM_BP_FAT	GO:0009890~negative regulation of biosynthetic process	92	2,1	9,61E-04	9,41E-01	1,16E-02
GOTERM_BP_FAT	GO:0031327~negative regulation of cellular biosynthetic process	92	2,1	9,61E-04	9,41E-01	1,16E-02
GOTERM_BP_FAT	GO:0010558~negative regulation of macromolecule biosynthetic process	91	2,1	9,83E-04	9,45E-01	1,19E-02
GOTERM_BP_FAT	GO:0010001~glial cell differentiation	14	0,3	9,89E-04	9,46E-01	1,19E-02
GOTERM_BP_FAT	GO:0034330~cell junction organization	20	0,5	1,14E-03	9,65E-01	1,36E-02
GOTERM_BP_FAT	GO:0006917~induction of apoptosis	16	0,4	1,14E-03	9,65E-01	1,36E-02
GOTERM_BP_FAT	GO:0030718~germ-line stem cell maintenance	16	0,4	1,14E-03	9,65E-01	1,36E-02
GOTERM_BP_FAT	GO:0051130~positive regulation of cellular component organization	16	0,4	1,14E-03	9,65E-01	1,36E-02
GOTERM_BP_FAT	GO:0040008~regulation of growth	44	1,0	1,18E-03	9,69E-01	1,40E-02
GOTERM_BP_FAT	GO:0016360~sensory organ precursor cell fate determination	13	0,3	1,21E-03	9,72E-01	1,43E-02
GOTERM_BP_FAT	GO:0007280~pole cell migration	13	0,3	1,21E-03	9,72E-01	1,43E-02
GOTERM_BP_FAT	GO:0000280~nuclear division	59	1,3	1,22E-03	9,73E-01	1,43E-02
GOTERM_BP_FAT	GO:0060446~branching involved in open tracheal system development	21	0,5	1,35E-03	9,81E-01	1,58E-02
GOTERM_BP_FAT	GO:0048754~branching morphogenesis of a tube	21	0,5	1,35E-03	9,81E-01	1,58E-02
GOTERM_BP_FAT	GO:0043062~extracellular structure organization	32	0,7	1,37E-03	9,82E-01	1,59E-02
GOTERM_BP_FAT	GO:0051094~positive regulation of developmental process	15	0,3	1,44E-03	9,86E-01	1,67E-02
GOTERM_BP_FAT	GO:0000278~mitotic cell cycle	125	2,8	1,45E-03	9,86E-01	1,67E-02
GOTERM_BP_FAT	GO:0035285~appendage segmentation	12	0,3	1,47E-03	9,87E-01	1,69E-02
GOTERM_BP_FAT	GO:0035286~leg segmentation	12	0,3	1,47E-03	9,87E-01	1,69E-02
GOTERM_BP_FAT	GO:0019991~septate junction assembly	12	0,3	1,47E-03	9,87E-01	1,69E-02
GOTERM_BP_FAT	GO:0016319~mushroom body development	25	0,6	1,54E-03	9,89E-01	1,76E-02
GOTERM_BP_FAT	GO:0060562~epithelial tube morphogenesis	22	0,5	1,57E-03	9,90E-01	1,79E-02
GOTERM_BP_FAT	GO:0001763~morphogenesis of a branching structure	22	0,5	1,57E-03	9,90E-01	1,79E-02
GOTERM_BP_FAT	GO:0008104~protein localization	135	3,0	1,69E-03	9,93E-01	1,92E-02
GOTERM_BP_FAT	GO:0033301~cell cycle comprising mitosis without cytokinesis	11	0,2	1,76E-03	9,94E-01	1,99E-02
GOTERM_BP_FAT	GO:0046528~imaginal disc fusion	11	0,2	1,76E-03	9,94E-01	1,99E-02
GOTERM_BP_FAT	GO:0045570~regulation of imaginal disc growth	11	0,2	1,76E-03	9,94E-01	1,99E-02
GOTERM_BP_FAT	GO:0007428~primary branching, open tracheal system	11	0,2	1,76E-03	9,94E-01	1,99E-02
GOTERM_BP_FAT	GO:0007379~segment specification	23	0,5	1,77E-03	9,95E-01	2,00E-02
GOTERM_BP_FAT	GO:0046620~regulation of organ growth	18	0,4	1,87E-03	9,96E-01	2,10E-02
GOTERM_BP_FAT	GO:0048609~reproductive process in a multicellular organism	250	5,6	1,89E-03	9,96E-01	2,12E-02
GOTERM_BP_FAT	GO:0032504~multicellular organism reproduction	250	5,6	1,89E-03	9,96E-01	2,12E-02
GOTERM_BP_FAT	GO:0048332~mesoderm morphogenesis	16	0,4	1,92E-03	9,96E-01	2,13E-02
GOTERM_BP_FAT	GO:0010002~cardioblast differentiation	10	0,2	2,05E-03	9,98E-01	2,28E-02
GOTERM_BP_FAT	GO:0007440~foregut morphogenesis	10	0,2	2,05E-03	9,98E-01	2,28E-02
GOTERM_BP_FAT	GO:0045216~cell-cell junction organization	19	0,4	2,24E-03	9,99E-01	2,47E-02
GOTERM_BP_FAT	GO:0010721~negative regulation of cell development	13	0,3	2,28E-03	9,99E-01	2,50E-02
GOTERM_BP_FAT	GO:0007465~R7 cell fate commitment	13	0,3	2,28E-03	9,99E-01	2,50E-02
GOTERM_BP_FAT	GO:0009408~response to heat	31	0,7	2,30E-03	9,99E-01	2,52E-02
GOTERM_BP_FAT	GO:0002164~larval development	31	0,7	2,30E-03	9,99E-01	2,52E-02
GOTERM_BP_FAT	GO:0008406~gonad development	17	0,4	2,40E-03	9,99E-01	2,62E-02
GOTERM_BP_FAT	GO:0042306~regulation of protein import into nucleus	17	0,4	2,40E-03	9,99E-01	2,62E-02
GOTERM_BP_FAT	GO:0048608~reproductive structure development	17	0,4	2,40E-03	9,99E-01	2,62E-02
GOTERM_BP_FAT	GO:0007283~spermatogenesis	60	1,4	2,49E-03	9,99E-01	2,70E-02
GOTERM_BP_FAT	GO:0048232~male gamete generation	60	1,4	2,49E-03	9,99E-01	2,70E-02
GOTERM_BP_FAT	GO:0006915~apoptosis	34	0,8	2,55E-03	9,99E-01	2,75E-02
GOTERM_BP_FAT	GO:0007292~female gamete generation	180	4,1	2,71E-03	1,00E+00	2,92E-02
GOTERM_BP_FAT	GO:0007143~female meiosis	29	0,7	2,78E-03	1,00E+00	2,98E-02
GOTERM_BP_FAT	GO:0007224~smoothened signaling pathway	18	0,4	2,88E-03	1,00E+00	3,07E-02
GOTERM_BP_FAT	GO:0048285~organelle fission	59	1,3	3,00E-03	1,00E+00	3,19E-02
GOTERM_BP_FAT	GO:0007494~midgut development	16	0,4	3,09E-03	1,00E+00	3,27E-02
GOTERM_BP_FAT	GO:0042058~regulation of epidermal growth factor receptor signaling pathway	14	0,3	3,13E-03	1,00E+00	3,30E-02
GOTERM_BP_FAT	GO:0030720~oocyte localization during germarium-derived egg chamber formation	11	0,2	3,54E-03	1,00E+00	3,71E-02
GOTERM_BP_FAT	GO:0021782~glial cell development	11	0,2	3,54E-03	1,00E+00	3,71E-02
GOTERM_BP_FAT	GO:0000819~sister chromatid segregation	23	0,5	3,54E-03	1,00E+00	3,70E-02
GOTERM_BP_FAT	GO:0010605~negative regulation of macromolecule metabolic process	110	2,5	3,56E-03	1,00E+00	3,71E-02
GOTERM_BP_FAT	GO:0033157~regulation of intracellular protein transport	17	0,4	3,71E-03	1,00E+00	3,85E-02
GOTERM_BP_FAT	GO:0048511~rhythmic process	24	0,5	3,81E-03	1,00E+00	3,93E-02
GOTERM_BP_FAT	GO:0001707~mesoderm formation	15	0,3	3,98E-03	1,00E+00	4,09E-02
GOTERM_BP_FAT	GO:0007426~tracheal outgrowth, open tracheal system	13	0,3	3,99E-03	1,00E+00	4,09E-02
GOTERM_BP_FAT	GO:0035287~head segmentation	13	0,3	3,99E-03	1,00E+00	4,09E-02
GOTERM_BP_FAT	GO:0050808~synapse organization	21	0,5	4,17E-03	1,00E+00	4,25E-02
GOTERM_BP_FAT	GO:0007264~small GTPase mediated signal transduction	44	1,0	4,29E-03	1,00E+00	4,36E-02
GOTERM_BP_FAT	GO:0008105~asymmetric protein localization	18	0,4	4,30E-03	1,00E+00	4,36E-02
GOTERM_BP_FAT	GO:0035186~syncytial blastoderm mitotic cell cycle	10	0,2	4,35E-03	1,00E+00	4,39E-02
GOTERM_BP_FAT	GO:0048854~brain morphogenesis	10	0,2	4,35E-03	1,00E+00	4,39E-02
GOTERM_BP_FAT	GO:0007539~primary sex determination, soma	10	0,2	4,35E-03	1,00E+00	4,39E-02
GOTERM_BP_FAT	GO:0035099~hemocyte migration	10	0,2	4,35E-03	1,00E+00	4,39E-02
GOTERM_BP_FAT	GO:0001964~startle response	10	0,2	4,35E-03	1,00E+00	4,39E-02
GOTERM_BP_FAT	GO:0031987~locomotion involved in locomotory behavior	10	0,2	4,35E-03	1,00E+00	4,39E-02
GOTERM_BP_FAT	GO:0010623~developmental programmed cell death	10	0,2	4,35E-03	1,00E+00	4,39E-02
GOTERM_BP_FAT	GO:0051253~negative regulation of RNA metabolic process	71	1,6	4,36E-03	1,00E+00	4,39E-02
GOTERM_BP_FAT	GO:0048477~oogenesis	176	4,0	4,71E-03	1,00E+00	4,71E-02
GOTERM_BP_FAT	GO:0016049~cell growth	23	0,5	4,86E-03	1,00E+00	4,85E-02
GOTERM_BP_FAT	GO:0051336~regulation of hydrolase activity	31	0,7	5,03E-03	1,00E+00	4,99E-02
GOTERM_BP_FAT	GO:0050768~negative regulation of neurogenesis	12	0,3	5,09E-03	1,00E+00	5,04E-02
GOTERM_BP_FAT	GO:0046660~female sex differentiation	12	0,3	5,09E-03	1,00E+00	5,04E-02
GOTERM_BP_FAT	GO:0051017~actin filament bundle formation	12	0,3	5,09E-03	1,00E+00	5,04E-02
GOTERM_BP_FAT	GO:0042692~muscle cell differentiation	32	0,7	5,12E-03	1,00E+00	5,04E-02
GOTERM_BP_FAT	GO:0048638~regulation of developmental growth	20	0,5	5,36E-03	1,00E+00	5,25E-02
GOTERM_BP_FAT	GO:0030162~regulation of proteolysis	20	0,5	5,36E-03	1,00E+00	5,25E-02
GOTERM_BP_FAT	GO:0060538~skeletal muscle organ development	26	0,6	5,66E-03	1,00E+00	5,52E-02
GOTERM_BP_FAT	GO:0051297~centrosome organization	21	0,5	5,81E-03	1,00E+00	5,65E-02
GOTERM_BP_FAT	GO:0007243~protein kinase cascade	27	0,6	5,86E-03	1,00E+00	5,68E-02
GOTERM_BP_FAT	GO:0000070~mitotic sister chromatid segregation	22	0,5	6,22E-03	1,00E+00	6,00E-02

GOTERM_BP_FAT	GO:0051656~establishment of organelle localization	32	0,7	6,47E-03	1,00E+00	6,21E-02
GOTERM_BP_FAT	GO:0048871~multicellular organismal homeostasis	11	0,2	6,49E-03	1,00E+00	6,21E-02
GOTERM_BP_FAT	GO:0035290~trunk segmentation	11	0,2	6,49E-03	1,00E+00	6,21E-02
GOTERM_BP_FAT	GO:0001737~establishment of imaginal disc-derived wing hair orientation	11	0,2	6,49E-03	1,00E+00	6,21E-02
GOTERM_BP_FAT	GO:0016476~regulation of embryonic cell shape	11	0,2	6,49E-03	1,00E+00	6,21E-02
GOTERM_BP_FAT	GO:0001894~tissue homeostasis	11	0,2	6,49E-03	1,00E+00	6,21E-02
GOTERM_BP_FAT	GO:0007623~circadian rhythm	23	0,5	6,58E-03	1,00E+00	6,27E-02
GOTERM_BP_FAT	GO:0045610~regulation of hemocyte differentiation	13	0,3	6,61E-03	1,00E+00	6,28E-02
GOTERM_BP_FAT	GO:0032880~regulation of protein localization	19	0,4	6,89E-03	1,00E+00	6,52E-02
GOTERM_BP_FAT	GO:0008354~germ cell migration	19	0,4	6,89E-03	1,00E+00	6,52E-02
GOTERM_BP_FAT	GO:0045892~negative regulation of transcription, DNA-dependent	68	1,5	7,17E-03	1,00E+00	6,76E-02
GOTERM_BP_FAT	GO:0048588~developmental cell growth	16	0,4	7,18E-03	1,00E+00	6,74E-02
GOTERM_BP_FAT	GO:0016568~chromatin modification	44	1,0	7,55E-03	1,00E+00	7,05E-02
GOTERM_BP_FAT	GO:0019730~antimicrobial humoral response	30	0,7	7,98E-03	1,00E+00	7,42E-02
GOTERM_BP_FAT	GO:0007530~sex determination	14	0,3	8,02E-03	1,00E+00	7,44E-02
GOTERM_BP_FAT	GO:0009266~response to temperature stimulus	31	0,7	8,06E-03	1,00E+00	7,44E-02
GOTERM_BP_FAT	GO:0048634~regulation of muscle development	17	0,4	8,07E-03	1,00E+00	7,44E-02
GOTERM_BP_FAT	GO:0051223~regulation of protein transport	17	0,4	8,07E-03	1,00E+00	7,44E-02
GOTERM_BP_FAT	GO:0070201~regulation of establishment of protein localization	17	0,4	8,07E-03	1,00E+00	7,44E-02
GOTERM_BP_FAT	GO:0046822~regulation of nucleocytoplasmic transport	17	0,4	8,07E-03	1,00E+00	7,44E-02
GOTERM_BP_FAT	GO:0051640~organelle localization	34	0,8	8,15E-03	1,00E+00	7,48E-02
GOTERM_BP_FAT	GO:0051293~establishment of spindle localization	10	0,2	8,24E-03	1,00E+00	7,54E-02
GOTERM_BP_FAT	GO:0007432~salivary gland boundary specification	10	0,2	8,24E-03	1,00E+00	7,54E-02
GOTERM_BP_FAT	GO:0050905~neuromuscular process	10	0,2	8,24E-03	1,00E+00	7,54E-02
GOTERM_BP_FAT	GO:0016199~axon midline choice point recognition	10	0,2	8,24E-03	1,00E+00	7,54E-02
GOTERM_BP_FAT	GO:0051653~spindle localization	10	0,2	8,24E-03	1,00E+00	7,54E-02
GOTERM_BP_FAT	GO:0031023~microtubule organizing center organization	22	0,5	8,38E-03	1,00E+00	7,63E-02
GOTERM_BP_FAT	GO:0045197~establishment or maintenance of epithelial cell apical/basal polarity	12	0,3	8,54E-03	1,00E+00	7,75E-02
GOTERM_BP_FAT	GO:0008347~glial cell migration	12	0,3	8,54E-03	1,00E+00	7,75E-02
GOTERM_BP_FAT	GO:0043069~negative regulation of programmed cell death	23	0,5	8,75E-03	1,00E+00	7,91E-02
GOTERM_BP_FAT	GO:0007613~memory	18	0,4	8,87E-03	1,00E+00	7,99E-02
GOTERM_BP_FAT	GO:0007173~epidermal growth factor receptor signaling pathway	18	0,4	8,87E-03	1,00E+00	7,99E-02
GOTERM_BP_FAT	GO:0007098~centrosome cycle	18	0,4	8,87E-03	1,00E+00	7,99E-02
GOTERM_BP_FAT	GO:0035150~regulation of tube size	13	0,3	1,04E-02	1,00E+00	9,29E-02
GOTERM_BP_FAT	GO:0018993~somatic sex determination	11	0,2	1,10E-02	1,00E+00	9,80E-02
GOTERM_BP_FAT	GO:0006914~autophagy	11	0,2	1,10E-02	1,00E+00	9,80E-02
GOTERM_BP_FAT	GO:0048675~axon extension	11	0,2	1,10E-02	1,00E+00	9,80E-02
GOTERM_BP_FAT	GO:0016334~establishment or maintenance of polarity of follicular epithelium	11	0,2	1,10E-02	1,00E+00	9,80E-02
GOTERM_BP_FAT	GO:0032386~regulation of intracellular transport	17	0,4	1,14E-02	1,00E+00	1,01E-01
GOTERM_BP_FAT	GO:0007626~locomotory behavior	65	1,5	1,16E-02	1,00E+00	1,02E-01
GOTERM_BP_FAT	GO:0006333~chromatin assembly or disassembly	25	0,6	1,20E-02	1,00E+00	1,05E-01
GOTERM_BP_FAT	GO:0010629~negative regulation of gene expression	88	2,0	1,20E-02	1,00E+00	1,05E-01
GOTERM_BP_FAT	GO:0008361~regulation of cell size	31	0,7	1,25E-02	1,00E+00	1,08E-01
GOTERM_BP_FAT	GO:0016339~calcium-dependent cell-cell adhesion	12	0,3	1,35E-02	1,00E+00	1,17E-01
GOTERM_BP_FAT	GO:0046545~development of primary female sexual characteristics	10	0,2	1,43E-02	1,00E+00	1,23E-01
GOTERM_BP_FAT	GO:0045746~negative regulation of Notch signaling pathway	10	0,2	1,43E-02	1,00E+00	1,23E-01
GOTERM_BP_FAT	GO:0051303~establishment of chromosome localization	10	0,2	1,43E-02	1,00E+00	1,23E-01
GOTERM_BP_FAT	GO:0050000~chromosome localization	10	0,2	1,43E-02	1,00E+00	1,23E-01
GOTERM_BP_FAT	GO:0007474~imaginal disc-derived wing vein specification	16	0,4	1,47E-02	1,00E+00	1,26E-01
GOTERM_BP_FAT	GO:0007612~learning	24	0,5	1,51E-02	1,00E+00	1,28E-01
GOTERM_BP_FAT	GO:0042335~cuticle development	27	0,6	1,54E-02	1,00E+00	1,31E-01
GOTERM_BP_FAT	GO:0016203~muscle attachment	13	0,3	1,57E-02	1,00E+00	1,32E-01
GOTERM_BP_FAT	GO:0045786~negative regulation of cell cycle	13	0,3	1,57E-02	1,00E+00	1,32E-01
GOTERM_BP_FAT	GO:0048542~lymph gland development	13	0,3	1,57E-02	1,00E+00	1,32E-01
GOTERM_BP_FAT	GO:0010627~regulation of protein kinase cascade	20	0,5	1,79E-02	1,00E+00	1,49E-01
GOTERM_BP_FAT	GO:0042330~taxis	20	0,5	1,79E-02	1,00E+00	1,49E-01
GOTERM_BP_FAT	GO:0050953~sensory perception of light stimulus	29	0,7	1,90E-02	1,00E+00	1,57E-01
GOTERM_BP_FAT	GO:0001704~formation of primary germ layer	15	0,3	1,90E-02	1,00E+00	1,57E-01
GOTERM_BP_FAT	GO:0051146~striated muscle cell differentiation	27	0,6	1,92E-02	1,00E+00	1,58E-01
GOTERM_BP_FAT	GO:0006366~transcription from RNA polymerase II promoter	38	0,9	1,99E-02	1,00E+00	1,63E-01
GOTERM_BP_FAT	GO:0032535~regulation of cellular component size	38	0,9	1,99E-02	1,00E+00	1,63E-01
GOTERM_BP_FAT	GO:0007416~synaptogenesis	12	0,3	2,05E-02	1,00E+00	1,67E-01
GOTERM_BP_FAT	GO:0007616~long-term memory	12	0,3	2,05E-02	1,00E+00	1,67E-01
GOTERM_BP_FAT	GO:0007362~terminal region determination	12	0,3	2,05E-02	1,00E+00	1,67E-01
GOTERM_BP_FAT	GO:0006164~purine nucleotide biosynthetic process	46	1,0	2,24E-02	1,00E+00	1,80E-01
GOTERM_BP_FAT	GO:0051325~interphase	13	0,3	2,28E-02	1,00E+00	1,83E-01
GOTERM_BP_FAT	GO:0051329~interphase of mitotic cell cycle	13	0,3	2,28E-02	1,00E+00	1,83E-01
GOTERM_BP_FAT	GO:0045465~R8 cell differentiation	10	0,2	2,31E-02	1,00E+00	1,85E-01
GOTERM_BP_FAT	GO:0044093~positive regulation of molecular function	29	0,7	2,31E-02	1,00E+00	1,84E-01
GOTERM_BP_FAT	GO:0008355~olfactory learning	21	0,5	2,35E-02	1,00E+00	1,87E-01
GOTERM_BP_FAT	GO:0060548~negative regulation of cell death	23	0,5	2,38E-02	1,00E+00	1,89E-01
GOTERM_BP_FAT	GO:0045132~meiotic chromosome segregation	24	0,5	2,39E-02	1,00E+00	1,88E-01
GOTERM_BP_FAT	GO:0042127~regulation of cell proliferation	24	0,5	2,39E-02	1,00E+00	1,88E-01
GOTERM_BP_FAT	GO:0042752~regulation of circadian rhythm	14	0,3	2,46E-02	1,00E+00	1,93E-01
GOTERM_BP_FAT	GO:0042078~germ-line stem cell division	14	0,3	2,46E-02	1,00E+00	1,93E-01
GOTERM_BP_FAT	GO:0031098~stress-activated protein kinase signaling pathway	15	0,3	2,61E-02	1,00E+00	2,03E-01
GOTERM_BP_FAT	GO:0048747~muscle fiber development	15	0,3	2,61E-02	1,00E+00	2,03E-01
GOTERM_BP_FAT	GO:0035151~regulation of tube size, open tracheal system	11	0,2	2,68E-02	1,00E+00	2,07E-01
GOTERM_BP_FAT	GO:0007448~anterior/posterior pattern formation, imaginal disc	11	0,2	2,68E-02	1,00E+00	2,07E-01
GOTERM_BP_FAT	GO:0032774~RNA biosynthetic process	49	1,1	2,71E-02	1,00E+00	2,09E-01
GOTERM_BP_FAT	GO:0016321~female meiosis chromosome segregation	16	0,4	2,73E-02	1,00E+00	2,10E-01
GOTERM_BP_FAT	GO:0008586~imaginal disc-derived wing vein morphogenesis	16	0,4	2,73E-02	1,00E+00	2,10E-01
GOTERM_BP_FAT	GO:0006352~transcription initiation	29	0,7	2,79E-02	1,00E+00	2,13E-01
GOTERM_BP_FAT	GO:0006351~transcription, DNA-dependent	48	1,1	2,79E-02	1,00E+00	2,13E-01
GOTERM_BP_FAT	GO:0007601~visual perception	28	0,6	2,83E-02	1,00E+00	2,15E-01
GOTERM_BP_FAT	GO:0006163~purine nucleotide metabolic process	47	1,1	2,87E-02	1,00E+00	2,17E-01
GOTERM_BP_FAT	GO:0030036~actin cytoskeleton organization	51	1,1	2,91E-02	1,00E+00	2,20E-01
GOTERM_BP_FAT	GO:0060249~anatomical structure homeostasis	19	0,4	2,93E-02	1,00E+00	2,20E-01
GOTERM_BP_FAT	GO:0000768~syncytium formation by plasma membrane fusion	12	0,3	2,97E-02	1,00E+00	2,22E-01
GOTERM_BP_FAT	GO:0014902~myotube differentiation	12	0,3	2,97E-02	1,00E+00	2,22E-01
GOTERM_BP_FAT	GO:0007520~myoblast fusion	12	0,3	2,97E-02	1,00E+00	2,22E-01
GOTERM_BP_FAT	GO:0009611~response to wounding	12	0,3	2,97E-02	1,00E+00	2,22E-01
GOTERM_BP_FAT	GO:0006643~membrane lipid metabolic process	12	0,3	2,97E-02	1,00E+00	2,22E-01
GOTERM_BP_FAT	GO:0006949~syncytium formation	12	0,3	2,97E-02	1,00E+00	2,22E-01
GOTERM_BP_FAT	GO:0035265~organ growth	12	0,3	2,97E-02	1,00E+00	2,22E-01

GOTERM_BP_FAT	GO:0016569~covalent chromatin modification	23	0,5	2,97E-02	1,00E+00	2,22E-01
GOTERM_BP_FAT	GO:0016570~histone modification	23	0,5	2,97E-02	1,00E+00	2,22E-01
GOTERM_BP_FAT	GO:0007015~actin filament organization	32	0,7	3,15E-02	1,00E+00	2,33E-01
GOTERM_BP_FAT	GO:0006959~humoral immune response	32	0,7	3,15E-02	1,00E+00	2,33E-01
GOTERM_BP_FAT	GO:0030029~actin filament-based process	51	1,1	3,30E-02	1,00E+00	2,43E-01
GOTERM_BP_FAT	GO:0006334~nucleosome assembly	14	0,3	3,37E-02	1,00E+00	2,46E-01
GOTERM_BP_FAT	GO:0007254~JNK cascade	14	0,3	3,37E-02	1,00E+00	2,46E-01
GOTERM_BP_FAT	GO:0045926~negative regulation of growth	14	0,3	3,37E-02	1,00E+00	2,46E-01
GOTERM_BP_FAT	GO:0007354~zygotic determination of anterior/posterior axis, embryo	14	0,3	3,37E-02	1,00E+00	2,46E-01
GOTERM_BP_FAT	GO:0009259~ribonucleotide metabolic process	41	0,9	3,41E-02	1,00E+00	2,48E-01
GOTERM_BP_FAT	GO:0048619~embryonic hindgut morphogenesis	15	0,3	3,50E-02	1,00E+00	2,53E-01
GOTERM_BP_FAT	GO:0006323~DNA packaging	26	0,6	3,52E-02	1,00E+00	2,54E-01
GOTERM_BP_FAT	GO:0015849~organic acid transport	22	0,5	3,69E-02	1,00E+00	2,64E-01
GOTERM_BP_FAT	GO:0046942~carboxylic acid transport	22	0,5	3,69E-02	1,00E+00	2,64E-01
GOTERM_BP_FAT	GO:0009124~nucleoside monophosphate biosynthetic process	21	0,5	3,71E-02	1,00E+00	2,65E-01
GOTERM_BP_FAT	GO:0051235~maintenance of location	20	0,5	3,72E-02	1,00E+00	2,65E-01
GOTERM_BP_FAT	GO:0048134~germ-line cyst formation	11	0,2	3,88E-02	1,00E+00	2,74E-01
GOTERM_BP_FAT	GO:0042990~regulation of transcription factor import into nucleus	11	0,2	3,88E-02	1,00E+00	2,74E-01
GOTERM_BP_FAT	GO:0046700~heterocycle catabolic process	11	0,2	3,88E-02	1,00E+00	2,74E-01
GOTERM_BP_FAT	GO:0009150~purine ribonucleotide metabolic process	39	0,9	4,15E-02	1,00E+00	2,89E-01
GOTERM_BP_FAT	GO:0009260~ribonucleotide biosynthetic process	39	0,9	4,15E-02	1,00E+00	2,89E-01
GOTERM_BP_FAT	GO:0007265~Ras protein signal transduction	13	0,3	4,35E-02	1,00E+00	3,01E-01
GOTERM_BP_FAT	GO:0016202~regulation of striated muscle tissue development	14	0,3	4,49E-02	1,00E+00	3,08E-01
GOTERM_BP_FAT	GO:0051248~negative regulation of protein metabolic process	22	0,5	4,52E-02	1,00E+00	3,09E-01
GOTERM_BP_FAT	GO:0009123~nucleoside monophosphate metabolic process	22	0,5	4,52E-02	1,00E+00	3,09E-01
GOTERM_BP_FAT	GO:0031497~chromatin assembly	16	0,4	4,64E-02	1,00E+00	3,16E-01
GOTERM_BP_FAT	GO:0043066~negative regulation of apoptosis	19	0,4	4,65E-02	1,00E+00	3,16E-01
GOTERM_BP_FAT	GO:0000226~microtubule cytoskeleton organization	96	2,2	4,96E-02	1,00E+00	3,32E-01
GOTERM_BP_FAT	GO:0009152~purine ribonucleotide biosynthetic process	37	0,8	5,05E-02	1,00E+00	3,36E-01
GOTERM_BP_FAT	GO:0001666~response to hypoxia	10	0,2	5,08E-02	1,00E+00	3,38E-01
GOTERM_BP_FAT	GO:0070482~response to oxygen levels	10	0,2	5,08E-02	1,00E+00	3,38E-01
GOTERM_BP_FAT	GO:0006897~endocytosis	84	1,9	5,20E-02	1,00E+00	3,44E-01
GOTERM_BP_FAT	GO:0010324~membrane invagination	84	1,9	5,20E-02	1,00E+00	3,44E-01
GOTERM_BP_FAT	GO:0016044~membrane organization	102	2,3	5,21E-02	1,00E+00	3,43E-01
GOTERM_BP_FAT	GO:0007610~behavior	134	3,0	5,39E-02	1,00E+00	3,52E-01
GOTERM_BP_FAT	GO:0007446~imaginal disc growth	11	0,2	5,40E-02	1,00E+00	3,52E-01
GOTERM_BP_FAT	GO:0035023~regulation of Rho protein signal transduction	13	0,3	5,76E-02	1,00E+00	3,70E-01
GOTERM_BP_FAT	GO:0007622~rhythmic behavior	17	0,4	5,85E-02	1,00E+00	3,74E-01
GOTERM_BP_FAT	GO:0043408~regulation of MAPKKK cascade	15	0,3	5,88E-02	1,00E+00	3,75E-01
GOTERM_BP_FAT	GO:0045185~maintenance of protein location	16	0,4	5,88E-02	1,00E+00	3,74E-01
GOTERM_BP_FAT	GO:0051129~negative regulation of cellular component organization	22	0,5	6,56E-02	1,00E+00	4,08E-01
GOTERM_BP_FAT	GO:0032269~negative regulation of cellular protein metabolic process	21	0,5	6,71E-02	1,00E+00	4,14E-01
GOTERM_BP_FAT	GO:0006820~anion transport	19	0,4	6,99E-02	1,00E+00	4,26E-01
GOTERM_BP_FAT	GO:0010948~negative regulation of cell cycle process	10	0,2	7,05E-02	1,00E+00	4,29E-01
GOTERM_BP_FAT	GO:0007390~germ-band shortening	10	0,2	7,05E-02	1,00E+00	4,29E-01
GOTERM_BP_FAT	GO:0009165~nucleotide biosynthetic process	51	1,1	7,26E-02	1,00E+00	4,37E-01
GOTERM_BP_FAT	GO:0035075~response to ecdysone	11	0,2	7,28E-02	1,00E+00	4,37E-01
GOTERM_BP_FAT	GO:0007097~nuclear migration	11	0,2	7,28E-02	1,00E+00	4,37E-01
GOTERM_BP_FAT	GO:0048545~response to steroid hormone stimulus	11	0,2	7,28E-02	1,00E+00	4,37E-01
GOTERM_BP_FAT	GO:0051345~positive regulation of hydrolase activity	11	0,2	7,28E-02	1,00E+00	4,37E-01
GOTERM_BP_FAT	GO:0048512~circadian behavior	16	0,4	7,33E-02	1,00E+00	4,39E-01
GOTERM_BP_FAT	GO:0031344~regulation of cell projection organization	16	0,4	7,33E-02	1,00E+00	4,39E-01
GOTERM_BP_FAT	GO:0051050~positive regulation of transport	12	0,3	7,40E-02	1,00E+00	4,41E-01
GOTERM_BP_FAT	GO:0040023~establishment of nucleus localization	12	0,3	7,40E-02	1,00E+00	4,41E-01
GOTERM_BP_FAT	GO:0051651~maintenance of location in cell	15	0,3	7,41E-02	1,00E+00	4,41E-01
GOTERM_BP_FAT	GO:0070302~regulation of stress-activated protein kinase signaling pathway	14	0,3	7,45E-02	1,00E+00	4,42E-01
GOTERM_BP_FAT	GO:0046328~regulation of JNK cascade	14	0,3	7,45E-02	1,00E+00	4,42E-01
GOTERM_BP_FAT	GO:0034654~nucleobase, nucleoside, nucleotide and nucleic acid biosynthetic process	53	1,2	7,51E-02	1,00E+00	4,44E-01
GOTERM_BP_FAT	GO:0034404~nucleobase, nucleoside and nucleotide biosynthetic process	53	1,2	7,51E-02	1,00E+00	4,44E-01
GOTERM_BP_FAT	GO:0009141~nucleoside triphosphate metabolic process	32	0,7	7,72E-02	1,00E+00	4,52E-01
GOTERM_BP_FAT	GO:0007049~cell cycle	186	4,2	7,75E-02	1,00E+00	4,53E-01
GOTERM_BP_FAT	GO:0007281~germ cell development	78	1,8	7,99E-02	1,00E+00	4,63E-01
GOTERM_BP_FAT	GO:0045995~regulation of embryonic development	21	0,5	8,00E-02	1,00E+00	4,62E-01
GOTERM_BP_FAT	GO:0017038~protein import	26	0,6	8,12E-02	1,00E+00	4,67E-01
GOTERM_BP_FAT	GO:0034613~cellular protein localization	59	1,3	8,18E-02	1,00E+00	4,68E-01
GOTERM_BP_FAT	GO:0002168~instar larval development	19	0,4	8,42E-02	1,00E+00	4,77E-01
GOTERM_BP_FAT	GO:0000910~cytokinesis	29	0,7	8,51E-02	1,00E+00	4,80E-01
GOTERM_BP_FAT	GO:0043473~pigmentation	24	0,5	8,63E-02	1,00E+00	4,85E-01
GOTERM_BP_FAT	GO:0043085~positive regulation of catalytic activity	24	0,5	8,63E-02	1,00E+00	4,85E-01
GOTERM_BP_FAT	GO:0006952~defense response	57	1,3	8,73E-02	1,00E+00	4,88E-01
GOTERM_BP_FAT	GO:0015837~amine transport	17	0,4	8,81E-02	1,00E+00	4,90E-01
GOTERM_BP_FAT	GO:0006909~phagocytosis	64	1,4	9,02E-02	1,00E+00	4,98E-01
GOTERM_BP_FAT	GO:0009628~response to abiotic stimulus	69	1,6	9,05E-02	1,00E+00	4,98E-01
GOTERM_BP_FAT	GO:0010004~gastrulation involving germ band extension	15	0,3	9,17E-02	1,00E+00	5,02E-01
GOTERM_BP_FAT	GO:0001703~gastrulation with mouth forming first	15	0,3	9,17E-02	1,00E+00	5,02E-01
GOTERM_BP_FAT	GO:0046843~dorsal appendage formation	15	0,3	9,17E-02	1,00E+00	5,02E-01
GOTERM_BP_FAT	GO:0080135~regulation of cellular response to stress	14	0,3	9,31E-02	1,00E+00	5,07E-01
GOTERM_BP_FAT	GO:0016050~vesicle organization	14	0,3	9,31E-02	1,00E+00	5,07E-01
GOTERM_BP_FAT	GO:0032507~maintenance of protein location in cell	14	0,3	9,31E-02	1,00E+00	5,07E-01
GOTERM_BP_FAT	GO:0010975~regulation of neuron projection development	13	0,3	9,43E-02	1,00E+00	5,11E-01
GOTERM_BP_FAT	GO:0010769~regulation of cell morphogenesis involved in differentiation	13	0,3	9,43E-02	1,00E+00	5,11E-01
GOTERM_BP_FAT	GO:0051647~nucleus localization	13	0,3	9,43E-02	1,00E+00	5,11E-01
GOTERM_BP_FAT	GO:0009156~ribonucleoside monophosphate biosynthetic process	10	0,2	9,44E-02	1,00E+00	5,10E-01
GOTERM_BP_FAT	GO:0006916~anti-apoptosis	10	0,2	9,44E-02	1,00E+00	5,10E-01
GOTERM_BP_FAT	GO:0009161~ribonucleoside monophosphate metabolic process	10	0,2	9,44E-02	1,00E+00	5,10E-01
GOTERM_BP_FAT	GO:0016571~histone methylation	10	0,2	9,44E-02	1,00E+00	5,10E-01
GOTERM_BP_FAT	GO:0030071~regulation of mitotic metaphase/anaphase transition	10	0,2	9,44E-02	1,00E+00	5,10E-01
GOTERM_BP_FAT	GO:0009798~axis specification	65	1,5	9,49E-02	1,00E+00	5,11E-01
GOTERM_BP_FAT	GO:0045475~locomotor rhythm	12	0,3	9,50E-02	1,00E+00	5,11E-01
GOTERM_BP_FAT	GO:0019731~antibacterial humoral response	12	0,3	9,50E-02	1,00E+00	5,11E-01
GOTERM_BP_FAT	GO:0007443~Malpighian tubule morphogenesis	12	0,3	9,50E-02	1,00E+00	5,11E-01
GOTERM_BP_FAT	GO:0006886~intracellular protein transport	57	1,3	9,54E-02	1,00E+00	5,12E-01
GOTERM_BP_FAT	GO:0060341~regulation of cellular localization	25	0,6	9,70E-02	1,00E+00	5,17E-01
GOTERM_CC_FAT	GO:0015630~microtubule cytoskeleton	123	2,8	2,32E-18	1,32E-15	1,32E-15
GOTERM_CC_FAT	GO:0005856~cytoskeleton	166	3,7	5,17E-15	2,97E-12	1,49E-12

GOTERM_CC_FAT	GO:0005886~plasma membrane	312	7,0	1,00E-14	5,70E-12	1,90E-12
GOTERM_CC_FAT	GO:0044430~cytoskeletal part	138	3,1	3,23E-13	1,84E-10	4,60E-11
GOTERM_CC_FAT	GO:0044459~plasma membrane part	175	3,9	1,93E-11	1,10E-08	2,20E-09
GOTERM_CC_FAT	GO:0005815~microtubule organizing center	42	0,9	9,65E-11	5,50E-08	9,16E-09
GOTERM_CC_FAT	GO:0000502~proteasome complex	40	0,9	1,27E-09	7,24E-07	1,03E-07
GOTERM_CC_FAT	GO:0005911~cell-cell junction	38	0,9	3,47E-09	1,98E-06	2,47E-07
GOTERM_CC_FAT	GO:0005875~microtubule associated complex	63	1,4	3,88E-09	2,21E-06	2,46E-07
GOTERM_CC_FAT	GO:0005813~centrosome	36	0,8	4,40E-09	2,51E-06	2,51E-07
GOTERM_CC_FAT	GO:0045179~apical cortex	20	0,5	1,98E-08	1,13E-05	1,03E-06
GOTERM_CC_FAT	GO:0005838~proteasome regulatory particle	21	0,5	2,80E-08	1,60E-05	1,33E-06
GOTERM_CC_FAT	GO:0045177~apical part of cell	33	0,7	2,94E-08	1,67E-05	1,29E-06
GOTERM_CC_FAT	GO:0022624~proteasome accessory complex	21	0,5	1,03E-07	5,87E-05	4,19E-06
GOTERM_CC_FAT	GO:0016327~apicolateral plasma membrane	29	0,7	1,81E-06	1,03E-03	6,88E-05
GOTERM_CC_FAT	GO:0030054~cell junction	64	1,4	2,04E-06	1,16E-03	7,27E-05
GOTERM_CC_FAT	GO:0043296~apical junction complex	27	0,6	2,54E-06	1,45E-03	8,51E-05
GOTERM_CC_FAT	GO:0005938~cell cortex	41	0,9	3,38E-06	1,93E-03	1,07E-04
GOTERM_CC_FAT	GO:0005819~spindle	38	0,9	4,23E-06	2,41E-03	1,27E-04
GOTERM_CC_FAT	GO:0044448~cell cortex part	28	0,6	1,33E-05	7,56E-03	3,79E-04
GOTERM_CC_FAT	GO:0005887~integral to plasma membrane	84	1,9	1,59E-05	9,00E-03	4,31E-04
GOTERM_CC_FAT	GO:0031226~intrinsic to plasma membrane	85	1,9	1,62E-05	9,19E-03	4,20E-04
GOTERM_CC_FAT	GO:0005694~chromosome	119	2,7	2,27E-05	1,29E-02	5,62E-04
GOTERM_CC_FAT	GO:0008540~proteasome regulatory particle, base subcomplex	11	0,2	2,53E-05	1,43E-02	6,01E-04
GOTERM_CC_FAT	GO:0044427~chromosomal part	98	2,2	3,47E-05	1,96E-02	7,91E-04
GOTERM_CC_FAT	GO:0005912~adherens junction	27	0,6	7,88E-05	4,39E-02	1,73E-03
GOTERM_CC_FAT	GO:0005770~late endosome	11	0,2	1,13E-04	6,26E-02	2,39E-03
GOTERM_CC_FAT	GO:0070161~anchoring junction	27	0,6	1,26E-04	6,95E-02	2,57E-03
GOTERM_CC_FAT	GO:0005871~kinesin complex	19	0,4	2,58E-04	1,37E-01	5,05E-03
GOTERM_CC_FAT	GO:0005874~microtubule	44	1,0	3,33E-04	1,73E-01	6,31E-03
GOTERM_CC_FAT	GO:0000785~chromatin	50	1,1	3,40E-04	1,76E-01	6,24E-03
GOTERM_CC_FAT	GO:0005913~cell-cell adherens junction	11	0,2	3,67E-04	1,89E-01	6,51E-03
GOTERM_CC_FAT	GO:0030286~dynein complex	24	0,5	3,94E-04	2,01E-01	6,79E-03
GOTERM_CC_FAT	GO:0042995~cell projection	49	1,1	4,21E-04	2,14E-01	7,04E-03
GOTERM_CC_FAT	GO:0044450~microtubule organizing center part	15	0,3	5,49E-04	2,69E-01	8,90E-03
GOTERM_CC_FAT	GO:0043005~neuron projection	24	0,5	6,08E-04	2,93E-01	9,59E-03
GOTERM_CC_FAT	GO:0000775~chromosome, centromeric region	26	0,6	8,45E-04	3,82E-01	1,29E-02
GOTERM_CC_FAT	GO:0031224~intrinsic to membrane	434	9,8	1,21E-03	4,99E-01	1,80E-02
GOTERM_CC_FAT	GO:0005918~septate junction	14	0,3	1,27E-03	5,14E-01	1,84E-02
GOTERM_CC_FAT	GO:0070160~occluding junction	16	0,4	1,49E-03	5,73E-01	2,10E-02
GOTERM_CC_FAT	GO:0031461~cullin-RING ubiquitin ligase complex	13	0,3	1,53E-03	5,82E-01	2,11E-02
GOTERM_CC_FAT	GO:0009986~cell surface	11	0,2	2,15E-03	7,07E-01	2,88E-02
GOTERM_CC_FAT	GO:0000151~ubiquitin ligase complex	21	0,5	2,75E-03	7,92E-01	3,58E-02
GOTERM_CC_FAT	GO:0044463~cell projection part	24	0,5	2,77E-03	7,94E-01	3,52E-02
GOTERM_CC_FAT	GO:0031225~anchored to membrane	12	0,3	3,51E-03	8,65E-01	4,36E-02
GOTERM_CC_FAT	GO:0016324~apical plasma membrane	14	0,3	3,95E-03	8,95E-01	4,78E-02
GOTERM_CC_FAT	GO:0005768~endosome	17	0,4	4,82E-03	9,36E-01	5,69E-02
GOTERM_CC_FAT	GO:0030424~axon	15	0,3	5,05E-03	9,44E-01	5,84E-02
GOTERM_CC_FAT	GO:0016021~integral to membrane	421	9,5	5,14E-03	9,47E-01	5,82E-02
GOTERM_CC_FAT	GO:0005700~polytene chromosome	37	0,8	1,44E-02	1,00E+00	1,53E-01
GOTERM_CC_FAT	GO:0043232~intracellular non-membrane-bounded organelle	294	6,6	1,61E-02	1,00E+00	1,66E-01
GOTERM_CC_FAT	GO:0043228~non-membrane-bounded organelle	294	6,6	1,61E-02	1,00E+00	1,66E-01
GOTERM_CC_FAT	GO:0030425~dendrite	12	0,3	1,63E-02	1,00E+00	1,65E-01
GOTERM_CC_FAT	GO:0000776~kinetochore	15	0,3	1,68E-02	1,00E+00	1,66E-01
GOTERM_CC_FAT	GO:0000793~condensed chromosome	16	0,4	1,84E-02	1,00E+00	1,78E-01
GOTERM_CC_FAT	GO:0008287~protein serine/threonine phosphatase complex	14	0,3	2,15E-02	1,00E+00	2,01E-01
GOTERM_CC_FAT	GO:0044421~extracellular region part	55	1,2	2,40E-02	1,00E+00	2,19E-01
GOTERM_CC_FAT	GO:0019897~extrinsic to plasma membrane	17	0,4	2,67E-02	1,00E+00	2,37E-01
GOTERM_CC_FAT	GO:0005839~proteasome core complex	13	0,3	2,75E-02	1,00E+00	2,40E-01
GOTERM_CC_FAT	GO:0000922~spindle pole	12	0,3	3,53E-02	1,00E+00	2,93E-01
GOTERM_CC_FAT	GO:0033179~proton-transporting V-type ATPase, V0 domain	10	0,2	4,08E-02	1,00E+00	3,27E-01
GOTERM_CC_FAT	GO:0005667~transcription factor complex	31	0,7	4,34E-02	1,00E+00	3,40E-01
GOTERM_CC_FAT	GO:0031012~extracellular matrix	22	0,5	4,70E-02	1,00E+00	3,58E-01
GOTERM_CC_FAT	GO:0045202~synapse	44	1,0	7,35E-02	1,00E+00	4,99E-01
GOTERM_CC_FAT	GO:0000792~heterochromatin	10	0,2	8,08E-02	1,00E+00	5,28E-01
GOTERM_CC_FAT	GO:0005578~proteinaceous extracellular matrix	20	0,5	8,45E-02	1,00E+00	5,39E-01
GOTERM_CC_FAT	GO:0033177~proton-transporting two-sector ATPase complex, proton-transporting domain	14	0,3	8,80E-02	1,00E+00	5,49E-01
GOTERM_MF_FAT	GO:0030528~transcription regulator activity	301	6,8	2,69E-16	2,72E-13	2,72E-13
GOTERM_MF_FAT	GO:0003700~transcription factor activity	192	4,3	3,11E-16	4,09E-13	2,04E-13
GOTERM_MF_FAT	GO:0003677~DNA binding	346	7,8	3,40E-14	4,17E-11	1,39E-11
GOTERM_MF_FAT	GO:0003702~RNA polymerase II transcription factor activity	134	3,0	9,30E-14	1,14E-10	2,85E-11
GOTERM_MF_FAT	GO:0043565~sequence-specific DNA binding	122	2,7	2,81E-13	3,45E-10	6,90E-11
GOTERM_MF_FAT	GO:0008270~zinc ion binding	402	9,1	1,21E-11	1,48E-08	2,47E-09
GOTERM_MF_FAT	GO:0003704~specific RNA polymerase II transcription factor activity	50	1,1	8,60E-10	1,06E-06	1,51E-07
GOTERM_MF_FAT	GO:0016881~acid-amino acid ligase activity	64	1,4	1,29E-08	1,58E-05	1,98E-06
GOTERM_MF_FAT	GO:0004842~ubiquitin-protein ligase activity	50	1,1	3,13E-08	3,85E-05	4,27E-06
GOTERM_MF_FAT	GO:0019787~small conjugating protein ligase activity	52	1,2	6,44E-08	7,90E-05	7,90E-06
GOTERM_MF_FAT	GO:0046872~metal ion binding	605	13,6	7,61E-08	9,33E-05	8,49E-06
GOTERM_MF_FAT	GO:0016564~transcription repressor activity	60	1,4	1,01E-07	1,24E-04	1,03E-05
GOTERM_MF_FAT	GO:0043167~ion binding	620	14,0	1,48E-07	1,81E-04	1,40E-05
GOTERM_MF_FAT	GO:0043169~cation binding	616	13,9	2,34E-07	2,87E-04	2,05E-05
GOTERM_MF_FAT	GO:0008092~cytoskeletal protein binding	101	2,3	3,62E-07	4,44E-04	2,96E-05
GOTERM_MF_FAT	GO:0004672~protein kinase activity	121	2,7	4,02E-06	4,92E-03	3,08E-04
GOTERM_MF_FAT	GO:0016879~ligase activity, forming carbon-nitrogen bonds	68	1,5	5,27E-06	6,45E-03	3,81E-04
GOTERM_MF_FAT	GO:0046914~transition metal ion binding	474	10,7	7,77E-06	9,49E-03	5,29E-04
GOTERM_MF_FAT	GO:0004713~protein tyrosine kinase activity	36	0,8	1,90E-05	2,30E-02	1,22E-03
GOTERM_MF_FAT	GO:0015631~tubulin binding	38	0,9	2,07E-05	2,51E-02	1,27E-03
GOTERM_MF_FAT	GO:0008017~microtubule binding	35	0,8	5,57E-05	6,61E-02	3,25E-03
GOTERM_MF_FAT	GO:0004674~protein serine/threonine kinase activity	86	1,9	1,70E-04	1,88E-01	9,42E-03
GOTERM_MF_FAT	GO:0004721~phosphoprotein phosphatase activity	46	1,0	3,25E-04	3,29E-01	1,72E-02
GOTERM_MF_FAT	GO:0004714~transmembrane receptor protein tyrosine kinase activity	16	0,4	4,90E-04	4,52E-01	2,47E-02
GOTERM_MF_FAT	GO:0019899~enzyme binding	33	0,7	1,13E-03	7,49E-01	5,39E-02
GOTERM_MF_FAT	GO:0035091~phosphoinositide binding	15	0,3	2,02E-03	9,16E-01	9,09E-02
GOTERM_MF_FAT	GO:0003777~microtubule motor activity	28	0,6	2,96E-03	9,74E-01	1,26E-01
GOTERM_MF_FAT	GO:0016566~specific transcriptional repressor activity	20	0,5	3,41E-03	9,85E-01	1,39E-01
GOTERM_MF_FAT	GO:0016563~transcription activator activity	41	0,9	4,89E-03	9,98E-01	1,87E-01
GOTERM_MF_FAT	GO:0004725~protein tyrosine phosphatase activity	23	0,5	5,69E-03	9,99E-01	2,08E-01

GOTERM_MF_FAT	GO:0004722~protein serine/threonine phosphatase activity	23	0,5	5,69E-03	9,99E-01	2,08E-01
GOTERM_MF_FAT	GO:0030695~GTPase regulator activity	58	1,3	5,72E-03	9,99E-01	2,03E-01
GOTERM_MF_FAT	GO:0042802~identical protein binding	59	1,3	5,83E-03	9,99E-01	2,01E-01
GOTERM_MF_FAT	GO:0004879~ligand-dependent nuclear receptor activity	14	0,3	7,04E-03	1,00E+00	2,31E-01
GOTERM_MF_FAT	GO:0060589~nucleoside-triphosphatase regulator activity	58	1,3	9,45E-03	1,00E+00	2,90E-01
GOTERM_MF_FAT	GO:0003774~motor activity	38	0,9	1,02E-02	1,00E+00	3,02E-01
GOTERM_MF_FAT	GO:0050839~cell adhesion molecule binding	12	0,3	1,03E-02	1,00E+00	2,97E-01
GOTERM_MF_FAT	GO:0003682~chromatin binding	29	0,7	1,30E-02	1,00E+00	3,52E-01
GOTERM_MF_FAT	GO:0005096~GTPase activator activity	32	0,7	1,51E-02	1,00E+00	3,89E-01
GOTERM_MF_FAT	GO:0005083~small GTPase regulator activity	44	1,0	1,75E-02	1,00E+00	4,26E-01
GOTERM_MF_FAT	GO:0016791~phosphatase activity	69	1,6	1,80E-02	1,00E+00	4,27E-01
GOTERM_MF_FAT	GO:0008047~enzyme activator activity	38	0,9	1,85E-02	1,00E+00	4,28E-01
GOTERM_MF_FAT	GO:0019904~protein domain specific binding	15	0,3	2,02E-02	1,00E+00	4,50E-01
GOTERM_MF_FAT	GO:0046982~protein heterodimerization activity	22	0,5	2,27E-02	1,00E+00	4,80E-01
GOTERM_MF_FAT	GO:0003727~single-stranded RNA binding	12	0,3	2,59E-02	1,00E+00	5,19E-01
GOTERM_MF_FAT	GO:0005509~calcium ion binding	87	2,0	3,30E-02	1,00E+00	5,99E-01
GOTERM_MF_FAT	GO:0030145~manganese ion binding	25	0,6	3,37E-02	1,00E+00	5,99E-01
GOTERM_MF_FAT	GO:0051020~GTPase binding	14	0,3	3,52E-02	1,00E+00	6,07E-01
GOTERM_MF_FAT	GO:0003779~actin binding	47	1,1	3,90E-02	1,00E+00	6,38E-01
GOTERM_MF_FAT	GO:0004298~threonine-type endopeptidase activity	13	0,3	4,34E-02	1,00E+00	6,71E-01
GOTERM_MF_FAT	GO:0070003~threonine-type peptidase activity	13	0,3	4,34E-02	1,00E+00	6,71E-01
GOTERM_MF_FAT	GO:0008234~cysteine-type peptidase activity	27	0,6	5,30E-02	1,00E+00	7,37E-01
GOTERM_MF_FAT	GO:0017022~myosin binding	12	0,3	5,37E-02	1,00E+00	7,35E-01
GOTERM_MF_FAT	GO:0008134~transcription factor binding	35	0,8	5,77E-02	1,00E+00	7,54E-01
GOTERM_MF_FAT	GO:0003729~mRNA binding	67	1,5	5,77E-02	1,00E+00	7,47E-01
GOTERM_MF_FAT	GO:0004221~ubiquitin thiolesterase activity	14	0,3	6,40E-02	1,00E+00	7,78E-01
GOTERM_MF_FAT	GO:0005099~Ras GTPase activator activity	20	0,5	6,64E-02	1,00E+00	7,84E-01
GOTERM_MF_FAT	GO:0008138~protein tyrosine/serine/threonine phosphatase activity	11	0,2	6,66E-02	1,00E+00	7,79E-01
GOTERM_MF_FAT	GO:0003714~transcription corepressor activity	11	0,2	6,66E-02	1,00E+00	7,79E-01
GOTERM_MF_FAT	GO:0031267~small GTPase binding	12	0,3	7,31E-02	1,00E+00	8,05E-01
GOTERM_MF_FAT	GO:0017016~Ras GTPase binding	12	0,3	7,31E-02	1,00E+00	8,05E-01
GOTERM_MF_FAT	GO:0000166~nucleotide binding	384	8,7	7,38E-02	1,00E+00	8,03E-01
GOTERM_MF_FAT	GO:0042624~ATPase activity, uncoupled	68	1,5	7,68E-02	1,00E+00	8,10E-01
GOTERM_MF_FAT	GO:0005543~phospholipid binding	19	0,4	7,93E-02	1,00E+00	8,16E-01
GOTERM_MF_FAT	GO:0003707~steroid hormone receptor activity	10	0,2	8,28E-02	1,00E+00	8,24E-01
GOTERM_MF_FAT	GO:0008083~growth factor activity	14	0,3	8,30E-02	1,00E+00	8,20E-01
GOTERM_MF_FAT	GO:0016251~general RNA polymerase II transcription factor activity	34	0,8	8,84E-02	1,00E+00	8,35E-01
GOTERM_MF_FAT	GO:0008374~O-acyltransferase activity	11	0,2	9,03E-02	1,00E+00	8,37E-01
GOTERM_MF_FAT	GO:0015171~amino acid transmembrane transporter activity	20	0,5	9,80E-02	1,00E+00	8,57E-01

Taf10 - Activation

Category	Term	Number of affected genes	percentage	PValue	Bonferroni	Benjamini
GOTERM_BP_FAT	GO:0055114~oxidation reduction	181	9,3	3,00E-33	5,41E-30	5,41E-30
GOTERM_BP_FAT	GO:0005976~polysaccharide metabolic process	71	3,6	8,04E-26	1,45E-22	7,25E-23
GOTERM_BP_FAT	GO:0006022~aminoglycan metabolic process	67	3,4	6,90E-25	1,25E-21	4,15E-22
GOTERM_BP_FAT	GO:0006030~chitin metabolic process	59	3,0	1,56E-24	2,81E-21	7,02E-22
GOTERM_BP_FAT	GO:0006508~proteolysis	166	8,5	1,24E-17	2,24E-14	4,48E-15
GOTERM_BP_FAT	GO:0006952~defense response	54	2,8	3,58E-11	6,46E-08	1,08E-08
GOTERM_BP_FAT	GO:0045087~innate immune response	35	1,8	5,24E-10	9,45E-07	1,35E-07
GOTERM_BP_FAT	GO:0016052~carbohydrate catabolic process	32	1,6	2,26E-09	4,08E-06	5,09E-07
GOTERM_BP_FAT	GO:0019318~hexose metabolic process	32	1,6	4,43E-09	8,00E-06	8,89E-07
GOTERM_BP_FAT	GO:0005996~monosaccharide metabolic process	34	1,7	1,07E-08	1,93E-05	1,93E-06
GOTERM_BP_FAT	GO:0006955~immune response	47	2,4	4,52E-08	8,16E-05	7,42E-06
GOTERM_BP_FAT	GO:0006006~glucose metabolic process	24	1,2	2,19E-07	3,96E-04	3,30E-05
GOTERM_BP_FAT	GO:0042742~defense response to bacterium	25	1,3	6,84E-07	1,23E-03	9,50E-05
GOTERM_BP_FAT	GO:0009636~response to toxin	14	0,7	7,05E-07	1,27E-03	9,08E-05
GOTERM_BP_FAT	GO:0009617~response to bacterium	27	1,4	1,06E-06	1,92E-03	1,28E-04
GOTERM_BP_FAT	GO:0016053~organic acid biosynthetic process	23	1,2	2,50E-06	4,49E-03	2,81E-04
GOTERM_BP_FAT	GO:0046394~carboxylic acid biosynthetic process	23	1,2	2,50E-06	4,49E-03	2,81E-04
GOTERM_BP_FAT	GO:0017085~response to insecticide	11	0,6	1,06E-05	1,89E-02	1,12E-03
GOTERM_BP_FAT	GO:0044275~cellular carbohydrate catabolic process	19	1,0	1,34E-05	2,39E-02	1,35E-03
GOTERM_BP_FAT	GO:0046164~alcohol catabolic process	19	1,0	1,34E-05	2,39E-02	1,35E-03
GOTERM_BP_FAT	GO:0006096~glycolysis	15	0,8	2,41E-05	4,26E-02	2,29E-03
GOTERM_BP_FAT	GO:0019731~antibacterial humoral response	13	0,7	2,62E-05	4,63E-02	2,37E-03
GOTERM_BP_FAT	GO:0000272~polysaccharide catabolic process	14	0,7	5,71E-05	9,79E-02	4,90E-03
GOTERM_BP_FAT	GO:0006811~ion transport	67	3,4	7,19E-05	1,22E-01	5,88E-03
GOTERM_BP_FAT	GO:0019320~hexose catabolic process	16	0,8	8,19E-05	1,37E-01	6,40E-03
GOTERM_BP_FAT	GO:0006007~glucose catabolic process	16	0,8	8,19E-05	1,37E-01	6,40E-03
GOTERM_BP_FAT	GO:0009064~glutamine family amino acid metabolic process	13	0,7	9,25E-05	1,54E-01	6,93E-03
GOTERM_BP_FAT	GO:0006631~fatty acid metabolic process	18	0,9	9,37E-05	1,56E-01	6,74E-03
GOTERM_BP_FAT	GO:0046365~monosaccharide catabolic process	16	0,8	1,11E-04	1,81E-01	7,65E-03
GOTERM_BP_FAT	GO:0016042~lipid catabolic process	16	0,8	1,48E-04	2,34E-01	9,83E-03
GOTERM_BP_FAT	GO:0006026~aminoglycan catabolic process	13	0,7	1,92E-04	2,93E-01	1,23E-02
GOTERM_BP_FAT	GO:0006575~cellular amino acid derivative metabolic process	19	1,0	1,96E-04	2,97E-01	1,21E-02
GOTERM_BP_FAT	GO:0019748~secondary metabolic process	21	1,1	2,19E-04	3,27E-01	1,31E-02
GOTERM_BP_FAT	GO:0050830~defense response to Gram-positive bacterium	11	0,6	2,41E-04	3,52E-01	1,39E-02
GOTERM_BP_FAT	GO:0006959~humoral immune response	22	1,1	6,18E-04	6,73E-01	3,43E-02
GOTERM_BP_FAT	GO:0018988~molting cycle, protein-based cuticle	16	0,8	6,82E-04	7,08E-01	3,66E-02
GOTERM_BP_FAT	GO:0042303~molting cycle	16	0,8	6,82E-04	7,08E-01	3,66E-02
GOTERM_BP_FAT	GO:0009309~amine biosynthetic process	15	0,8	8,89E-04	7,99E-01	4,61E-02
GOTERM_BP_FAT	GO:0007591~molting cycle, chitin-based cuticle	15	0,8	8,89E-04	7,99E-01	4,61E-02
GOTERM_BP_FAT	GO:0019730~antimicrobial humoral response	19	1,0	1,21E-03	8,88E-01	6,05E-02
GOTERM_BP_FAT	GO:0044271~nitrogen compound biosynthetic process	45	2,3	1,39E-03	9,18E-01	6,72E-02
GOTERM_BP_FAT	GO:0008652~cellular amino acid biosynthetic process	11	0,6	1,40E-03	9,20E-01	6,61E-02
GOTERM_BP_FAT	GO:0042445~hormone metabolic process	12	0,6	1,48E-03	9,31E-01	6,81E-02
GOTERM_BP_FAT	GO:0010817~regulation of hormone levels	12	0,6	1,48E-03	9,31E-01	6,81E-02
GOTERM_BP_FAT	GO:0046395~carboxylic acid catabolic process	14	0,7	1,83E-03	9,64E-01	8,15E-02
GOTERM_BP_FAT	GO:0016054~organic acid catabolic process	14	0,7	1,83E-03	9,64E-01	8,15E-02
GOTERM_BP_FAT	GO:0006812~cation transport	46	2,4	3,83E-03	9,99E-01	1,59E-01
GOTERM_BP_FAT	GO:0055001~muscle cell development	13	0,7	5,58E-03	1,00E+00	2,18E-01
GOTERM_BP_FAT	GO:0055002~striated muscle cell development	13	0,7	5,58E-03	1,00E+00	2,18E-01
GOTERM_BP_FAT	GO:0006816~calcium ion transport	10	0,5	6,90E-03	1,00E+00	2,57E-01
GOTERM_BP_FAT	GO:0030001~metal ion transport	32	1,6	7,19E-03	1,00E+00	2,61E-01
GOTERM_BP_FAT	GO:0006633~fatty acid biosynthetic process	10	0,5	8,71E-03	1,00E+00	3,02E-01
GOTERM_BP_FAT	GO:0015674~di-, tri-valent inorganic cation transport	11	0,6	1,21E-02	1,00E+00	3,87E-01
GOTERM_BP_FAT	GO:0009063~cellular amino acid catabolic process	10	0,5	1,34E-02	1,00E+00	4,11E-01
GOTERM_BP_FAT	GO:0055082~cellular chemical homeostasis	13	0,7	1,57E-02	1,00E+00	4,55E-01
GOTERM_BP_FAT	GO:0006091~generation of precursor metabolites and energy	38	1,9	1,69E-02	1,00E+00	4,73E-01
GOTERM_BP_FAT	GO:0051146~striated muscle cell differentiation	15	0,8	2,04E-02	1,00E+00	5,32E-01
GOTERM_BP_FAT	GO:0019637~organophosphate metabolic process	20	1,0	2,07E-02	1,00E+00	5,30E-01
GOTERM_BP_FAT	GO:0009310~amine catabolic process	10	0,5	2,35E-02	1,00E+00	5,69E-01
GOTERM_BP_FAT	GO:0016051~carbohydrate biosynthetic process	13	0,7	3,16E-02	1,00E+00	6,72E-01
GOTERM_BP_FAT	GO:0006644~phospholipid metabolic process	17	0,9	4,03E-02	1,00E+00	7,54E-01
GOTERM_BP_FAT	GO:0015672~monovalent inorganic cation transport	30	1,5	4,41E-02	1,00E+00	7,78E-01
GOTERM_BP_FAT	GO:0014706~striated muscle tissue development	10	0,5	4,43E-02	1,00E+00	7,74E-01
GOTERM_BP_FAT	GO:0051606~detection of stimulus	19	1,0	5,06E-02	1,00E+00	8,13E-01
GOTERM_BP_FAT	GO:0060537~muscle tissue development	10	0,5	5,09E-02	1,00E+00	8,09E-01
GOTERM_BP_FAT	GO:0009123~nucleoside monophosphate metabolic process	12	0,6	5,34E-02	1,00E+00	8,19E-01
GOTERM_BP_FAT	GO:0042692~muscle cell differentiation	15	0,8	5,54E-02	1,00E+00	8,25E-01
GOTERM_BP_FAT	GO:0019725~cellular homeostasis	20	1,0	6,97E-02	1,00E+00	8,86E-01
GOTERM_BP_FAT	GO:0009124~nucleoside monophosphate biosynthetic process	11	0,6	7,08E-02	1,00E+00	8,86E-01
GOTERM_BP_FAT	GO:0007606~sensory perception of chemical stimulus	31	1,6	8,67E-02	1,00E+00	9,29E-01
GOTERM_BP_FAT	GO:0034654~nucleobase, nucleoside, nucleotide and nucleic acid biosynthetic process	26	1,3	9,01E-02	1,00E+00	9,33E-01
GOTERM_BP_FAT	GO:0034404~nucleobase, nucleoside and nucleotide biosynthetic process	26	1,3	9,01E-02	1,00E+00	9,33E-01
GOTERM_BP_FAT	GO:0009165~nucleotide biosynthetic process	25	1,3	9,16E-02	1,00E+00	9,33E-01
GOTERM_BP_FAT	GO:0048878~chemical homeostasis	13	0,7	9,24E-02	1,00E+00	9,32E-01
GOTERM_CC_FAT	GO:0005576~extracellular region	166	8,5	1,09E-35	3,00E-33	3,00E-33
GOTERM_CC_FAT	GO:0042598~vesicular fraction	54	2,8	4,04E-26	1,11E-23	5,56E-24
GOTERM_CC_FAT	GO:0005792~microsome	54	2,8	4,04E-26	1,11E-23	5,56E-24
GOTERM_CC_FAT	GO:0005624~membrane fraction	54	2,8	2,89E-21	7,96E-19	2,65E-19
GOTERM_CC_FAT	GO:0005626~insoluble fraction	54	2,8	2,56E-20	7,03E-18	1,76E-18
GOTERM_CC_FAT	GO:0000267~cell fraction	54	2,8	1,20E-19	3,30E-17	6,59E-18
GOTERM_CC_FAT	GO:0019898~extrinsic to membrane	59	3,0	8,56E-16	2,44E-13	4,07E-14
GOTERM_CC_FAT	GO:0005615~extracellular space	31	1,6	2,12E-08	5,82E-06	8,32E-07
GOTERM_CC_FAT	GO:0005783~endoplasmic reticulum	70	3,6	2,14E-08	5,87E-06	7,34E-07
GOTERM_CC_FAT	GO:0043292~contractile fiber	14	0,7	6,32E-08	1,74E-05	1,93E-06
GOTERM_CC_FAT	GO:0044449~contractile fiber part	12	0,6	1,69E-06	4,64E-04	4,65E-05
GOTERM_CC_FAT	GO:0044421~extracellular region part	39	2,0	1,58E-05	4,34E-03	3,95E-04
GOTERM_CC_FAT	GO:0030017~sarcomere	10	0,5	1,95E-05	5,34E-03	4,46E-04
GOTERM_CC_FAT	GO:0030016~myofibril	10	0,5	3,95E-05	1,08E-02	8,36E-04
GOTERM_CC_FAT	GO:0005811~lipid particle	53	2,7	1,24E-04	3,34E-02	2,42E-03
GOTERM_CC_FAT	GO:0016021~integral to membrane	209	10,7	4,07E-04	1,06E-01	7,43E-03
GOTERM_CC_FAT	GO:0031224~intrinsic to membrane	210	10,8	7,57E-04	1,88E-01	1,29E-02

GOTERM_CC_FAT	GO:0005887~integral to plasma membrane	37	1,9	1,43E-02	9,81E-01	2,08E-01
GOTERM_CC_FAT	GO:0031226~intrinsic to plasma membrane	37	1,9	1,78E-02	9,93E-01	2,41E-01
GOTERM_CC_FAT	GO:0045211~postsynaptic membrane	12	0,6	2,17E-02	9,98E-01	2,72E-01
GOTERM_CC_FAT	GO:0005773~vacuole	14	0,7	3,03E-02	1,00E+00	3,45E-01
GOTERM_CC_FAT	GO:0042579~microbody	10	0,5	6,67E-02	1,00E+00	5,95E-01
GOTERM_CC_FAT	GO:0005777~peroxisome	10	0,5	6,67E-02	1,00E+00	5,95E-01
GOTERM_MF_FAT	GO:0030247~polysaccharide binding	62	3,2	3,43E-20	3,04E-17	3,04E-17
GOTERM_MF_FAT	GO:0001871~pattern binding	62	3,2	3,43E-20	3,04E-17	3,04E-17
GOTERM_MF_FAT	GO:0046906~tetrapyrrole binding	66	3,4	9,60E-20	8,50E-17	4,25E-17
GOTERM_MF_FAT	GO:0020037~heme binding	66	3,4	9,60E-20	8,50E-17	4,25E-17
GOTERM_MF_FAT	GO:0005506~iron ion binding	96	4,9	3,04E-19	2,70E-16	8,99E-17
GOTERM_MF_FAT	GO:0008061~chitin binding	53	2,7	9,34E-19	8,27E-16	2,07E-16
GOTERM_MF_FAT	GO:0009055~electron carrier activity	80	4,1	3,50E-18	3,10E-15	6,20E-16
GOTERM_MF_FAT	GO:0008233~peptidase activity	172	8,8	2,32E-16	1,97E-13	3,28E-14
GOTERM_MF_FAT	GO:0042302~structural constituent of cuticle	55	2,8	9,07E-16	7,87E-13	1,12E-13
GOTERM_MF_FAT	GO:0005214~structural constituent of chitin-based cuticle	52	2,7	1,19E-15	1,08E-12	1,35E-13
GOTERM_MF_FAT	GO:0030246~carbohydrate binding	73	3,7	1,54E-15	1,38E-12	1,53E-13
GOTERM_MF_FAT	GO:0008236~serine-type peptidase activity	98	5,0	1,41E-14	1,25E-11	1,25E-12
GOTERM_MF_FAT	GO:0017171~serine hydrolase activity	98	5,0	2,22E-14	1,97E-11	1,79E-12
GOTERM_MF_FAT	GO:0070011~peptidase activity, acting on L-amino acid peptides	158	8,1	6,40E-14	5,67E-11	4,72E-12
GOTERM_MF_FAT	GO:0004252~serine-type endopeptidase activity	89	4,6	2,91E-13	2,58E-10	1,98E-11
GOTERM_MF_FAT	GO:0016490~structural constituent of peritrophic membrane	19	1,0	1,37E-11	1,21E-08	8,67E-10
GOTERM_MF_FAT	GO:0004175~endopeptidase activity	118	6,0	1,67E-10	1,48E-07	9,87E-09
GOTERM_MF_FAT	GO:0004091~carboxylesterase activity	43	2,2	3,04E-10	2,69E-07	1,68E-08
GOTERM_MF_FAT	GO:0005198~structural molecule activity	111	5,7	2,29E-08	2,03E-05	1,19E-06
GOTERM_MF_FAT	GO:0016298~lipase activity	35	1,8	6,42E-08	5,69E-05	3,16E-06
GOTERM_MF_FAT	GO:0008010~structural constituent of chitin-based larval cuticle	20	1,0	4,47E-07	3,96E-04	2,09E-05
GOTERM_MF_FAT	GO:0048037~cofactor binding	53	2,7	1,25E-06	1,11E-03	5,55E-05
GOTERM_MF_FAT	GO:0004180~carboxypeptidase activity	21	1,1	1,42E-06	1,25E-03	5,97E-05
GOTERM_MF_FAT	GO:0004806~triacylglycerol lipase activity	19	1,0	2,44E-06	2,16E-03	9,83E-05
GOTERM_MF_FAT	GO:0019842~vitamin binding	32	1,6	1,77E-05	1,56E-02	6,81E-04
GOTERM_MF_FAT	GO:0008237~metallopeptidase activity	45	2,3	5,52E-05	4,77E-02	2,04E-03
GOTERM_MF_FAT	GO:0008238~exopeptidase activity	32	1,6	7,72E-05	6,61E-02	2,73E-03
GOTERM_MF_FAT	GO:0004620~phospholipase activity	19	1,0	7,72E-05	6,61E-02	2,63E-03
GOTERM_MF_FAT	GO:0016811~hydrolase activity, acting on carbon-nitrogen (but not peptide) bonds, in linear amides	22	1,1	8,65E-05	7,38E-02	2,83E-03
GOTERM_MF_FAT	GO:0015101~organic cation transmembrane transporter activity	16	0,8	1,08E-04	9,16E-02	3,43E-03
GOTERM_MF_FAT	GO:0004866~endopeptidase inhibitor activity	26	1,3	1,17E-04	9,83E-02	3,56E-03
GOTERM_MF_FAT	GO:0050662~coenzyme binding	38	1,9	1,48E-04	1,23E-01	4,35E-03
GOTERM_MF_FAT	GO:0030414~peptidase inhibitor activity	26	1,3	2,24E-04	1,80E-01	6,38E-03
GOTERM_MF_FAT	GO:0004857~enzyme inhibitor activity	30	1,5	6,37E-04	4,31E-01	1,75E-02
GOTERM_MF_FAT	GO:0004867~serine-type endopeptidase inhibitor activity	21	1,1	6,85E-04	4,55E-01	1,82E-02
GOTERM_MF_FAT	GO:0015926~glucosidase activity	10	0,5	6,89E-04	4,57E-01	1,78E-02
GOTERM_MF_FAT	GO:0070279~vitamin B6 binding	15	0,8	7,99E-04	5,08E-01	2,00E-02
GOTERM_MF_FAT	GO:0030170~pyridoxal phosphate binding	15	0,8	7,99E-04	5,08E-01	2,00E-02
GOTERM_MF_FAT	GO:0015370~solute:sodium symporter activity	20	1,0	9,77E-04	5,79E-01	2,38E-02
GOTERM_MF_FAT	GO:0004181~metallocarboxypeptidase activity	11	0,6	2,46E-03	8,87E-01	5,73E-02
GOTERM_MF_FAT	GO:0016877~ligase activity, forming carbon-sulfur bonds	10	0,5	3,52E-03	9,56E-01	7,89E-02
GOTERM_MF_FAT	GO:0015294~solute:cation symporter activity	22	1,1	4,54E-03	9,82E-01	9,83E-02
GOTERM_MF_FAT	GO:0004364~glutathione transferase activity	13	0,7	4,90E-03	9,87E-01	1,03E-01
GOTERM_MF_FAT	GO:0005507~copper ion binding	11	0,6	8,02E-03	9,99E-01	1,60E-01
GOTERM_MF_FAT	GO:0015293~symporter activity	22	1,1	8,27E-03	9,99E-01	1,61E-01
GOTERM_MF_FAT	GO:0015276~ligand-gated ion channel activity	19	1,0	1,03E-02	1,00E+00	1,92E-01
GOTERM_MF_FAT	GO:0022834~ligand-gated channel activity	19	1,0	1,03E-02	1,00E+00	1,92E-01
GOTERM_MF_FAT	GO:0031406~carboxylic acid binding	18	0,9	1,05E-02	1,00E+00	1,91E-01
GOTERM_MF_FAT	GO:0050660~FAD binding	16	0,8	2,70E-02	1,00E+00	4,17E-01
GOTERM_MF_FAT	GO:0005275~amine transmembrane transporter activity	15	0,8	3,71E-02	1,00E+00	5,17E-01
GOTERM_MF_FAT	GO:0016701~oxidoreductase activity, acting on single donors with incorporation of molecular oxygen	11	0,6	4,24E-02	1,00E+00	5,58E-01
GOTERM_MF_FAT	GO:0022836~gated channel activity	25	1,3	4,69E-02	1,00E+00	5,88E-01
GOTERM_MF_FAT	GO:0016765~transferase activity, transferring alkyl or aryl (other than methyl) groups	15	0,8	4,80E-02	1,00E+00	5,89E-01
GOTERM_MF_FAT	GO:0051287~NAD or NADH binding	11	0,6	5,81E-02	1,00E+00	6,54E-01
GOTERM_MF_FAT	GO:0022838~substrate specific channel activity	35	1,8	6,70E-02	1,00E+00	7,00E-01
GOTERM_MF_FAT	GO:0005230~extracellular ligand-gated ion channel activity	13	0,7	6,89E-02	1,00E+00	7,04E-01
GOTERM_MF_FAT	GO:0022803~passive transmembrane transporter activity	36	1,8	7,11E-02	1,00E+00	7,09E-01
GOTERM_MF_FAT	GO:0015267~channel activity	36	1,8	7,11E-02	1,00E+00	7,09E-01
GOTERM_MF_FAT	GO:0005216~ion channel activity	34	1,7	7,17E-02	1,00E+00	7,05E-01
GOTERM_MF_FAT	GO:0043167~ion binding	280	14,3	7,27E-02	1,00E+00	7,04E-01
GOTERM_MF_FAT	GO:0016702~oxidoreductase activity, acting on single donors with incorporation of molecular oxygen, inco	10	0,5	8,13E-02	1,00E+00	7,39E-01
GOTERM_MF_FAT	GO:0015171~amino acid transmembrane transporter activity	12	0,6	8,28E-02	1,00E+00	7,39E-01
GOTERM_MF_FAT	GO:0043169~cation binding	278	14,2	8,46E-02	1,00E+00	7,41E-01
GOTERM_MF_FAT	GO:0008235~metalloexopeptidase activity	11	0,6	8,80E-02	1,00E+00	7,49E-01

Ada2a - Activation						
Category	Term	Number of affected genes	percentage	PValue	Bonferroni	Benjamini
GOTERM_BP_FAT	GO:0055114~oxidation reduction	264	11,0	4,07E-68	7,92E-65	7,92E-65
GOTERM_BP_FAT	GO:0006091~generation of precursor metabolites and energy	107	4,5	2,82E-33	5,49E-30	2,74E-30
GOTERM_BP_FAT	GO:0015980~energy derivation by oxidation of organic compounds	65	2,7	8,90E-25	1,73E-21	5,77E-22
GOTERM_BP_FAT	GO:0045333~cellular respiration	60	2,5	5,85E-23	1,14E-19	2,85E-20
GOTERM_BP_FAT	GO:0006119~oxidative phosphorylation	67	2,8	8,83E-22	1,72E-18	3,43E-19
GOTERM_BP_FAT	GO:0006030~chitin metabolic process	61	2,5	2,27E-21	4,42E-18	7,37E-19
GOTERM_BP_FAT	GO:0022900~electron transport chain	52	2,2	3,10E-20	6,03E-17	8,62E-18
GOTERM_BP_FAT	GO:0005976~polysaccharide metabolic process	70	2,9	1,51E-19	2,93E-16	3,66E-17
GOTERM_BP_FAT	GO:0022904~respiratory electron transport chain	45	1,9	9,51E-19	1,85E-15	2,05E-16
GOTERM_BP_FAT	GO:0042775~mitochondrial ATP synthesis coupled electron transport	42	1,8	3,35E-18	6,52E-15	6,52E-16
GOTERM_BP_FAT	GO:0042773~ATP synthesis coupled electron transport	43	1,8	7,70E-18	1,50E-14	1,36E-15
GOTERM_BP_FAT	GO:0006022~aminoglycan metabolic process	64	2,7	1,60E-17	3,12E-14	2,60E-15
GOTERM_BP_FAT	GO:0019318~hexose metabolic process	39	1,6	3,86E-11	7,50E-08	5,77E-09
GOTERM_BP_FAT	GO:0006120~mitochondrial electron transport, NADH to ubiquinone	23	1,0	5,01E-11	9,75E-08	6,96E-09
GOTERM_BP_FAT	GO:0006508~proteolysis	171	7,1	2,26E-10	4,40E-07	2,93E-08
GOTERM_BP_FAT	GO:0005996~monosaccharide metabolic process	41	1,7	2,47E-10	4,80E-07	3,00E-08
GOTERM_BP_FAT	GO:0006631~fatty acid metabolic process	28	1,2	7,13E-10	1,39E-06	8,16E-08
GOTERM_BP_FAT	GO:0051186~cofactor metabolic process	41	1,7	5,56E-09	1,08E-05	6,00E-07
GOTERM_BP_FAT	GO:0006811~ion transport	92	3,8	1,30E-08	2,53E-05	1,33E-06
GOTERM_BP_FAT	GO:0046394~carboxylic acid biosynthetic process	29	1,2	2,63E-08	5,11E-05	2,55E-06
GOTERM_BP_FAT	GO:0016053~organic acid biosynthetic process	29	1,2	2,63E-08	5,11E-05	2,55E-06
GOTERM_BP_FAT	GO:0006633~fatty acid biosynthetic process	19	0,8	3,52E-08	6,85E-05	3,26E-06
GOTERM_BP_FAT	GO:0006732~coenzyme metabolic process	35	1,5	3,92E-08	7,62E-05	3,46E-06
GOTERM_BP_FAT	GO:0015672~monovalent inorganic cation transport	54	2,3	1,34E-07	2,61E-04	1,13E-05
GOTERM_BP_FAT	GO:0006006~glucose metabolic process	27	1,1	1,57E-07	3,05E-04	1,27E-05
GOTERM_BP_FAT	GO:0006818~hydrogen transport	27	1,1	4,93E-07	9,58E-04	3,83E-05
GOTERM_BP_FAT	GO:0006812~cation transport	68	2,8	7,46E-07	1,45E-03	5,58E-05
GOTERM_BP_FAT	GO:0015992~proton transport	26	1,1	1,38E-06	2,67E-03	9,91E-05
GOTERM_BP_FAT	GO:0044271~nitrogen compound biosynthetic process	63	2,6	1,88E-06	3,65E-03	1,31E-04
GOTERM_BP_FAT	GO:0006084~acetyl-CoA metabolic process	18	0,8	2,51E-06	4,88E-03	1,69E-04
GOTERM_BP_FAT	GO:0044275~cellular carbohydrate catabolic process	22	0,9	4,78E-06	9,26E-03	3,10E-04
GOTERM_BP_FAT	GO:0009109~coenzyme catabolic process	17	0,7	5,13E-06	9,93E-03	3,22E-04
GOTERM_BP_FAT	GO:0009060~aerobic respiration	17	0,7	8,36E-06	1,61E-02	5,08E-04
GOTERM_BP_FAT	GO:0051187~cofactor catabolic process	17	0,7	8,36E-06	1,61E-02	5,08E-04
GOTERM_BP_FAT	GO:0006099~tricarboxylic acid cycle	16	0,7	1,70E-05	3,26E-02	1,00E-03
GOTERM_BP_FAT	GO:0046356~acetyl-CoA catabolic process	16	0,7	1,70E-05	3,26E-02	1,00E-03
GOTERM_BP_FAT	GO:0046164~alcohol catabolic process	21	0,9	1,92E-05	3,66E-02	1,10E-03
GOTERM_BP_FAT	GO:0015986~ATP synthesis coupled proton transport	22	0,9	2,67E-05	5,05E-02	1,48E-03
GOTERM_BP_FAT	GO:0015985~energy coupled proton transport, down electrochemical gradient	22	0,9	2,67E-05	5,05E-02	1,48E-03
GOTERM_BP_FAT	GO:0016052~carbohydrate catabolic process	28	1,2	4,02E-05	7,52E-02	2,17E-03
GOTERM_BP_FAT	GO:0034220~ion transmembrane transport	22	0,9	4,91E-05	9,10E-02	2,58E-03
GOTERM_BP_FAT	GO:0019748~secondary metabolic process	25	1,0	6,87E-05	1,25E-01	3,51E-03
GOTERM_BP_FAT	GO:0006007~glucose catabolic process	18	0,8	7,13E-05	1,29E-01	3,55E-03
GOTERM_BP_FAT	GO:0019320~hexose catabolic process	18	0,8	7,13E-05	1,29E-01	3,55E-03
GOTERM_BP_FAT	GO:0046365~monosaccharide catabolic process	18	0,8	1,00E-04	1,77E-01	4,86E-03
GOTERM_BP_FAT	GO:0006096~glycolysis	15	0,6	2,67E-04	4,05E-01	1,26E-02
GOTERM_BP_FAT	GO:0043603~cellular amide metabolic process	10	0,4	2,77E-04	4,17E-01	1,28E-02
GOTERM_BP_FAT	GO:0006754~ATP biosynthetic process	25	1,0	2,78E-04	4,18E-01	1,25E-02
GOTERM_BP_FAT	GO:0009636~response to toxin	12	0,5	2,97E-04	4,39E-01	1,31E-02
GOTERM_BP_FAT	GO:0016310~phosphorylation	90	3,8	3,72E-04	5,15E-01	1,60E-02
GOTERM_BP_FAT	GO:0046034~ATP metabolic process	25	1,0	4,23E-04	5,61E-01	1,77E-02
GOTERM_BP_FAT	GO:0016042~lipid catabolic process	17	0,7	4,85E-04	6,11E-01	1,99E-02
GOTERM_BP_FAT	GO:0006163~purine nucleotide metabolic process	35	1,5	5,50E-04	6,57E-01	2,21E-02
GOTERM_BP_FAT	GO:0006164~purine nucleotide biosynthetic process	34	1,4	5,51E-04	6,58E-01	2,16E-02
GOTERM_BP_FAT	GO:0009145~purine nucleoside triphosphate biosynthetic process	25	1,0	6,31E-04	7,07E-01	2,43E-02
GOTERM_BP_FAT	GO:0009206~purine ribonucleoside triphosphate biosynthetic process	25	1,0	6,31E-04	7,07E-01	2,43E-02
GOTERM_BP_FAT	GO:0044242~cellular lipid catabolic process	12	0,5	6,91E-04	7,39E-01	2,60E-02
GOTERM_BP_FAT	GO:0009152~purine ribonucleotide biosynthetic process	29	1,2	7,28E-04	7,57E-01	2,69E-02
GOTERM_BP_FAT	GO:0009260~ribonucleotide biosynthetic process	30	1,3	7,39E-04	7,63E-01	2,68E-02
GOTERM_BP_FAT	GO:0009142~nucleoside triphosphate biosynthetic process	25	1,0	7,65E-04	7,74E-01	2,72E-02
GOTERM_BP_FAT	GO:0009201~ribonucleoside triphosphate biosynthetic process	25	1,0	7,65E-04	7,74E-01	2,72E-02
GOTERM_BP_FAT	GO:0009165~nucleotide biosynthetic process	38	1,6	1,21E-03	9,05E-01	4,19E-02
GOTERM_BP_FAT	GO:0034654~nucleobase, nucleoside, nucleotide and nucleic acid biosynthetic process	39	1,6	1,51E-03	9,47E-01	5,12E-02
GOTERM_BP_FAT	GO:0034404~nucleobase, nucleoside and nucleotide biosynthetic process	39	1,6	1,51E-03	9,47E-01	5,12E-02
GOTERM_BP_FAT	GO:0016054~organic acid catabolic process	16	0,7	1,55E-03	9,51E-01	5,14E-02
GOTERM_BP_FAT	GO:0046395~carboxylic acid catabolic process	16	0,7	1,55E-03	9,51E-01	5,14E-02
GOTERM_BP_FAT	GO:0009205~purine ribonucleoside triphosphate metabolic process	25	1,0	1,58E-03	9,53E-01	5,15E-02
GOTERM_BP_FAT	GO:0009144~purine nucleoside triphosphate metabolic process	25	1,0	1,58E-03	9,53E-01	5,15E-02
GOTERM_BP_FAT	GO:0055085~transmembrane transport	30	1,3	1,61E-03	9,57E-01	5,19E-02
GOTERM_BP_FAT	GO:0009259~ribonucleotide metabolic process	30	1,3	1,61E-03	9,57E-01	5,19E-02
GOTERM_BP_FAT	GO:0009150~purine ribonucleotide metabolic process	29	1,2	1,62E-03	9,58E-01	5,13E-02
GOTERM_BP_FAT	GO:0009199~ribonucleoside triphosphate metabolic process	25	1,0	1,87E-03	9,74E-01	5,79E-02
GOTERM_BP_FAT	GO:0051188~cofactor biosynthetic process	19	0,8	2,14E-03	9,85E-01	6,51E-02
GOTERM_BP_FAT	GO:0009141~nucleoside triphosphate metabolic process	25	1,0	2,20E-03	9,86E-01	6,58E-02
GOTERM_BP_FAT	GO:0030001~metal ion transport	39	1,6	3,42E-03	9,99E-01	9,89E-02
GOTERM_BP_FAT	GO:0055002~striated muscle cell development	15	0,6	4,50E-03	1,00E+00	1,26E-01
GOTERM_BP_FAT	GO:0055001~muscle cell development	15	0,6	4,50E-03	1,00E+00	1,26E-01
GOTERM_BP_FAT	GO:0006952~defense response	40	1,7	4,51E-03	1,00E+00	1,25E-01
GOTERM_BP_FAT	GO:0034637~cellular carbohydrate biosynthetic process	10	0,4	5,59E-03	1,00E+00	1,50E-01
GOTERM_BP_FAT	GO:0016051~carbohydrate biosynthetic process	17	0,7	6,27E-03	1,00E+00	1,65E-01
GOTERM_BP_FAT	GO:0006575~cellular amino acid derivative metabolic process	18	0,8	6,47E-03	1,00E+00	1,67E-01
GOTERM_BP_FAT	GO:0010817~regulation of hormone levels	12	0,5	8,20E-03	1,00E+00	2,04E-01
GOTERM_BP_FAT	GO:0042445~hormone metabolic process	12	0,5	8,20E-03	1,00E+00	2,04E-01
GOTERM_BP_FAT	GO:0009064~glutamine family amino acid metabolic process	11	0,5	9,17E-03	1,00E+00	2,23E-01
GOTERM_BP_FAT	GO:0006796~phosphate metabolic process	103	4,3	9,63E-03	1,00E+00	2,30E-01
GOTERM_BP_FAT	GO:0006793~phosphorus metabolic process	103	4,3	9,63E-03	1,00E+00	2,30E-01
GOTERM_BP_FAT	GO:0006814~sodium ion transport	20	0,8	1,41E-02	1,00E+00	3,16E-01
GOTERM_BP_FAT	GO:0006790~sulfur metabolic process	12	0,5	1,61E-02	1,00E+00	3,46E-01
GOTERM_BP_FAT	GO:0007606~sensory perception of chemical stimulus	41	1,7	1,85E-02	1,00E+00	3,85E-01
GOTERM_BP_FAT	GO:0009063~cellular amino acid catabolic process	11	0,5	1,86E-02	1,00E+00	3,81E-01
GOTERM_BP_FAT	GO:0009108~coenzyme biosynthetic process	13	0,5	2,03E-02	1,00E+00	4,04E-01

GOTERM_BP_FAT	GO:0008610~lipid biosynthetic process	27	1,1	2,64E-02	1,00E+00	4,87E-01
GOTERM_BP_FAT	GO:0051146~striated muscle cell differentiation	17	0,7	2,66E-02	1,00E+00	4,85E-01
GOTERM_BP_FAT	GO:0045087~innate immune response	22	0,9	2,78E-02	1,00E+00	4,96E-01
GOTERM_BP_FAT	GO:0007608~sensory perception of smell	21	0,9	2,91E-02	1,00E+00	5,08E-01
GOTERM_BP_FAT	GO:0009310~amine catabolic process	11	0,5	3,36E-02	1,00E+00	5,56E-01
GOTERM_BP_FAT	GO:0031032~actomyosin structure organization	11	0,5	4,75E-02	1,00E+00	6,80E-01
GOTERM_BP_FAT	GO:0007519~skeletal muscle tissue development	10	0,4	6,65E-02	1,00E+00	7,97E-01
GOTERM_BP_FAT	GO:0042692~muscle cell differentiation	17	0,7	7,65E-02	1,00E+00	8,38E-01
GOTERM_BP_FAT	GO:0007498~mesoderm development	20	0,8	7,68E-02	1,00E+00	8,36E-01
GOTERM_BP_FAT	GO:0006820~anion transport	12	0,5	8,14E-02	1,00E+00	8,50E-01
GOTERM_BP_FAT	GO:0009309~amine biosynthetic process	12	0,5	8,14E-02	1,00E+00	8,50E-01
GOTERM_CC_FAT	GO:0005739~mitochondrion	192	8,0	2,41E-24	8,10E-22	8,10E-22
GOTERM_CC_FAT	GO:0044429~mitochondrial part	152	6,3	1,16E-23	3,89E-21	1,94E-21
GOTERM_CC_FAT	GO:0042598~vesicular fraction	56	2,3	2,25E-21	7,57E-19	2,52E-19
GOTERM_CC_FAT	GO:0005792~microsome	56	2,3	2,25E-21	7,57E-19	2,52E-19
GOTERM_CC_FAT	GO:0005624~membrane fraction	57	2,4	2,53E-17	8,50E-15	2,13E-15
GOTERM_CC_FAT	GO:0005626~insoluble fraction	57	2,4	2,25E-16	7,46E-14	1,49E-14
GOTERM_CC_FAT	GO:0070469~respiratory chain	48	2,0	1,18E-15	4,10E-13	6,84E-14
GOTERM_CC_FAT	GO:0000267~cell fraction	57	2,4	1,27E-15	4,10E-13	5,86E-14
GOTERM_CC_FAT	GO:0005746~mitochondrial respiratory chain	46	1,9	6,88E-15	2,31E-12	2,89E-13
GOTERM_CC_FAT	GO:0005740~mitochondrial envelope	94	3,9	6,13E-14	2,06E-11	2,29E-12
GOTERM_CC_FAT	GO:0044455~mitochondrial membrane part	60	2,5	1,83E-13	6,13E-11	6,13E-12
GOTERM_CC_FAT	GO:0005576~extracellular region	151	6,3	5,02E-13	1,69E-10	1,53E-11
GOTERM_CC_FAT	GO:0031966~mitochondrial membrane	87	3,6	1,15E-12	3,85E-10	3,21E-11
GOTERM_CC_FAT	GO:0005743~mitochondrial inner membrane	75	3,1	6,49E-12	2,18E-09	1,68E-10
GOTERM_CC_FAT	GO:0005811~lipid particle	86	3,6	2,44E-11	8,21E-09	5,86E-10
GOTERM_CC_FAT	GO:0031980~mitochondrial lumen	61	2,5	1,59E-10	5,33E-08	3,55E-09
GOTERM_CC_FAT	GO:0005759~mitochondrial matrix	61	2,5	1,59E-10	5,33E-08	3,55E-09
GOTERM_CC_FAT	GO:0019866~organelle inner membrane	76	3,2	2,08E-10	6,98E-08	4,36E-09
GOTERM_CC_FAT	GO:0005747~mitochondrial respiratory chain complex I	26	1,1	2,99E-09	1,01E-06	5,92E-08
GOTERM_CC_FAT	GO:0030964~NADH dehydrogenase complex	26	1,1	2,99E-09	1,01E-06	5,92E-08
GOTERM_CC_FAT	GO:0045271~respiratory chain complex I	26	1,1	2,99E-09	1,01E-06	5,92E-08
GOTERM_CC_FAT	GO:0019898~extrinsic to membrane	57	2,4	5,74E-09	1,93E-06	1,07E-07
GOTERM_CC_FAT	GO:0031967~organelle envelope	97	4,1	2,05E-07	6,90E-05	3,63E-06
GOTERM_CC_FAT	GO:0031090~organelle membrane	117	4,9	2,19E-07	7,37E-05	3,68E-06
GOTERM_CC_FAT	GO:0031975~envelope	97	4,1	2,39E-07	8,02E-05	3,82E-06
GOTERM_CC_FAT	GO:0043292~contractile fiber	15	0,6	2,57E-07	8,64E-05	3,93E-06
GOTERM_CC_FAT	GO:0005783~endoplasmic reticulum	80	3,3	3,11E-06	1,04E-03	4,54E-05
GOTERM_CC_FAT	GO:0044449~contractile fiber part	13	0,5	4,30E-06	1,44E-03	6,01E-05
GOTERM_CC_FAT	GO:0005761~mitochondrial ribosome	30	1,3	5,38E-06	1,80E-03	7,23E-05
GOTERM_CC_FAT	GO:0000313~organellar ribosome	30	1,3	5,38E-06	1,80E-03	7,23E-05
GOTERM_CC_FAT	GO:0000314~organellar small ribosomal subunit	16	0,7	3,70E-05	1,23E-02	4,78E-04
GOTERM_CC_FAT	GO:0005763~mitochondrial small ribosomal subunit	16	0,7	3,70E-05	1,23E-02	4,78E-04
GOTERM_CC_FAT	GO:0030016~myofibril	11	0,5	6,47E-05	2,15E-02	8,05E-04
GOTERM_CC_FAT	GO:0016469~proton-transporting two-sector ATPase complex	23	1,0	2,32E-04	7,49E-02	2,78E-03
GOTERM_CC_FAT	GO:0030017~sarcomere	10	0,4	2,39E-04	7,73E-02	2,77E-03
GOTERM_CC_FAT	GO:0005773~vacuole	21	0,9	1,39E-03	3,74E-01	1,55E-02
GOTERM_CC_FAT	GO:0016021~integral to membrane	271	11,3	1,63E-03	4,22E-01	1,75E-02
GOTERM_CC_FAT	GO:0016471~vacuolar proton-transporting V-type ATPase complex	13	0,5	1,88E-03	4,68E-01	1,95E-02
GOTERM_CC_FAT	GO:0033178~proton-transporting two-sector ATPase complex, catalytic domain	13	0,5	1,88E-03	4,68E-01	1,95E-02
GOTERM_CC_FAT	GO:0044437~vacuolar part	14	0,6	2,08E-03	5,04E-01	2,10E-02
GOTERM_CC_FAT	GO:0005774~vacuolar membrane	14	0,6	2,08E-03	5,04E-01	2,10E-02
GOTERM_CC_FAT	GO:0033176~proton-transporting V-type ATPase complex	14	0,6	2,85E-03	6,17E-01	2,78E-02
GOTERM_CC_FAT	GO:0031224~intrinsic to membrane	272	11,4	3,47E-03	6,89E-01	3,28E-02
GOTERM_CC_FAT	GO:0000315~organellar large ribosomal subunit	16	0,7	9,95E-03	9,65E-01	8,91E-02
GOTERM_CC_FAT	GO:0005762~mitochondrial large ribosomal subunit	16	0,7	9,95E-03	9,65E-01	8,91E-02
GOTERM_CC_FAT	GO:0045259~proton-transporting ATP synthase complex	11	0,5	1,46E-02	9,93E-01	1,25E-01
GOTERM_CC_FAT	GO:0005777~peroxisome	14	0,6	1,68E-02	9,97E-01	1,39E-01
GOTERM_CC_FAT	GO:0042579~microbody	14	0,6	1,68E-02	9,97E-01	1,39E-01
GOTERM_CC_FAT	GO:0015935~small ribosomal subunit	19	0,8	3,12E-02	1,00E+00	2,39E-01
GOTERM_CC_FAT	GO:0005615~extracellular space	22	0,9	3,48E-02	1,00E+00	2,58E-01
GOTERM_CC_FAT	GO:0044421~extracellular region part	34	1,4	8,11E-02	1,00E+00	5,00E-01
GOTERM_CC_FAT	GO:0033177~proton-transporting two-sector ATPase complex, proton-transporting domain	10	0,4	8,59E-02	1,00E+00	5,13E-01
GOTERM_MF_FAT	GO:0009055~electron carrier activity	99	4,1	8,74E-24	8,72E-21	8,72E-21
GOTERM_MF_FAT	GO:0005506~iron ion binding	107	4,5	1,74E-18	1,74E-15	8,68E-16
GOTERM_MF_FAT	GO:0046906~tetrapyrrole binding	70	2,9	1,47E-17	1,46E-14	4,88E-15
GOTERM_MF_FAT	GO:0020037~heme binding	70	2,9	1,47E-17	1,46E-14	4,88E-15
GOTERM_MF_FAT	GO:0008061~chitin binding	56	2,3	5,63E-17	1,11E-13	2,76E-14
GOTERM_MF_FAT	GO:0042302~structural constituent of cuticle	59	2,5	2,16E-14	2,16E-11	4,32E-12
GOTERM_MF_FAT	GO:0005198~structural molecule activity	148	6,2	3,24E-14	3,23E-11	5,39E-12
GOTERM_MF_FAT	GO:0005214~structural constituent of chitin-based cuticle	55	2,3	7,64E-14	7,63E-11	1,09E-11
GOTERM_MF_FAT	GO:0001871~pattern binding	59	2,5	1,33E-13	1,32E-10	1,65E-11
GOTERM_MF_FAT	GO:0030247~polysaccharide binding	59	2,5	1,33E-13	1,32E-10	1,65E-11
GOTERM_MF_FAT	GO:0030246~carbohydrate binding	74	3,1	2,33E-11	2,33E-08	2,59E-09
GOTERM_MF_FAT	GO:0048037~cofactor binding	72	3,0	3,53E-11	3,51E-08	3,51E-09
GOTERM_MF_FAT	GO:0017171~serine hydrolase activity	102	4,3	2,26E-10	2,25E-07	2,05E-08
GOTERM_MF_FAT	GO:0008236~serine-type peptidase activity	101	4,2	3,47E-10	3,46E-07	2,88E-08
GOTERM_MF_FAT	GO:0008233~peptidase activity	176	7,3	2,44E-09	2,43E-06	1,87E-07
GOTERM_MF_FAT	GO:0004252~serine-type endopeptidase activity	91	3,8	4,81E-09	4,80E-06	3,43E-07
GOTERM_MF_FAT	GO:0008010~structural constituent of chitin-based larval cuticle	24	1,0	1,30E-08	1,29E-05	8,63E-07
GOTERM_MF_FAT	GO:0070011~peptidase activity, acting on L-amino acid peptides	164	6,8	2,68E-08	2,67E-05	1,67E-06
GOTERM_MF_FAT	GO:0003954~NADH dehydrogenase activity	25	1,0	2,79E-08	2,78E-05	1,64E-06
GOTERM_MF_FAT	GO:0016651~oxidoreductase activity, acting on NADH or NADPH	28	1,2	5,09E-08	5,07E-05	2,82E-06
GOTERM_MF_FAT	GO:0016490~structural constituent of peritrophic membrane	17	0,7	8,00E-08	7,97E-05	4,20E-06
GOTERM_MF_FAT	GO:0004175~endopeptidase activity	128	5,3	8,15E-08	8,13E-05	4,06E-06
GOTERM_MF_FAT	GO:0050662~coenzyme binding	52	2,2	1,17E-07	1,16E-04	5,54E-06
GOTERM_MF_FAT	GO:0004091~carboxylesterase activity	43	1,8	1,79E-07	1,78E-04	8,10E-06
GOTERM_MF_FAT	GO:0050136~NADH dehydrogenase (quinone) activity	21	0,9	4,52E-07	4,50E-04	1,96E-05
GOTERM_MF_FAT	GO:0016655~oxidoreductase activity, acting on NADH or NADPH, quinone or similar compound as accepto	21	0,9	4,52E-07	4,50E-04	1,96E-05
GOTERM_MF_FAT	GO:0008137~NADH dehydrogenase (ubiquinone) activity	21	0,9	4,52E-07	4,50E-04	1,96E-05
GOTERM_MF_FAT	GO:0015078~hydrogen ion transmembrane transporter activity	38	1,6	1,20E-06	1,19E-03	4,98E-05
GOTERM_MF_FAT	GO:0019842~vitamin binding	39	1,6	1,32E-06	1,32E-03	5,27E-05
GOTERM_MF_FAT	GO:0015077~monovalent inorganic cation transmembrane transporter activity	38	1,6	2,17E-06	2,16E-03	8,31E-05
GOTERM_MF_FAT	GO:0004364~glutathione transferase activity	20	0,8	4,26E-06	4,24E-03	1,57E-04
GOTERM_MF_FAT	GO:0015020~glucuronosyltransferase activity	19	0,8	4,34E-06	4,32E-03	1,55E-04

GOTERM_MF_FAT	GO:0016765~transferase activity, transferring alkyl or aryl (other than methyl) groups	27	1,1	4,65E-06	4,63E-03	1,60E-04
GOTERM_MF_FAT	GO:0022890~inorganic cation transmembrane transporter activity	45	1,9	6,37E-06	6,34E-03	2,12E-04
GOTERM_MF_FAT	GO:0008553~hydrogen-exporting ATPase activity, phosphorylative mechanism	22	0,9	9,44E-05	8,98E-02	3,03E-03
GOTERM_MF_FAT	GO:0030170~pyridoxal phosphate binding	18	0,8	1,90E-04	1,73E-01	5,90E-03
GOTERM_MF_FAT	GO:0070279~vitamin B6 binding	18	0,8	1,90E-04	1,73E-01	5,90E-03
GOTERM_MF_FAT	GO:0004035~alkaline phosphatase activity	10	0,4	3,48E-04	2,93E-01	1,05E-02
GOTERM_MF_FAT	GO:0043492~ATPase activity, coupled to movement of substances	42	1,8	3,65E-04	3,05E-01	1,06E-02
GOTERM_MF_FAT	GO:0042626~ATPase activity, coupled to transmembrane movement of substances	42	1,8	3,65E-04	3,05E-01	1,06E-02
GOTERM_MF_FAT	GO:0016298~lipase activity	31	1,3	4,21E-04	3,43E-01	1,19E-02
GOTERM_MF_FAT	GO:0016820~hydrolase activity, acting on acid anhydrides, catalyzing transmembrane movement of substa	42	1,8	5,10E-04	3,98E-01	1,40E-02
GOTERM_MF_FAT	GO:0031406~carboxylic acid binding	24	1,0	6,34E-04	4,69E-01	1,70E-02
GOTERM_MF_FAT	GO:0004806~triacylglycerol lipase activity	17	0,7	6,67E-04	4,86E-01	1,73E-02
GOTERM_MF_FAT	GO:0015926~glucosidase activity	11	0,5	6,72E-04	4,88E-01	1,70E-02
GOTERM_MF_FAT	GO:0015370~solute:sodium symporter activity	23	1,0	7,91E-04	5,46E-01	1,95E-02
GOTERM_MF_FAT	GO:0008237~metallopeptidase activity	48	2,0	9,04E-04	5,94E-01	2,18E-02
GOTERM_MF_FAT	GO:0051287~NAD or NADH binding	17	0,7	9,27E-04	6,04E-01	2,18E-02
GOTERM_MF_FAT	GO:0015662~ATPase activity, coupled to transmembrane movement of ions, phosphorylative mechanism	26	1,1	1,03E-03	6,42E-01	2,36E-02
GOTERM_MF_FAT	GO:0015405~P-P-bond-hydrolysis-driven transmembrane transporter activity	45	1,9	1,25E-03	7,13E-01	2,79E-02
GOTERM_MF_FAT	GO:0015399~primary active transmembrane transporter activity	45	1,9	1,25E-03	7,13E-01	2,79E-02
GOTERM_MF_FAT	GO:0042625~ATPase activity, coupled to transmembrane movement of ions	26	1,1	2,35E-03	9,04E-01	5,07E-02
GOTERM_MF_FAT	GO:0019829~cation-transporting ATPase activity	13	0,5	2,85E-03	9,42E-01	6,00E-02
GOTERM_MF_FAT	GO:0004180~carboxypeptidase activity	17	0,7	3,88E-03	9,79E-01	7,91E-02
GOTERM_MF_FAT	GO:0050660~FAD binding	21	0,9	3,93E-03	9,80E-01	7,86E-02
GOTERM_MF_FAT	GO:0046961~proton-transporting ATPase activity, rotational mechanism	11	0,5	4,14E-03	9,84E-01	8,10E-02
GOTERM_MF_FAT	GO:0015294~solute:cation symporter activity	25	1,0	6,08E-03	9,98E-01	1,14E-01
GOTERM_MF_FAT	GO:0004620~phospholipase activity	17	0,7	7,90E-03	1,00E+00	1,44E-01
GOTERM_MF_FAT	GO:0008509~anion transmembrane transporter activity	27	1,1	1,03E-02	1,00E+00	1,79E-01
GOTERM_MF_FAT	GO:0015293~symporter activity	25	1,0	1,16E-02	1,00E+00	1,97E-01
GOTERM_MF_FAT	GO:0004222~metalloendopeptidase activity	24	1,0	1,23E-02	1,00E+00	2,04E-01
GOTERM_MF_FAT	GO:0016877~ligase activity, forming carbon-sulfur bonds	10	0,4	1,43E-02	1,00E+00	2,30E-01
GOTERM_MF_FAT	GO:0005549~odorant binding	33	1,4	1,90E-02	1,00E+00	2,90E-01
GOTERM_MF_FAT	GO:0016811~hydrolase activity, acting on carbon-nitrogen (but not peptide) bonds, in linear amides	19	0,8	1,92E-02	1,00E+00	2,88E-01
GOTERM_MF_FAT	GO:0015101~organic cation transmembrane transporter activity	13	0,5	2,51E-02	1,00E+00	3,53E-01
GOTERM_MF_FAT	GO:0015296~anion:cation symporter activity	13	0,5	2,51E-02	1,00E+00	3,53E-01
GOTERM_MF_FAT	GO:0016701~oxidoreductase activity, acting on single donors with incorporation of molecular oxygen	13	0,5	3,08E-02	1,00E+00	4,10E-01
GOTERM_MF_FAT	GO:0051536~iron-sulfur cluster binding	16	0,7	3,19E-02	1,00E+00	4,16E-01
GOTERM_MF_FAT	GO:0051540~metal cluster binding	16	0,7	3,19E-02	1,00E+00	4,16E-01
GOTERM_MF_FAT	GO:0004656~procollagen-proline 4-dioxygenase activity	10	0,4	3,29E-02	1,00E+00	4,21E-01
GOTERM_MF_FAT	GO:0019798~procollagen-proline dioxygenase activity	10	0,4	3,29E-02	1,00E+00	4,21E-01
GOTERM_MF_FAT	GO:0008238~exopeptidase activity	28	1,2	3,44E-02	1,00E+00	4,31E-01
GOTERM_MF_FAT	GO:0031545~peptidyl-proline 4-dioxygenase activity	10	0,4	4,16E-02	1,00E+00	4,90E-01
GOTERM_MF_FAT	GO:0031543~peptidyl-proline dioxygenase activity	10	0,4	4,16E-02	1,00E+00	4,90E-01
GOTERM_MF_FAT	GO:0031418~L-ascorbic acid binding	10	0,4	4,16E-02	1,00E+00	4,90E-01
GOTERM_MF_FAT	GO:0016706~oxidoreductase activity, acting on paired donors, with incorporation or reduction of molecula	13	0,5	4,50E-02	1,00E+00	5,12E-01
GOTERM_MF_FAT	GO:0015300~solute:solute antiporter activity	10	0,4	5,19E-02	1,00E+00	5,58E-01
GOTERM_MF_FAT	GO:0016702~oxidoreductase activity, acting on single donors with incorporation of molecular oxygen, inco	12	0,5	5,69E-02	1,00E+00	5,87E-01
GOTERM_MF_FAT	GO:0015297~antiporter activity	10	0,4	6,36E-02	1,00E+00	6,24E-01
GOTERM_MF_FAT	GO:0022803~passive transmembrane transporter activity	43	1,8	7,07E-02	1,00E+00	6,59E-01
GOTERM_MF_FAT	GO:0015267~channel activity	43	1,8	7,07E-02	1,00E+00	6,59E-01
GOTERM_MF_FAT	GO:0022838~substrate specific channel activity	41	1,7	8,69E-02	1,00E+00	7,31E-01
GOTERM_MF_FAT	GO:0022836~gated channel activity	28	1,2	9,08E-02	1,00E+00	7,42E-01
